# Solvent-Assisted Catalysis: Water Promotes Ring Closure Formation of 2‑Oxazolidinone from Amino Acid Derived *N*‑Boc-Epoxide

**DOI:** 10.1021/acsorginorgau.5c00051

**Published:** 2025-07-17

**Authors:** Patrick de L. Barbosa, Victor Facchinetti, Claudia Regina B. Gomes, Marcus Vinícius de Souza, Thatyana R. A. Vasconcelos, Rodolfo G. Fiorot

**Affiliations:** † Department of Organic Chemistry, Institute of Chemistry, Universidade Federal Fluminense—UFF, Niteroi, Rio de Janeiro 24020-141, Brazil; ‡ Department of Drug and Bioactive Synthesis, Institute of Drug Technology (Farmanguinhos), Fundação Oswaldo Cruz—Fiocruz, Rio de Janeiro, Rio de Janeiro 21041-250, Brazil

**Keywords:** anchimeric assistance, computational chemistry, DFT, intramolecular cyclization, hydrogen bonding, reaction mechanism

## Abstract

Oxazolidinones are important heterocyclic compounds with several therapeutic properties, especially antimicrobial activity, as seen in linezolid. Unexpectedly, a 2-oxazolidinone structurally similar to linezolid was obtained, prompting optimization of the reaction methodology and quantum calculations to rationalize the experimental observation. The reaction proceeded efficiently in refluxing water, in which computational analysis identified a remarkable and unprecedented stable reaction intermediate, enabling possible anchimeric assistance. Water significantly accelerates the process by lowering energy barriers and facilitating epoxide ring-opening through hydrogen bonding catalysis, as supported by density functional theory calculations.

## Graphical abstract

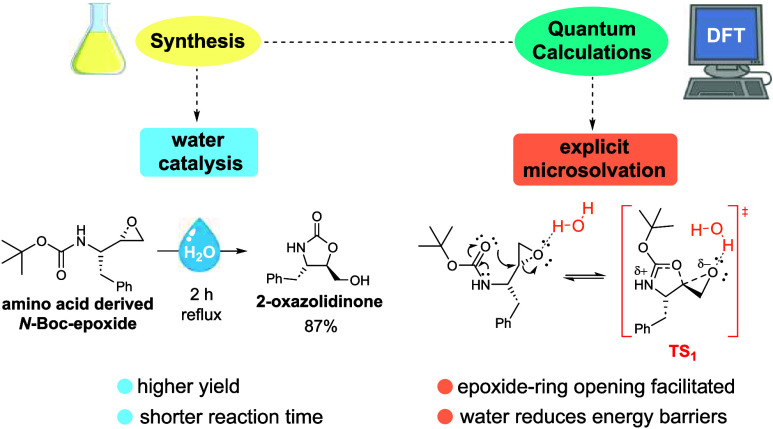

## Introduction

Oxazolidinones are five-membered heterocyclic compounds containing oxygen, nitrogen, and a carbonyl group, existing as three structural isomers (Figure 1a).^1^ Among these, 2-oxazolidinone, also known as cyclic carbamate, stands out in medicinal chemistry due to its structural similarity to pharmacologically significant groups like ureas and amides, making it a promising scaffold for bioactive compounds.^1–3^ Derivatives of oxazolidinones have demonstrated a wide range of therapeutic activities, including anticancer, antiviral, anti-inflammatory, and particularly antimicrobial properties, with a focus on bacterial infections (Figure 1b).^1,2,4^

**Figure 1:**
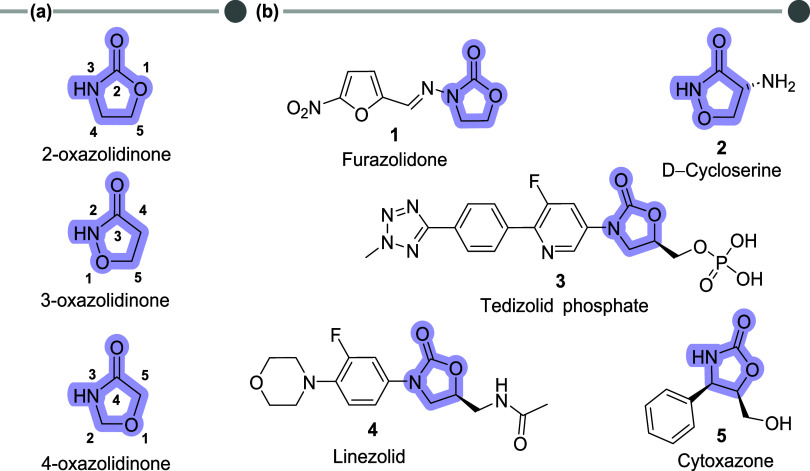
(a) Chemical structures of oxazolidinone isomers; (b) drug molecules and bioactive products containing the oxazolidinone scaffold.

Several notable oxazolidinone derivatives include furazolidone **1**, cycloserine **2**, and tedizolid phosphate **3**, each with distinct applications.^5–7^ Furazolidone, a synthetic nitrofuran, has been used for decades to treat gastrointestinal infections.^5^ d–Cycloserine exhibits potent antituberculosis activity and is employed in multidrug-resistant tuberculosis cases.^6^ Tedizolid phosphate, a newer antibiotic, is used for acute bacterial skin and soft tissue infections caused by Gram-positive resistant bacteria.^7,8^ Linezolid **4**, another synthetic antibiotic in the *N*-aryl-oxazolidinone class, is highly effective against multidrug-resistant Gram-positive pathogens and has been included by the World Health Organization (WHO) in its list of recommended drugs for multidrug-resistant tuberculosis, underscoring the importance of continued exploration of oxazolidinone derivatives.^9–11^ More recently, the natural oxazolidinone derivative cytoxazone **5**, produced by *Streptomyces sp*., was discovered and demonstrated cytokine-modulating activity, showing potential in treating immunological disorders like allergies.^12^

2-oxazolidinones are accessible through various synthetic methods, such as reactions of β-amino alcohols with phosgene (Scheme 1a), cycloaddition of epoxides with isocyanates (Scheme 1b), or an acid-catalyzed intramolecular cyclization of *N*-Boc-epoxides (Scheme 1c).^13–17^ The latter is particularly promising as the Boc (*tert*-butyl carbamate) group acts as a nitrogen-protecting group, minimizing side reactions and improving efficiency despite its low reactivity.^14,18^ These advancements highlight the versatility and therapeutic potential of oxazolidinones.

**Scheme 1:**
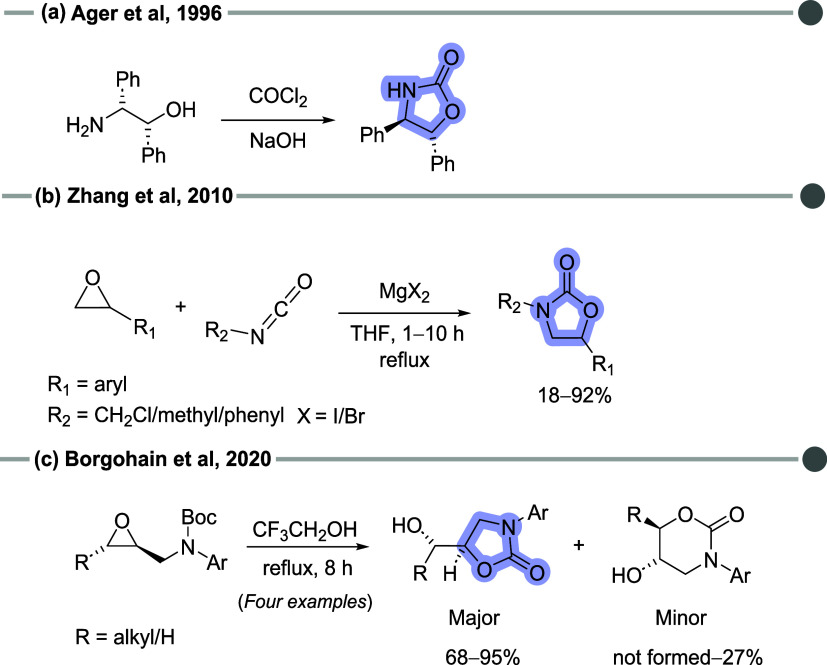
Common Methods for Obtaining 2-Oxazolidinone Derivatives: (a) Reaction of a β-Amino Alcohol with Phosgene (COCl_2_); (b) Cycloaddition of Epoxides with Isocyanates; (c) Acid-Catalyzed Intramolecular Cyclization of N-Boc-Epoxides

Recently, we identified the unexpected formation of a 2-oxazolidinone—structurally similar to linezolid **4** and cytoxazone **5**—as a byproduct of the reaction between a phenylalanine-derived *N*-Boc-epoxide and amines, in isopropanol under reflux conditions, without the addition of acids (Scheme 2a).^19^ Lisauskaitė and co-workers have previously reported this same unanticipated reaction while searching for novel antimalarial chemical probes.^20^ Given that this five-membered heterocycle is a valuable scaffold in medicinal and synthetic chemistry, and considering its acid-free formation from *N*-Boc-epoxides, we focused on optimizing the reaction conditions to improve its yield and reduce the reaction time. For that purpose, amines were omitted, and the reaction was conducted in solvents of varying polarities, including polar aprotic and protic solvents such as water (Scheme 2b). These protic solvents have previously been reported to act as catalysts or cocatalysts in epoxide ring-opening reactions, potentially facilitating the formation of oxazolidinones.^21–24^

**Scheme 2:**
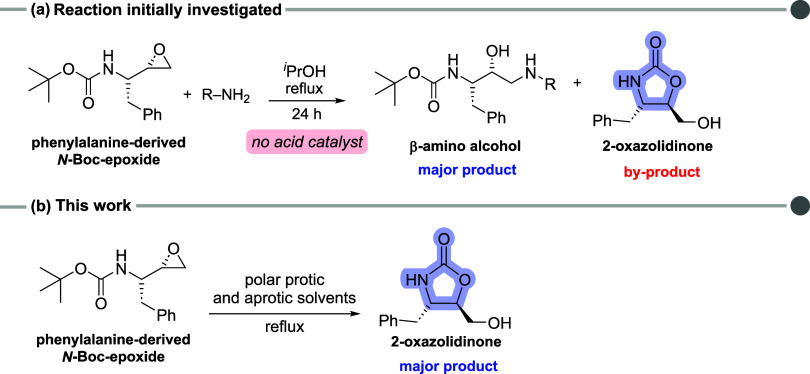
(a) Reaction Conditions of β-Amino Alcohol Formation through Epoxide Aminolysis with 2-Oxazolidinone as a By-Product. (b) Reaction Investigation Proposed by This Work

Furthermore, to the best of our knowledge, the only computational study investigating the formation of 2-oxazolidinones via the intramolecular cyclization of *N*-Boc-epoxides was conducted by Casado-Bellver and co-workers in the early 2000s.^25^ Molecular modeling has proven invaluable in understanding reaction mechanisms, supporting experimental findings, and guiding the development of efficient synthetic methodologies for new bioactive compounds.^26^ To address the limited molecular-level understanding of 2-oxazolidinone formation through this pathway, we performed quantum chemical calculations to examine in detail how reaction conditions influence the energy profiles of the reaction pathways, as well as the roles of both the substrate and solvent in this transformation.

Thus, the reaction pathways were initially evaluated in the gas phase to explore the intrinsic reactivity of the system. Subsequently, solvation effects were introduced using an implicit solvation model, aiming to account for charge density stabilization by the bulk properties of solvents with low and high polarity. Finally, an explicit polar protic solvent model was employed to further stabilize charged intermediates, facilitate proton transfer along the reaction pathway, and elucidate the potential role of solvent-assisted catalysis in the formation of this heterocycle.

## Methodology

### Experimental Section

All reagents and solvents were obtained from commercial suppliers and used without further purification. Reactions were monitored by thin-layer chromatography (TLC) on silica-gel precoated F254 Merck plates (Darmstadt, Germany) visualized under UV light (254–366 nm). The melting point was determined using a PF 1500 Pharma (Gehaka, São Paulo, Brazil) instrument and is uncorrected. The Infrared (IR) spectrum was obtained using a Thermo Scientific Nicolet 6700 spectrometer (Waltham), and frequencies are expressed in cm^–1^. Nuclear magnetic resonance (NMR) spectra were obtained using 400 or 500 MHz Bruker (Billerica) AC spectrometers in dimethyl sulfoxide (DMSO-*d*_6_) or methanol (CD_3_OD) (Cambridge Isotope Laboratories Inc., Tewksbury), as the deuterated solvent. Chemical shifts (δ) are reported in parts per million (ppm). High-resolution mass spectra (HRMS) ESI-TOF (Electrospray Ionization, Time of Flight) in positive ion mode was acquired on a Bruker (Billerica) compact-TOF. The sample was introduced by the standard direct insertion probe method.

### Theoretical Calculations

Full geometry optimizations and frequency calculations were performed using the hybrid meta-exchange-correlation functional M06–2X,^27^ associated with def2-TZVP basis set,^28^ at the Gaussian 09 ^29^ software package with the default convergence criteria and ultrafine integration grid sizes (99 radial, 590 angular points). The M06–2X functional was chosen due to its extensive application in reaction mechanism investigations,^30–32^ especially for its capability in predicting accurate energy barriers, transition states geometries, and describe nonbonded and dispersive interactions, such as solvent effects and hydrogen bonds,^27^ which are significant to the systems under analysis. Furthermore, the M06–2X functional have been previously employed for the DFT investigation of epoxides ring-opening.^32^ The nature of the stationary points was characterized according to their second-order Hessian matrix: minimum energy points on the potential energy surface were identified as those with only positive eigenvalues, while first-order saddle points (transition state structures) with one and only negative eigenvalue.

Thermodynamic parameters were computed at 298 K and 1 atm, after frequency calculation in the default configuration using the standard statistical thermodynamics equations. In addition to the gas phase calculations, all the pathways were simulated with implicit solvation, employing the self-consisted reaction field (SCRF) option of Gaussian 09, implementing the integral equation formalism variant of the polarizable continuum model (IEFPCM)^33,34^ with 1,4-dioxane (ε = 2.20), acetonitrile (ε = 35.68) and water (ε = 78.35) as solvents. Moreover, explicit microsolvation using the supermolecule model was applied in every step of each pathway, simulating one water molecule in the specific sites which the reactions take place.

In order to obtain more reliable electronic energies and solvation contributions, single-point energy calculations were performed using the ωB97M-V functional^35,36^ with the def2-TZVP basis set,^28^ both in the gas phase and with the SMD solvation model,^37^ which is based on the molecular electron density. These calculations were carried out using the ORCA 6.0 program package,^38,39^ where ωB97M-V functional is implemented. This functional was selected for its well-documented accuracy in describing organic systems,^36^ especially those involving charged species,^40^ and its reliable prediction of intermolecular interactions such as hydrogen bonding^41^—features particularly relevant to the system under investigation. The SMD solvation model is widely used for simulating organic reactions in solution because it captures both electrostatic and nonelectrostatic solvation effects by combining a self-consistent reaction field approach with empirical terms for dispersion, cavitation, and solvent structure, making it an efficient tool for modeling reactivity in condensed-phase systems.^37,42,43^

Accordingly, the refined electronic energies computed at the ωB97M-V/def2-TZVP level, in both the gas phase and with the SMD solvation model, were employed in the construction of the free energy profiles. Based on these, the reaction and activation free energies in the gas phase *G*_g_ and in solution *G*_sol_ were computed according to following equations

1 $$G_{g}=E_{e\,\mathrm{lg}}+G_{\mathrm{vrtg}}$$

2 $$G_{\mathrm{sol}}=E_{\mathrm{el}\mathrm{sol}}+G_{\mathrm{vrt}\mathrm{sol}}+1.89\;\mathrm{kcal}\;{\mathrm{mol}}^{-1}$$

where *E*_elg_ and *E*_elsol_ refer to the electronic energies obtained at the ωB97M-V/def2-TZVP level in the gas phase and with the SMD solvation model, respectively. *G*_vrtg_ and *G*_vrtsol_ represent the vibrational, rotational, and translational contributions to the free energy (at 1 atm), computed at the M06–2X/def2-TZVP method in the gas phase and with the IEFPCM solvation model, respectively. To account for the change in standard-state concentration from 1 atm (gas phase) to 1 mol L^–1^ (solution), an entropy correction of 1.89 kcal mol^–1^ was added to the absolute free energies of all solvated species, as recommended in recent studies focused on the construction of solution-phase free energy profiles.^37,44–46^

Finally, natural population analysis (NPA) was carried out using the Natural Bond Orbital method (NBO6),^47^ at ωB97M-V/def2-TZVP level with SMD solvation model, using the ORCA 4.0 program package,^39,48^ to evaluate the natural atomic charges of the prereactive conformers of the epoxide ring-opening and intramolecular elimination steps (**PRC**_**1**_ and **PRC**_**2**_, respectively), previously fully optimized at the M06–2X/def2-TZVP level.

## Results and Discussion

Some authors have previously reported the conversion of *N*-Boc-epoxides into oxazolidinones.^16,49–51^ Usually, the reaction happens in the presence of peroxy acid catalysts, such as *meta*-chloroperoxybenzoic acid (*m*-CPBA) or mineral acids, after long reaction times. Therefore, we were puzzled that this transformation occurred in the presence of an amine (that is, a mildly basic environment), after a few hours of refluxing isopropanol—Scheme 2a. In an attempt to privilege the formation of the oxazolidinone as the primary product, we decided to reflux (2*S*,3*S*)-3-(Boc-amino)-1,2-epoxy-4-phenylbutane **1** in isopropanol without any catalysts, obtaining the desired (4*S*,5*R*)-4-benzyl-5-(hydroxymethyl)-1,3-oxazolidin-2-one **2** in 24 h, in excellent yields (96%), after washing with ethyl ether (Scheme 3).

**Scheme 3:**
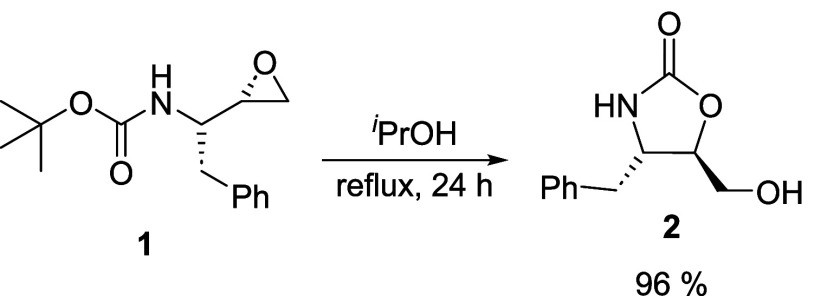
Formation of Oxazolidinone 2 in Refluxing *^i^* PrOH

To further investigate this reaction, we attempted to transform **1** in **2** by refluxing **1** in different solvents, ranging from polar protic (entries **1–5**, in Table 1) to aprotic (entries **6–9**, in Table 1), without the addition of any acidic or basic catalyst. The reactions were monitored by thin-layer chromatography (TLC) for up to 7 h, after which the yields were estimated by gas chromatography–mass spectrometry (GC-MS) analysis.

**Table 1:**
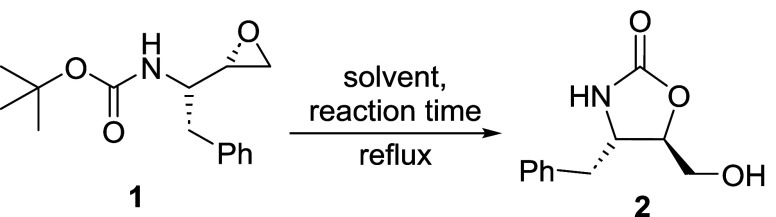
Estimated Yields Obtained in Various Polar Protic and Polar Aprotic Solvents after Reflux (Maximum of 7 h)

entry	solvent	reaction time (h)	estimated yield (GC-MS, %)
**1**	H_2_O	2	87
**2**	MeOH/H_2_O (1:1)	7	86
**3**	MeOH	7	67
**4**	EtOH	7	45
**5**	^*i*^PrOH	7	73
**6**	MeCN	7	0
**7**	THF	7	0
**8**	dioxane	7	0
**9**	AcOEt	7	0

After 7 h, the formation of **2** as the major product was observed in all polar protic solvents (entries **2–5**), with yields ranging from 45 to 86%. Interestingly, no product was detected in aprotic solvents (entries **6–9**). However, in most cases, 7 h were insufficient for the reaction to reach completion under these conditions, except for the mixture MeOH/H_2_O (1:1). Given these results, we then tested water as the sole solvent, despite the poor hydrosolubility of **1** (entry **1**). To our surprise, in addition to achieving comparable yields (87%), the reaction proceeded to completion significantly faster, requiring only 2 h under reflux, more than three times faster than in the MeOH/H_2_O (1:1) system. These findings prompted us to explore the reaction mechanism through quantum-chemical calculations, aiming to understand the transformation’s nature and the solvent’s influence on its course.

The proposed pathway for converting **1** into **2** passes through the epoxide ring-opening promoted by the *N*-Boc group.^14,15,25^ The ring-opening of epoxides traditionally occurs through two main pathways: with the aid of an acid catalyst or via a reaction with strong nucleophiles (base-catalyzed).^52,53^ In an acid-catalyzed opening, the reaction typically happens at the more substituted carbon, following a borderline nucleophilic substitution (an intermediate mechanism between S_N_1 and S_N_2).^52–55^ In the second case, the opening preferentially occurs at the less substituted carbon, following a classic S_N_2 mechanism.^52–54^ In this case, the formation of 2-oxazolidinones from *N*-Boc-epoxides is associated with the participation of the nitrogen-protecting group *tert*-butyl carbamate (Boc) of the substrate (named **PRC**_**1**_, which stands for prereactive conformer 1) in the intramolecular cyclization reaction (Scheme 4a).^14–16^

**Scheme 4:**
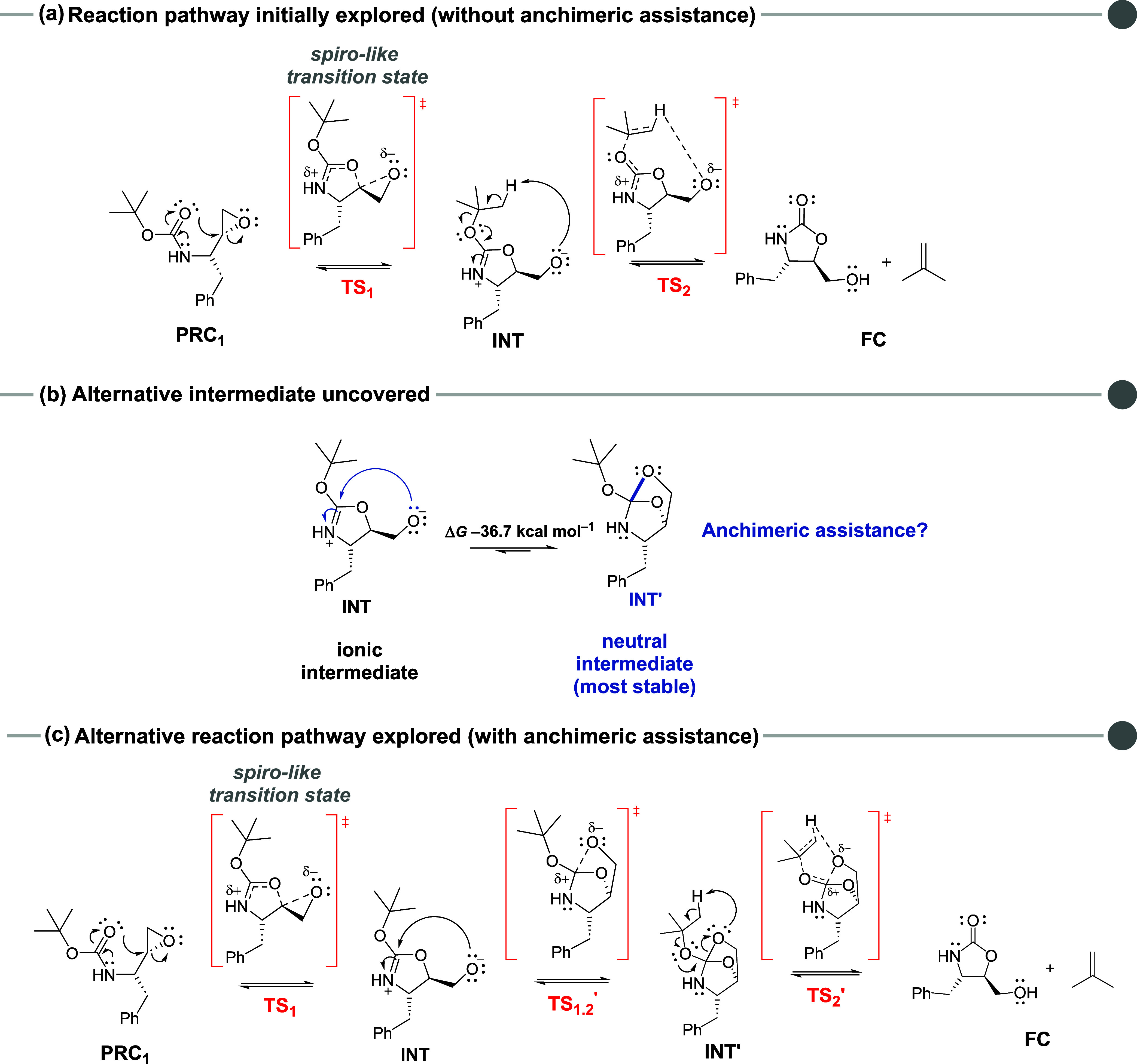
Possible Reaction Pathways Evaluated for the Formation of 2-Oxazolidinone 2: (a) Classical Mechanism Passing through a Zwitterion Intermediate; (b) Intermediates Identified as Minimum-Energy Points on the Gas-Phase Potential Energy Surface (Gibbs Free Energy Change Computed at ωB97M-V/def2-TZVP//M06-2X/def2-TZVP Level of Theory); (c) Proposed Mechanism Passing through a Neutral Bicyclic Intermediate

According to the previously proposed mechanism (Scheme 4a), the formation of the five-membered heterocycle **2** results from the underestimated reactivity of the *N*-Boc group, promoting the epoxide ring-opening via an spiro-like transition state (**TS**_**1**_).^14,18^ After the formation of the zwitterionic intermediate **INT**, the *t*-butyl fragment of the *N*-Boc group is eliminated, which may occur in a cationic form or, in certain cases, with the neighboring alkoxide group participation (**TS**_**2**_), generating isobutylene as a coproduct (**FC**, which stands for final complex).^16,56,57^

Considering this evidence, we began investigating the reaction pathway (Scheme 4a), specifically in the gas phase. This approach was chosen to analyze the potential energy surface in an environment free from solvation effects, which are known to stabilize transition states and ionic intermediates through both macroscopic and molecular-level properties, such as dielectric constant and intermolecular interactions, respectively.^58,59^ By doing so, we aimed to provide a comprehensive understanding of the system’s intrinsic reactivity, without the influence of bulk solvent properties or hydrogen-bond donor interactions, which might otherwise facilitate epoxide ring opening—similar to acid catalysis.^32,60^

Surprisingly, while searching for the relevant stationary points on the potential energy surface, the zwitterionic intermediate **INT** converged during optimization into a more stable structure—a neutral bicyclic intermediate (Scheme 4b), hereafter referred to as **INT**′. This transformation resulted in a significant stabilization of the reactive intermediate (|Δ*G*| = 36.7 kcal mol^–1^ in favor of **INT**′). The unprecedented formation of this neutral intermediate arises from the interaction between the electron-rich alkoxide, generated from the epoxide ring-opening in the first step of the reaction, and the electron-deficient carbon in the newly formed five-membered ring. From this observation, we began exploring the possibility of anchimeric assistance (and, thus, an uncovered reaction pathway, presented in the Scheme 4c)—a direct interaction between a reaction center and either a lone pair of electrons or the electrons in a nonconjugated σ- or π-bond within the parent molecule—that, as defined by the IUPAC Gold Book, can enhance the reaction rate.^61^

Since we experimentally observed an inversion of configuration from the *N*-Boc-epoxide substrate **1** to the resulting product **2**, the zwitterionic intermediate **INT** must be formed prior to the bicyclic intermediate **INT**′, via a classical backside nucleophilic attack (**TS**_**1**_),^62^ which accounts for the observed experimental outcome (Figure 2a). Attempts to locate a transition state connecting the substrate **PRC**_**1**_ directly to **INT**′ led to a frontside nucleophilic attack of the Boc group on the epoxide moiety (Figure 2b). However, since frontside nucleophilic substitutions typically involve higher activation barriers than backside attacks—ascribed mainly to steric repulsion between the nucleophile and the adjacent leaving group in the transition state—and are stereospecific, resulting in retention of configuration,^63,64^ this transition state could not be connected to our substrate (**PRC**_**1**_) on the potential energy surface.

**Figure 2:**
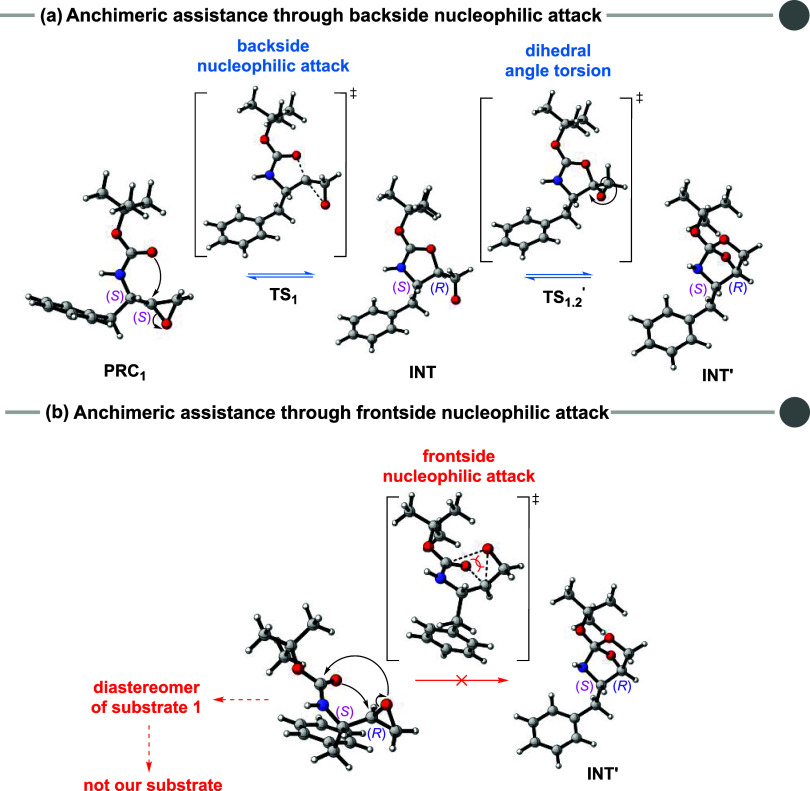
Possible anchimeric assistance pathways evaluated. (a) Formation of **INT**′ via backside nucleophilic attack; (b) Formation of **INT**′ via frontside nucleophilic attack. Optimized molecular structures obtained at M06–2X/def2-TZVP method in the gas phase.

Therefore, to rationalize this potentially lower-energy pathway (Scheme 4c), we proposed a new transition state **TS_1.2_′** after epoxide ring-opening **TS**_**1**_, connecting the zwitterionic intermediate to the bicyclic intermediate, and a two-step “concerted” transition state **TS**_**2**_ **′**, connecting the bicyclic intermediate to the final complex **FC** (2-oxazolidinone and isobutylene). “Concerted” here means that the elimination of isobutylene occurs as the intramolecular assistance that stabilizes **INT**′ is disrupted in **TS**_**2**_ **′** to form the desired oxazolidinone **2**. The potential energy surfaces for these reaction pathways and the nature of all transition states were evaluated by IRC calculations (see the Supporting Information, SI).

To incorporate solvation effects, we began by simulating 1,4-dioxane—the solvent with the lowest dielectric constant (ε = 2.20) among those experimentally assessed. Figure 3 presents the Gibbs free energy profile along the reaction coordinate, comprising the epoxide ring-opening followed by intramolecular elimination, in the presence of 1,4-dioxane implicitly simulated. Overall, and in good agreement with the experimental findings, the reaction appears unfeasible in a medium lacking strong stabilizing interactions, as evidenced by the optimal reaction condition results (Table 1). In this context, low-polarity aprotic solvents such as ethyl acetate, 1,4-dioxane, and THF (entries **7–9**) failed to yield product **2**. This is consistent with the elevated activation free energy observed, all exceeding 40 kcal mol^–1^. Although 1,4-dioxane showed some ability to stabilize the reaction relative to the gas-phase energy profile (see Section S3 in the Supporting Information), the results indicate that, under these conditions, the product formation are limited due to kinetic reasons. Therefore, for an epoxide ring-opening reaction conducted in the absence of an acid catalyst and in aprotic solvents, a high-energy profile is to be expected.^53^

**Figure 3:**
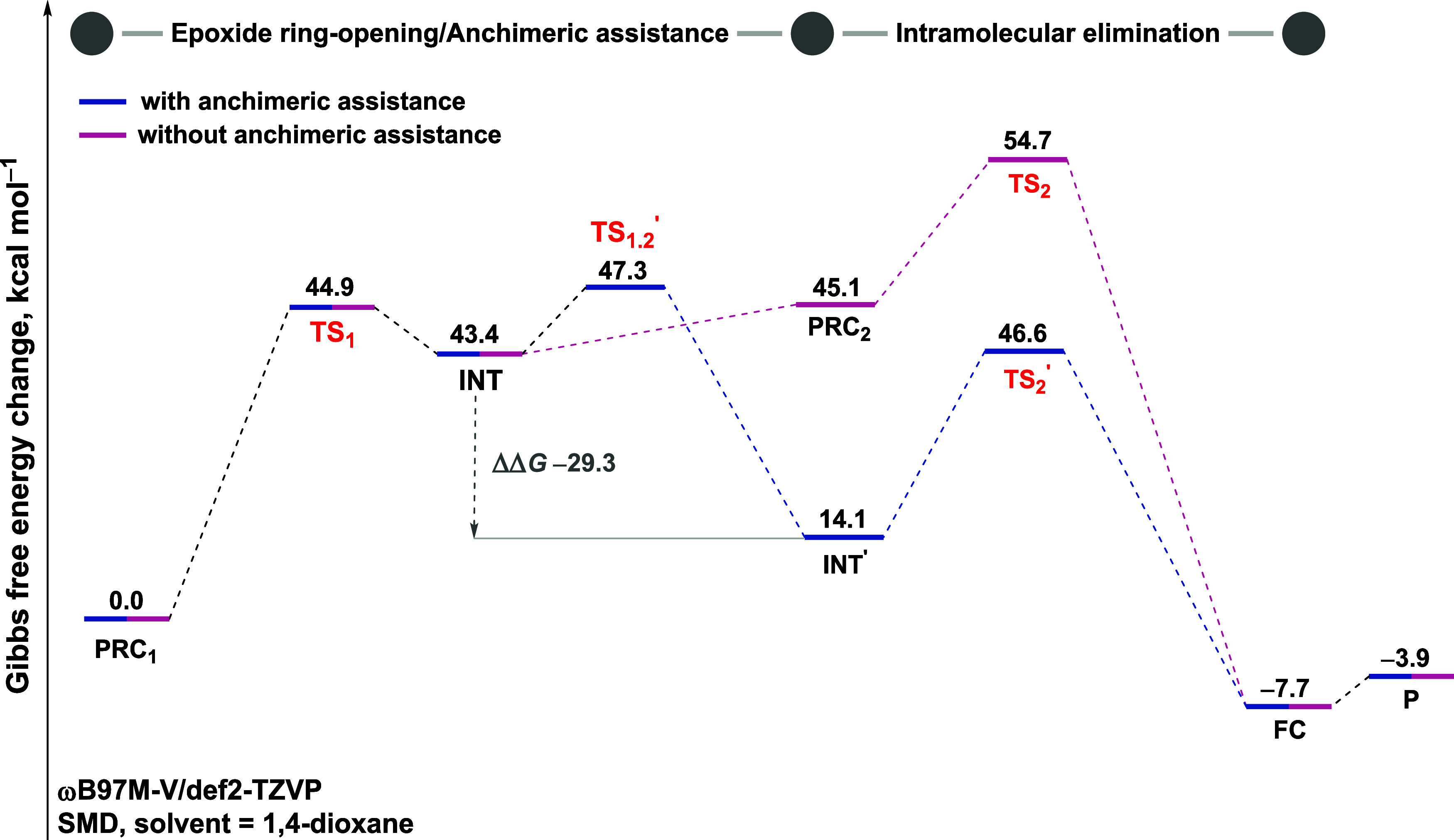
Gibbs free energy change (in kcal mol^–1^) at 25 °C and 1 mol L^–1^ standard state for reaction pathways with anchimeric assistance (in blue) and without anchimeric assistance (in purple). The transition states are highlighted in red. “P” is referred to as the products free species (isobutylene and the 2-oxazolidinone **2**). Values computed at the ωB97M-V/def2-TZVP//M06–2X/def2-TZVP level of theory. Solvent effect calculated by the SMD model (1,4-dioxane).

Once the zwitterionic intermediate **INT** is formed, it can rapidly convert into the bicyclic intermediate **INT′** via the transition state **TS**_**1.2**_ **′**, characterized by the torsion of a dihedral angle in the alkoxide moiety. This transformation proceeds with a low energy barrier (Δ*G* ^‡^ **=** 3.9 kcal mol^–1^ relative to **INT**), attributed to the proximity of a negatively charged alkoxide ion to an electrophilic center (the electron-deficient carbon in the newly formed five-membered ring).

The intramolecular elimination from the intermediates can proceed via two distinct pathways. Passing by **TS**_**2**_ (purple), the negatively charged oxygen atom generated during epoxide ring-opening deprotonates the *tert*-butyl group of the Boc moiety, leading to isobutylene release. Along this route, **INT** converts to **PRC**_**2**_ (prereactive conformer 2), which precedes **TS**_**2**_. Alternatively, in the anchimeric assistance pathway (**TS**_**2**_ **′**, blue), isobutylene is released as the neutral bicyclic intermediate opens to form the final product. **TS**_**2**_ is more energetic than **TS**_**2**_ **′** by 8.1 kcal mol^–1^, likely due to the significantly greater stability of **INT’** compared to **INT**. Hence, from the formation of the neutral intermediate (**INT′**) onward, the pathway with anchimeric assistance becomes energetically favored.

A significant and progressive stabilization of the transition states and charge-separated intermediates is observed with increasing solvent polarity, modeled using implicit solvation models (acetonitrile, ε = 35.68; and water, ε = 78.35; Figure 4—purple and blue curves, respectively). In addition, explicit microsolvation with water further stabilizes the entire energy profile (Figure 4, in orange). While implicit models simulate bulk solvent effects by accounting for charge stabilization via high dielectric constants, the explicit model also incorporates specific intermolecular interactions, such as hydrogen bonding, which promote charge delocalization and facilitate proton transfer along the reaction pathway. In this case, we simulated one water molecule in all the structures, strategically positioned to form hydrogen bond with the oxygen atom of the epoxide ring—the reactive site where the negative charge is developed. This approach aligns with the work of Jamison and co-workers on elucidating the role of water in the epoxide-opening cascades involved in the biogenesis of ladder polyether natural products. Their studies suggest that water can act as a reaction promoter in epoxide ring openings by activating the oxirane ring through hydrogen bonding.^24,65,66^

**Figure 4:**
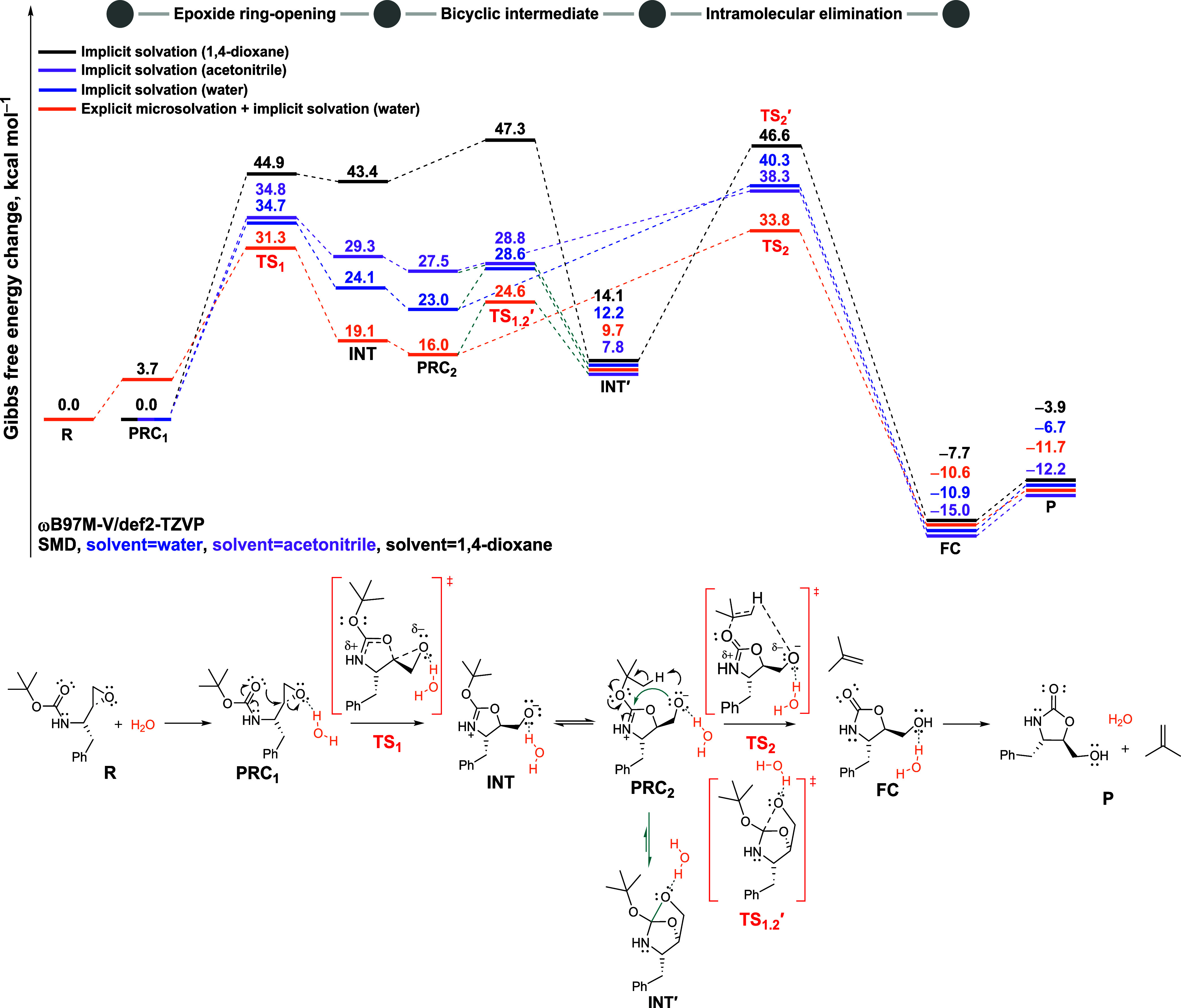
Gibbs free energy change (in kcal mol^–1^) at 25 °C and 1 mol L^–1^ standard state for the reaction pathways with implicit solvation in 1,4-dioxane (black), acetonitrile (purple), and water (blue), as well as explicit microsolvation combined with implicit solvation in water (orange). Transition states are highlighted in red. “R” and “P” refer to the free reactants and products species, respectively. All values were computed at the ωB97M-V/def2-TZVP//M06–2X/def2-TZVP level of theory. Solvent effect calculated by the SMD model.

Figure 4 presents the Gibbs free energy change for the reaction in different solvent environments. Considering the anchimeric assistance possibility, we can now divide the reaction pathway into three stages: epoxide ring-opening, formation of the bicyclic intermediate, and the intramolecular elimination step.

Generally, under implicit solvation with acetonitrile (purple) and water (blue), the bulk effects of these polar solvents—particularly their high dielectric constants (ε = 35.68 and ε = 78.35, respectively)—stabilize the charged intermediate **INT**, resulting in a substantial decrease in the energy of the associated transition states. However, in this continuum model, the lower-energy concerted transition state **TS_2_′**, which connects **INT′** directly to the product complex, could not be located—likely due to the stabilization of the alkoxide ion by the polar medium. This suggests that, in the continuum medium, the formation of the bicyclic intermediate (**INT′**) could be considered as an anchimeric assistance.

Still, the Gibbs free energy barrier to form this more stable intermediate **INT′** via **TS**_**1.2**_ **′** (Δ*G* ^‡^ = 28.8 kcal mol^–1^ in acetonitrile and 28.6 kcal mol^–1^ in water, both relative to their **PRC**_**1**_) is lower than that of the elimination step via **TS**_**2**_ (Δ*G* ^‡^ = 38.3 kcal mol^–1^ in acetonitrile and Δ*G* ^‡^ = 40.3 kcal mol^–1^ in water relative to **PRC**_**1**_). However, once the **TS**_**2**_ energy barrier is overcome, the reaction becomes irreversible, with the products formation being highly exergonic (Δ*G* = −15.0 kcal mol^–1^ in acetonitrile and Δ*G* = −10.9 kcal mol^–1^ in water relative to **PRC**_**1**_). These results suggest that, prior to the product formation, the system may interconvert both intermediates under equilibrium—albeit with **INT** present at low concentration, as it is less stable than **INT′** by 21.5 kcal mol^–1^ in acetonitrile and 11.9 kcal mol^–1^ in water.

Although acetonitrile and water implicitly simulated stabilize key transition states, the energy profile remains kinetically limited. The highest energy point along the pathway (**TS**_**2**_, in the intramolecular elimination step) is approximately 40 kcal mol^–1^ in both solvents. This finding indicates that additional factors, such as explicit hydrogen bonding, is likely required for the reaction to proceed efficiently.

Upon implementing the explicit microsolvation model (Figure 4, orange), we observed additional stabilization along the entire reaction pathway. In this model, the reference point for the free energy profile is defined as **R** (reactants), corresponding to the free species composed of the substrate and one water molecule. Under these conditions, the formation of the prereactive complex **PRC**_**1**_, in which the water molecule forms a hydrogen bond with the epoxide moiety, is associated with a free energy of Δ*G* = +3.7 kcal mol^–1^, consistent with the poor hydrosolubility of the substrate. Nevertheless, water plays a crucial role along the reaction course by facilitating the formation of oxazolidinone **2**. Specifically, when excluding the free energy cost associated with the formation of the substrate-water adduct, the hydrogen bond formed between the added water molecule and the oxygen atom of the electrophilic epoxide—combined with implicit water solvation—further lowers the activation free energy for **TS**_**1**_, from Δ*G* ^‡^ = 34.7 kcal mol^–1^ (in blue) to 27.6 kcal mol^–1^ (in orange), relative to **PRC**_**1**_, as shown in Figure 4. Moreover, the charge-separated zwitterionic intermediate **INT** also undergoes substantial stabilization. The intramolecular elimination transition state (**TS**_**2**_) benefits from significant stabilization as well, with its activation free energy decreasing from Δ*G* ^‡^ = 40.3 kcal mol^–1^ (in blue) to Δ*G* ^‡^ = 30.1 kcal mol^–1^ relative to **PRC**_**1**_, it remains the highest-energy structure along the reaction pathway.

Table 2 compares the energy barriers for the epoxide ring-opening (first stage) with the intramolecular elimination (final stage) by subtracting the energies of the transition state from its respective prereactive conformer (i.e., **TS**_**1**_ **–PRC**_**1**_ and **TS**_**2**_ **–PRC**_**2**_, respectively). When the explicit solvation model is applied, we observe opposing trends between these two reaction stages. For the epoxide ring-opening step, the activation free energy is reduced by 7.1 kcal mol^–1^. In contrast, for the intramolecular elimination step, the barrier slightly increases (ΔΔ*G* ^‡^ = 0.5 kcal mol^–1^).

**Table 2:**
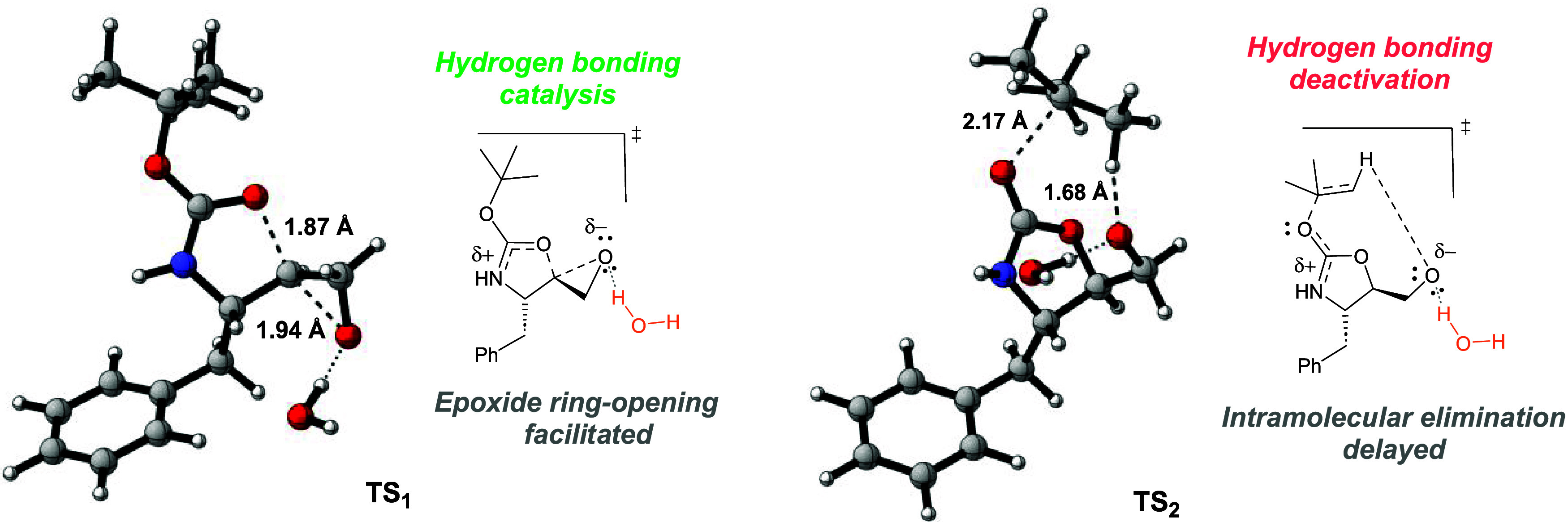
Effect of Water’s Hydrogen-Bond Donation on the Activation Free Energy (in kcal mol^–1^, 25 °C and 1 mol L^–1^ Standard State) of the Transition State Structures TS_1_ and TS_2_ Calculated at ωB97M-V/def2-TZVP//M06-2X/def2-TZVP-SMD (Water) Using Implicit Solvation and Explicit Microsolvation Models

		epoxide ring-opening (first stage)	intramolecular elimination (final stage)
solvation model	energy barrier (kcal mol^–1^)	**TS**_**1**_–**PRC**_**1**_	**TS**_**2**_–**PRC**_**2**_
implicit	Δ*G* ^‡^	34.7	17.3
implicit + explicit (microsolvation)	Δ*G* ^‡^	27.6	17.8

To confirm the reliability of these trends, we evaluated the main activation free energy (**TS**_**1**_ and **TS**_**2**_) using additional density functionals: M06–2X, ωB97X-D, CAM-B3LYP and B3LYP-D3 (see Section S5 in Supporting Information). The results show consistent activation free energies across all tested functionals, supporting the robustness of our computational findings. Notably, these functionals are widely recommended for calculating thermochemical data and reaction barriers in organic systems,^67^ particularly when accurate descriptions of nonbonded and dispersive interactions—such as hydrogen bonding and conjugated systems—are required.^27,68^ Regardless of the functional employed, implementation of the implicit solvation model combined with the microsolvation consistently resulted in lowered activation barrier for the epoxide ring-opening step, while increasing the barrier for the intramolecular elimination step.

Over the years, epoxide ring-opening reactions assisted by polar protic solvents—often described as solvent-catalyzed processes—have been reported to proceed via hydrogen bonding catalysis.^22,23,69,32^ Williams and co-workers have computationally elucidated the hydrogen-bond-assisted activation in spirodiepoxide-opening reactions, demonstrating that transition state stabilization facilitates the process.^70^ More recently, Ahsan and co-workers conducted a computational investigation into the electronic basis of water’s catalytic effect, particularly in epoxide ring-opening promoted by aspartate proteases.^32^ Their results align well with our findings, as shown in Table 2.

In our study, water accelerates the epoxide ring-opening by stabilizing the transition state through hydrogen bonding, reducing the activation free energy (ΔΔ*G* ^‡^ = 7.1 kcal mol^–1^ for **TS**_**1**_) compared to only implicit solvation model. However, in the intramolecular elimination step, water appears to hinder the proton abstraction from the *tert*-butyl fragment, likely due to a decrease in the intrinsic basicity of the alkoxide ion caused by hydrogen-bonding deactivation. These observations emphasize the catalytic potential of water and other polar protic solvents as valuable tools for optimizing organic reactions under mild conditions.

As a complementary evaluation, Natural Bond Orbital (NBO) analysis, a well-established method for describing various chemical bonding concepts—including bond order, delocalization, orbital occupancy, and atomic charges—was conducted to assess the atomic charges of the system along the reaction coordinate.^71–73^ This approach provides valuable insights into electronic distribution within the molecule.^73^ The calculated natural atomic charges of the prereactive conformers of the epoxide ring-opening and intramolecular elimination steps (**PRC**_**1**_ and **PRC**_**2**_, respectively) are summarized in Figure 5. These findings support our data by identifying the carbonyl oxygen (O5) of the Boc group as the nucleophilic site of **PRC**_**1**_ (Figure 5a), characterized by substantial negative charge density across all evaluated media. These results further highlight the underestimated reactivity of the *N*-Boc protecting group. Meanwhile, the most substituted carbon atom C4 of the epoxide moiety emerges as the electrophilic site, as indicated by its slight positive charge density in **PRC**_**1**_ (Figure 5a). Furthermore, the electron deficiency of C1 remains evident even before cyclization (Figure 5a) and slightly increases in the zwitterionic structure **PRC**_**2**_ (Figure 5b), identifying it as a key reaction center. This characteristic facilitates its interaction with the negatively charged alkoxide (O14) in **PRC**_**2**_, ultimately leading to the formation of the most stable bicyclic intermediate (**INT′**).

**Figure 5:**
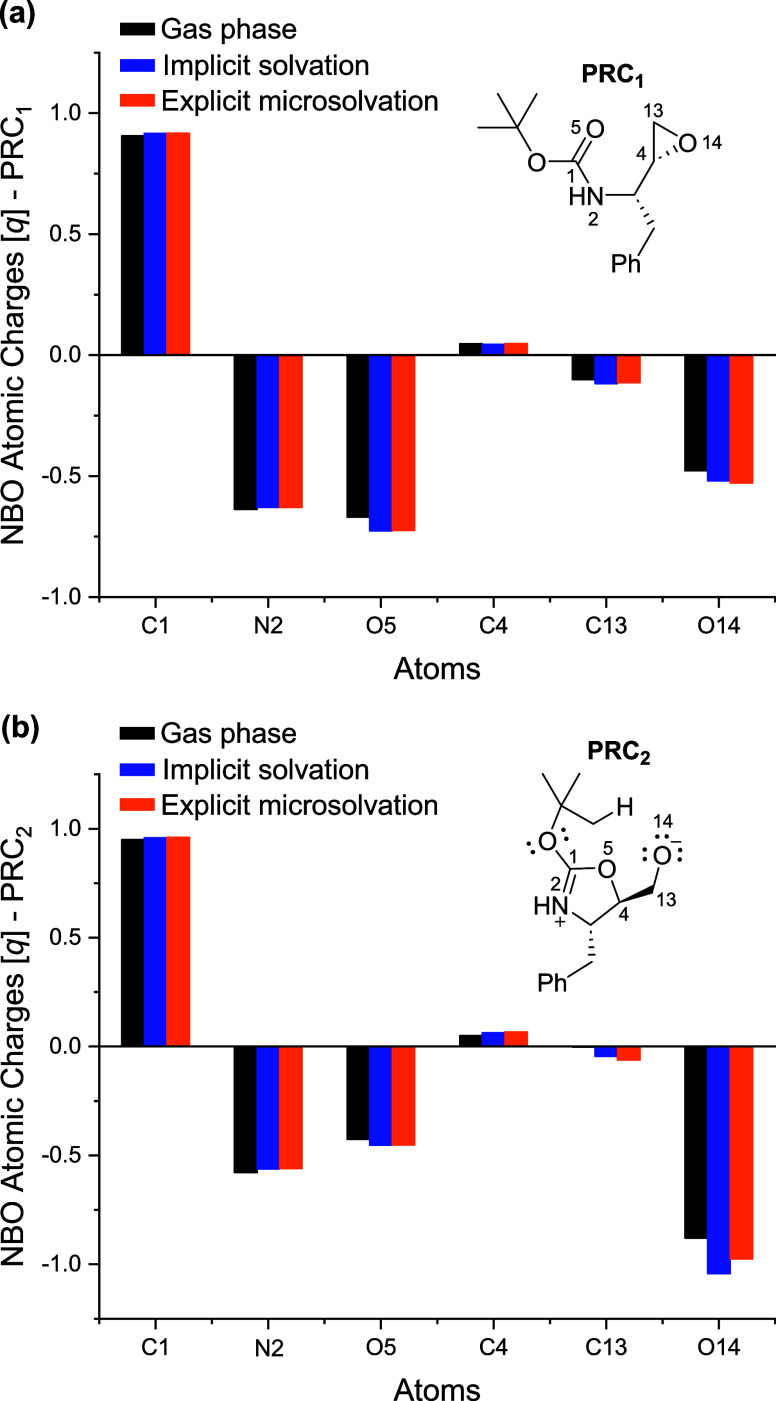
Natural atomic charges from the natural population analysis (NPA) for the reactive sites of the prereactive conformers of the epoxide ring-opening and intramolecular elimination steps (a) **PRC**_**1**_; and (b) **PRC**_**2**_, respectively) in the gas phase (black), implicit solvation with water (blue) and explicit microsolvation combined with implicit solvation in water (orange). Values computed at ωB97M-V/def2-TZVP//M06–2X/def2-TZVP (SMD water) from NBO method.

Finally, as discussed earlier, water slightly enhances the buildup of positive charge on the C4 epoxide carbon through hydrogen-bond catalysis, thereby facilitating the epoxide ring-opening, as evidenced by the increased positive charge density on C4 in the explicit microsolvation model (Figure 5a). Additionally, water reduces the basicity of the alkoxide (O14) in **PRC**_**2**_ compared to when only its bulk polar effects are simulated via implicit solvation (Figure 5b). These observations help to rationalize why the barrier for the intramolecular elimination increases, while the epoxide ring-opening barrier is reduced.

## Conclusions

Herein, we optimized the reaction conditions for the conversion of a phenylalanine-derived *N*-Boc-epoxide into a 2-oxazolidinone in polar protic solvents, particularly water, under acid-free conditions. Furthermore, based on DFT calculations, we identified a remarkably stable, bicyclic neutral intermediate in equilibrium with a zwitterionic species. The discovery of this unprecedented intermediate prompted us the investigation of an alternative mechanism involving anchimeric assistance.

Our computational exploration of the mechanism under four distinct conditions—implicit solvation with 1,4-dioxane, acetonitrile, and water, as well as explicit microsolvation with water—revealed substantial energetic differences among them. Notably, only in the presence of an explicit water molecule did the energy profile align with the observed experimental reactivity. This underscores that hydrogen-bonding catalysis, rather than general solvent polarity, is critical for efficient epoxide ring-opening. This finding is consistent with experimental observations showing no conversion in polar aprotic solvents, whereas successful formation of 2-oxazolidinone occurs in protic solvents such as methanol, isopropanol, and water.

## Supplementary Material



## Data Availability

The data underlying this study are available in the published article and its Supporting Information.

## Notes

Details of the complete experimental procedure, DFT calculation details, characterization data, nuclear magnetic resonance spectra, and high-resolution mass spectrometry (PDF)
