# Supplementary material for: Solvent-Assisted Catalysis: Water Promotes Ring Closure Formation of 2‑Oxazolidinone from Amino Acid Derived N‑Boc-Epoxide
Source: ACS Org Inorg Au. 2025 Jul 17;5(5):360–72. doi: 10.1021/acsorginorgau.5c00051 (PMC12492048; doi:10.1021/acsorginorgau.5c00051)
Supplement: Supplementary file 1 [file gg5c00051_si_001.pdf]

Solvent-Assisted Catalysis: Water Promotes Ring Closure Formation of 2-Oxazolidinone from Amino Acid Derived *N*-Boc-Epoxide

Patrick de L. Barbosa,[1] Victor Facchinetti,[2] Claudia Regina B. Gomes,[2] Marcus Vinícius de Souza,[2] Thatyana R. A. Vasconcelos,[1,*] Rodolfo G. Fiorot.[1,*]

[1]Department of Organic Chemistry, Institute of Chemistry, Universidade Federal Fluminense – UFF, Niteroi, Rio de Janeiro, 24020-141, Brazil.

[2]Department of Drug and Bioactive Synthesis, Institute of Drug Technology (Farmanguinhos), Fundação Oswaldo Cruz – Fiocruz, Rio de Janeiro, Rio de Janeiro, 21041-250, Brazil.

*Corresponding authors: rodolfofiorot@id.uff.br; thatyanavasconcelos@id.uff.br

Table of Contents

S1 General procedure for the synthesis of 2 in various solvents 2

S2 NMR spectral data, high-resolution mass spectral (HRMS) and IR spectrum of the compound 2 3

S3 Gas phase free energy profile 5

S4 Intrinsic Reaction Coordinate (IRC) calculations 6

S4.1 Gas phase calculations 6

S4.2 Implicit solvation calculations 8

S4.3 Explicit microsolvation calculations 12

S5 Variation of energy barriers for different functionals 13

S6 Optimized cartesian coordinate matrices 14

S6.1 Gas phase calculations 14

S6.2 Implicit solvation calculations 27

S6.3 Explicit microsolvation calculations 66

S1 General procedure for the synthesis of 2 in various solvents

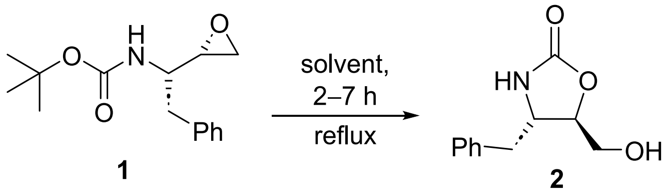

(2*S*,3*S*)-3-(Boc-amino)-1,2-epoxy-4-phenylbutane **1** (263 mg, 1mmol), and 5mL of the corresponding solvent (D_2O, H_2O, H_2O/MeOH, MeOH, EtOH, iPrOH, THF, dioxane, MeCN or ethyl acetate) were added to a round bottle flask under magnetic stirring under reflux in an oil bath for 7h or until completion of the reaction (TLC). Crude reactions were analyzed by GC-MS to estimate the oxazolidinone formation and yields. In the case of H_2O/MeOH and H_2O systems, reactions were purified by removing the solvents under reduced pressure, followed by trituration of the crude product with ethyl ether to afford (4*S*,5*R)*-4-benzyl-5-(hydroxymethyl)-1,3-oxazolidin-2-one.

(4*S*,5*R)*-4-benzyl-5-(hydroxymethyl)-1,3-oxazolidin-2-one (2):

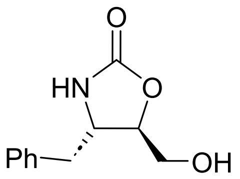

White Solid. Yield (H_2O): 87% (180.10 mg); **M.P.**: 115-117ºC. **HRMS (ESI-TOF):** *m/z* $[M+Na]^+$ calcd for $C_{11}H_{13}NO_3Na$ 230.0793, found 230.0792; **IR** (**KBr, cm^{-1}):** 3304 (OH); 1734 (C=O) **^{1}H NMR (DMSO-*d6*, 400 MHz):** δ 7.71 (s, 1H), 7.34-7.20 (m, 5H), 5.01 (s, 1H), 4.15 (q, 1H, *J* = 4.7 Hz), 3.81 (q, 1H, *J* = 5.8 Hz), 3.38 - 3.34 (m, 1H), 3.21 (dd, 1H, *J* = 12.1, 4.7 Hz), 2.83 (dd, 1H, *J* = 13.5, 5.6 Hz), 2.76 (dd, 1H, *J* = 13.5, 6.9 Hz). **^{13}C{^{1}H} NMR (CD_3OD, 125 MHz)**: δ 161.5; 137.7, 130.7, 129.9, 128.1; 83.3; 63.5; 56.4; 42.3.

S2 NMR spectral data, high-resolution mass spectral (HRMS) and IR spectrum of the compound 2

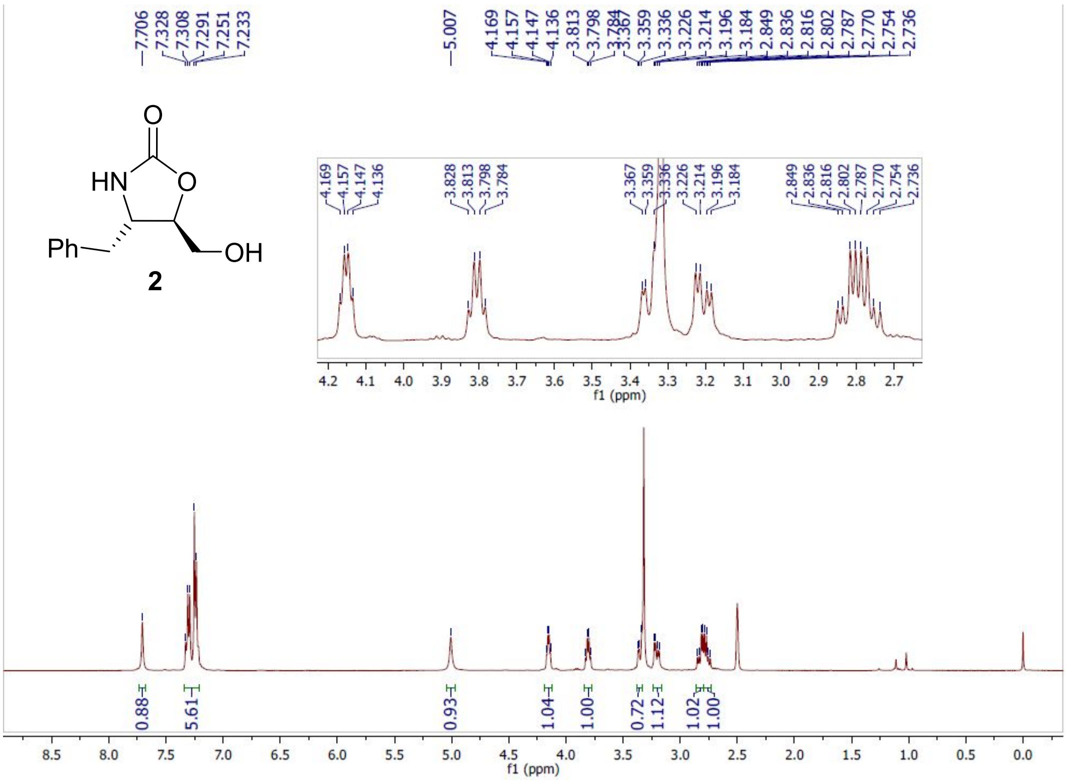

Figure S1: ^{1}H NMR spectrum (DMSO-d6, 400 MHz) of Oxazolidinone **2**.

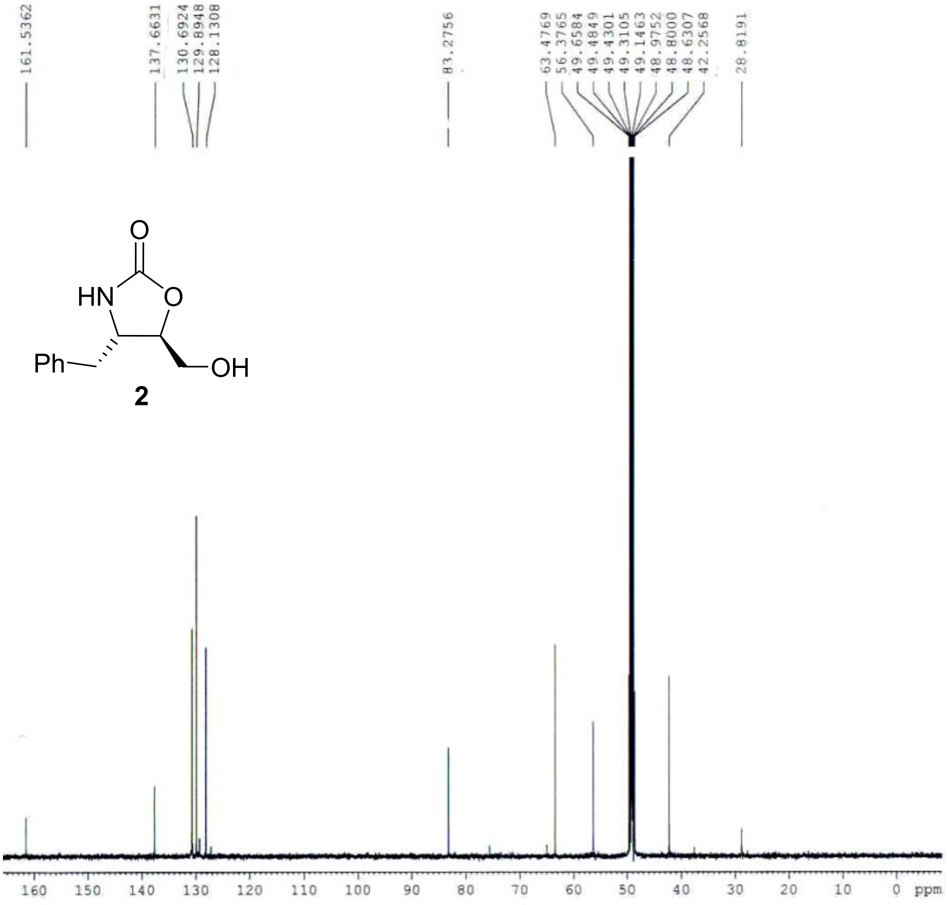

Figure S2. ^{13}C NMR spectrum (MeOD, 125 MHz) of Oxazolidinone **2**.

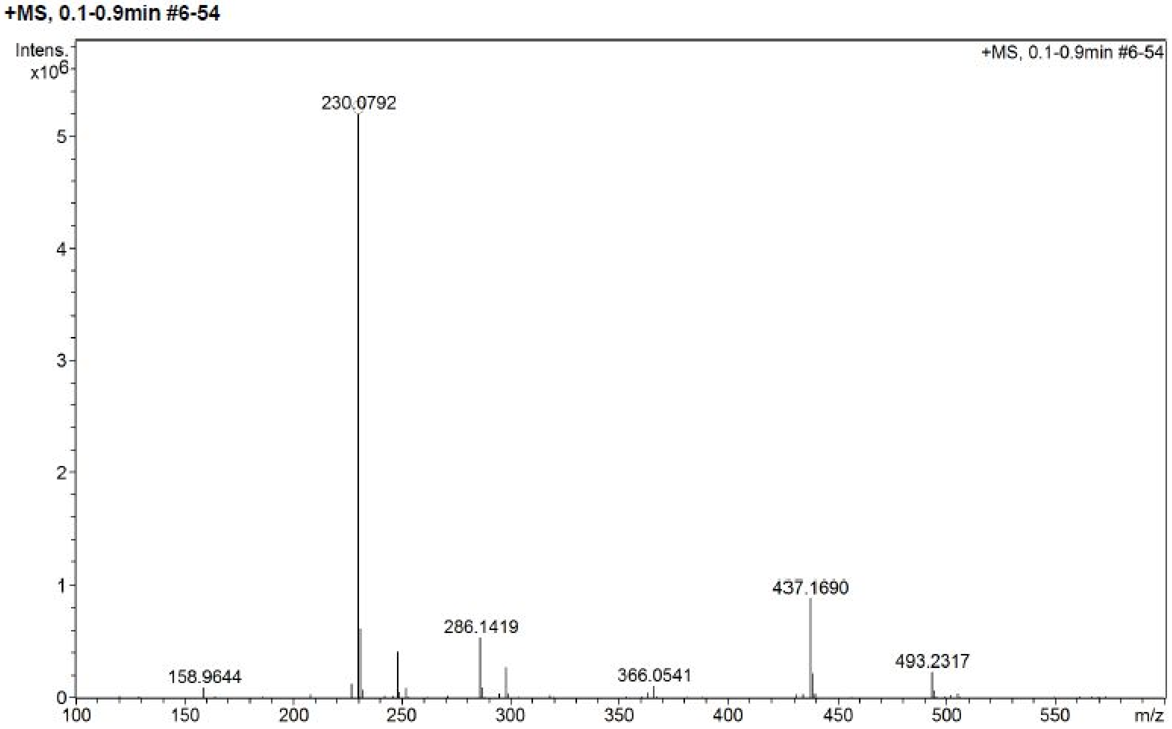

Figure S3. HRMS spectrum of oxazolidinone **2**.

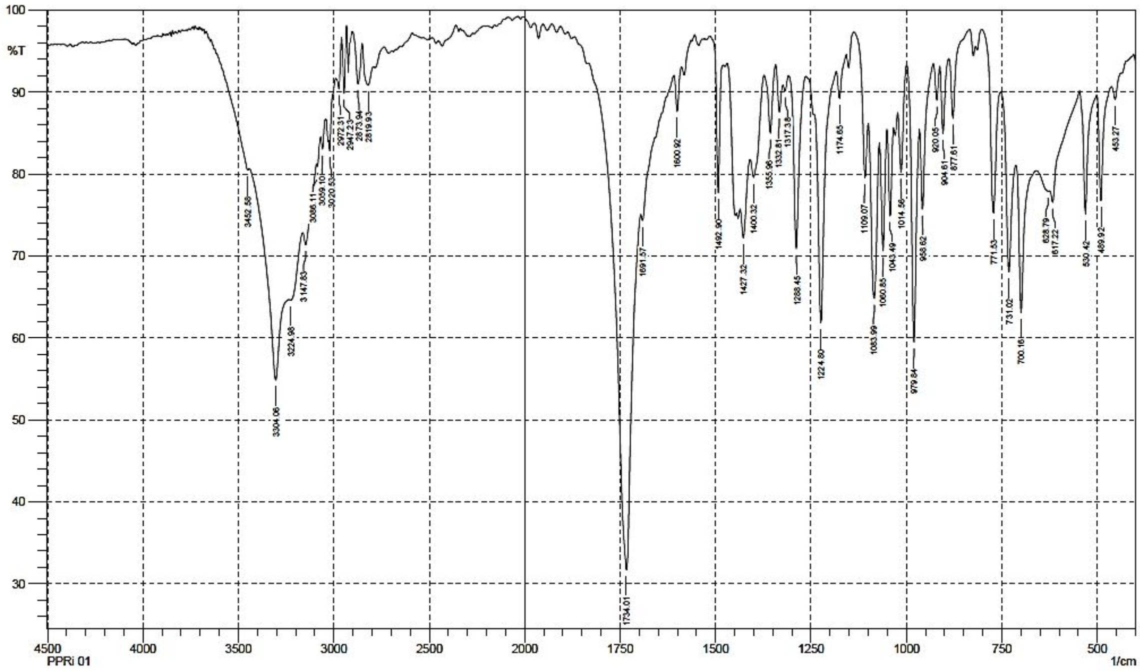

Figure S4. IR spectrum of oxazolidinone **2**.

S3 Gas phase free energy profile

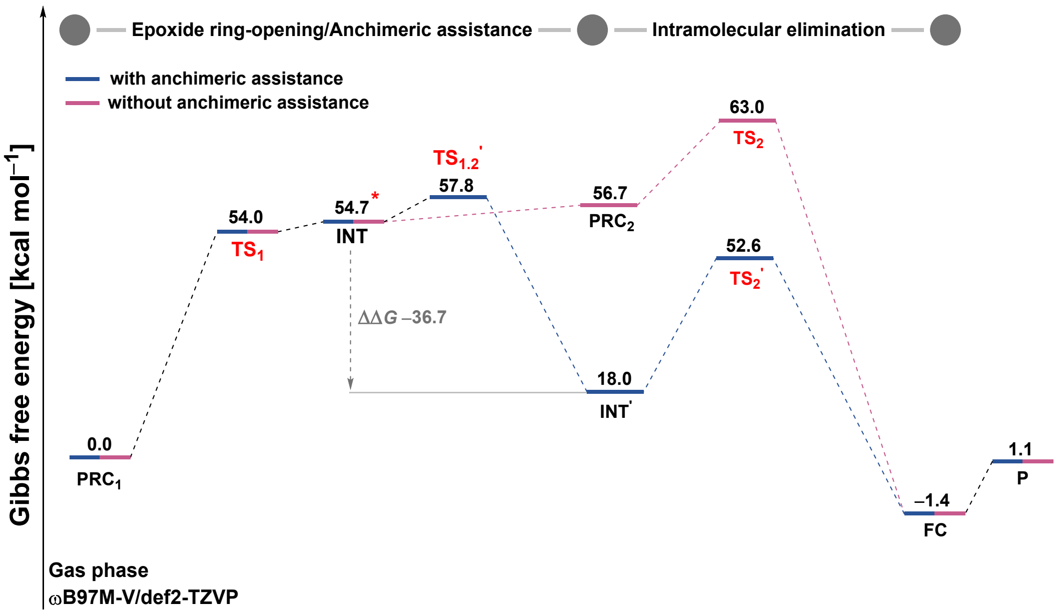

Figure S5: Gibbs free energy change (in kcal mol^{-1}) for reaction pathways with anchimeric assistance (in blue) and without anchimeric assistance (in purple). The transition states are highlighted in red. 'P' is referred to as the products free species (isobutylene and oxazolidin-2-one **2**). Values computed at the ωB97M-V/def2-TZVP//M06-2X/def2-TZVP in the gas phase.

*The apparently higher Gibbs free energy of **INT** relative to **TS$_1$** likely arises from intrinsic limitations of the computational method, which may introduce inaccuracies in the free energy correction to the electronic energy.

S4 Intrinsic Reaction Coordinate (IRC) calculations

Intrinsic reaction coordinate (IRC) calculations were carried out to confirm that each transition state structure was connected to its corresponding minimum energy points in the potential energy surface.

S4.1 Gas phase calculations

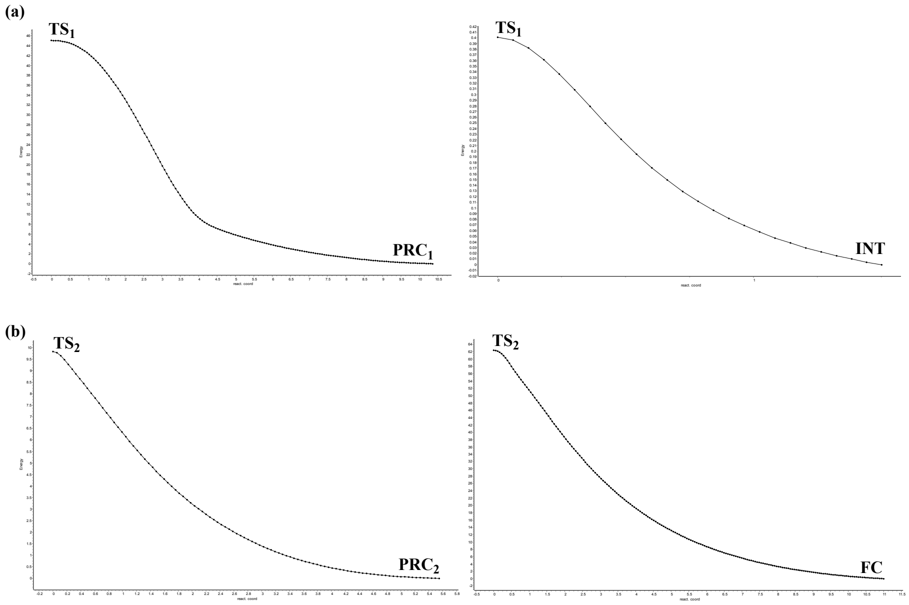

Figure S6. Reaction pathway without anchimeric assistance. **a)** Intrinsic reaction coordinate (IRC) calculated from **TS$_1$** structure (M06-2X/def2-TZVP) in the gas phase; **b)** Intrinsic reaction coordinate (IRC) calculated from **TS$_2$** structure (M06-2X/def2-TZVP) in the gas phase.

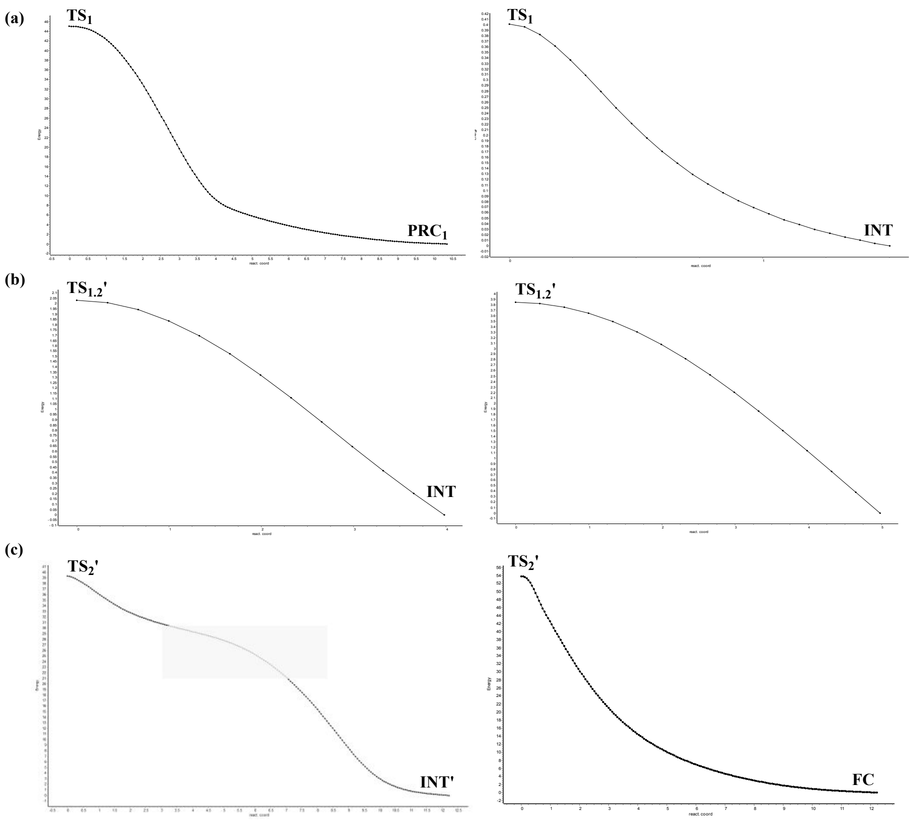

Figure S7. Reaction pathway with anchimeric assistance. **a)** Intrinsic reaction coordinate (IRC) calculated from $\mathbf{TS_1}$ structure (M06-2X/def2-TZVP) in the gas phase; **b)** Intrinsic reaction coordinate (IRC) calculated from $\mathbf{TS_{1.2}'}$ structure (M06-2X/def2-TZVP) in the gas phase; **c)** Intrinsic reaction coordinate (IRC) calculated from $\mathbf{TS_2'}$ structure (M06-2X/def2-TZVP) in the gas phase.

S4.2 Implicit solvation calculations

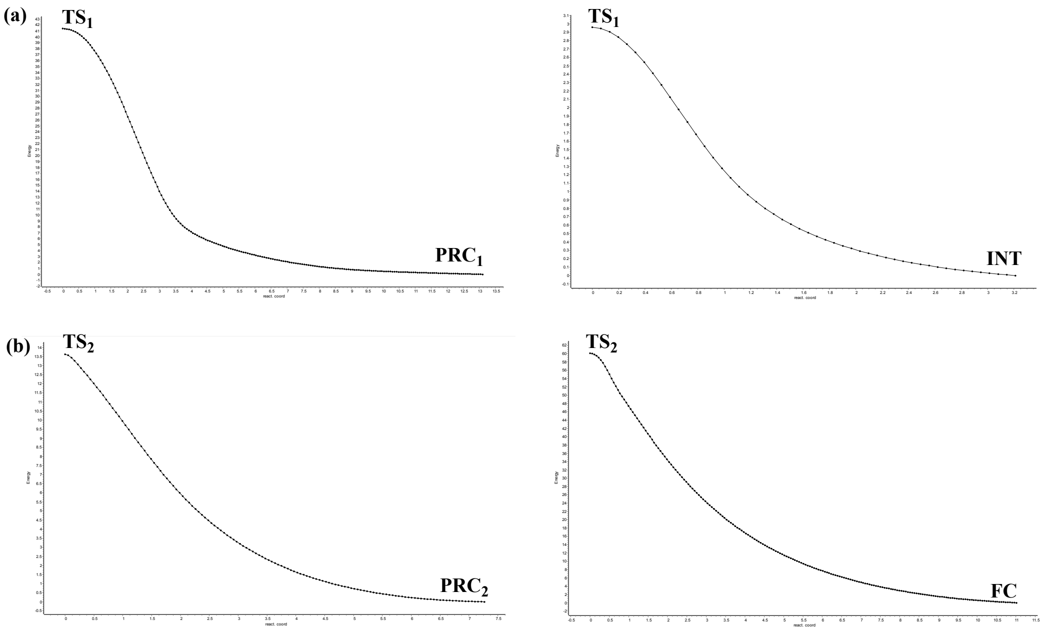

Figure S8. Reaction pathway without anchimeric assistance. **a)** Intrinsic reaction coordinate (IRC) calculated from $\mathbf{TS_1}$ structure M06-2X/def2-TZVP/IEFPCM (1,4-dioxane) in implicit solvation; **b)** Intrinsic reaction coordinate (IRC) calculated from $\mathbf{TS_2}$ structure M06-2X/def2-TZVP/IEFPCM (1,4-dioxane) in implicit solvation.

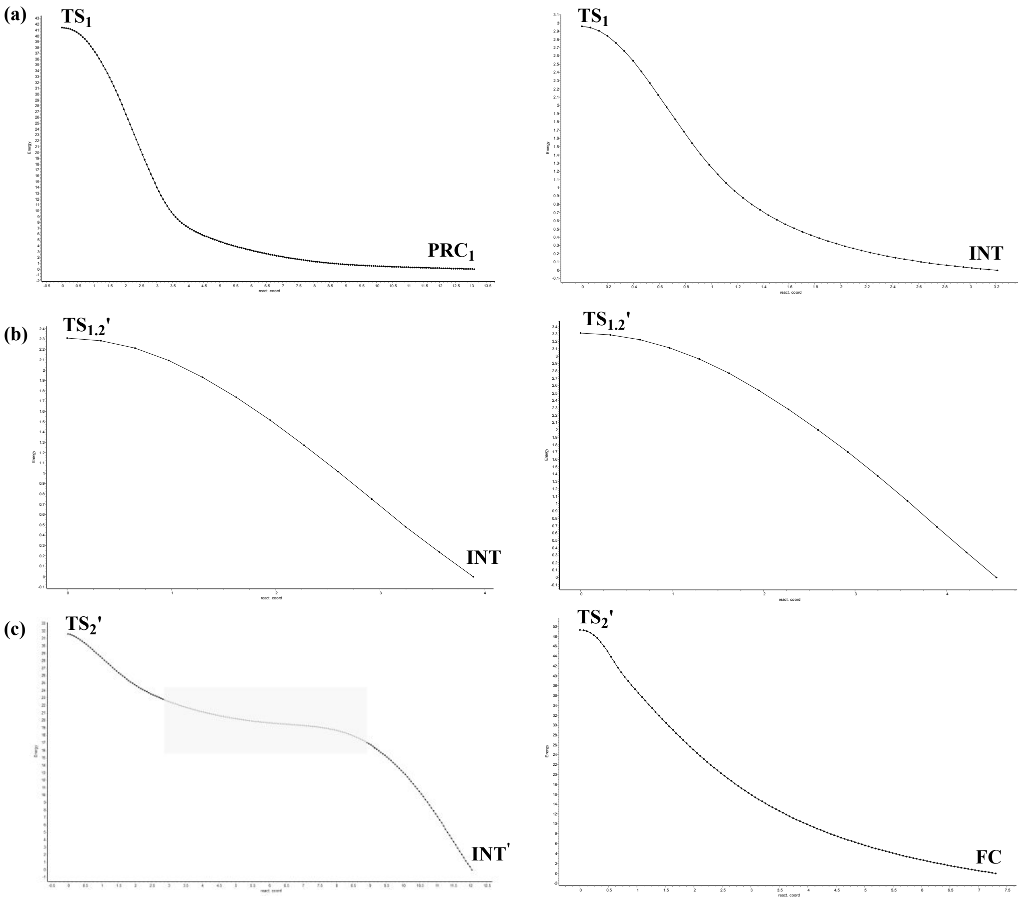

Figure S9. Reaction pathway with anchimeric assistance. **a)** Intrinsic reaction coordinate (IRC) calculated from **TS$_1$** structure M06-2X/def2-TZVP/IEFPCM (1,4-dioxane) in implicit solvation; **b)** Intrinsic reaction coordinate (IRC) calculated from **TS$_{1.2}$'** structure M06-2X/def2-TZVP/IEFPCM (1,4-dioxane) in implicit solvation; **c)** Intrinsic reaction coordinate (IRC) calculated from **TS$_2$'** structure M06-2X/def2-TZVP/IEFPCM (1,4-dioxane) in implicit solvation.

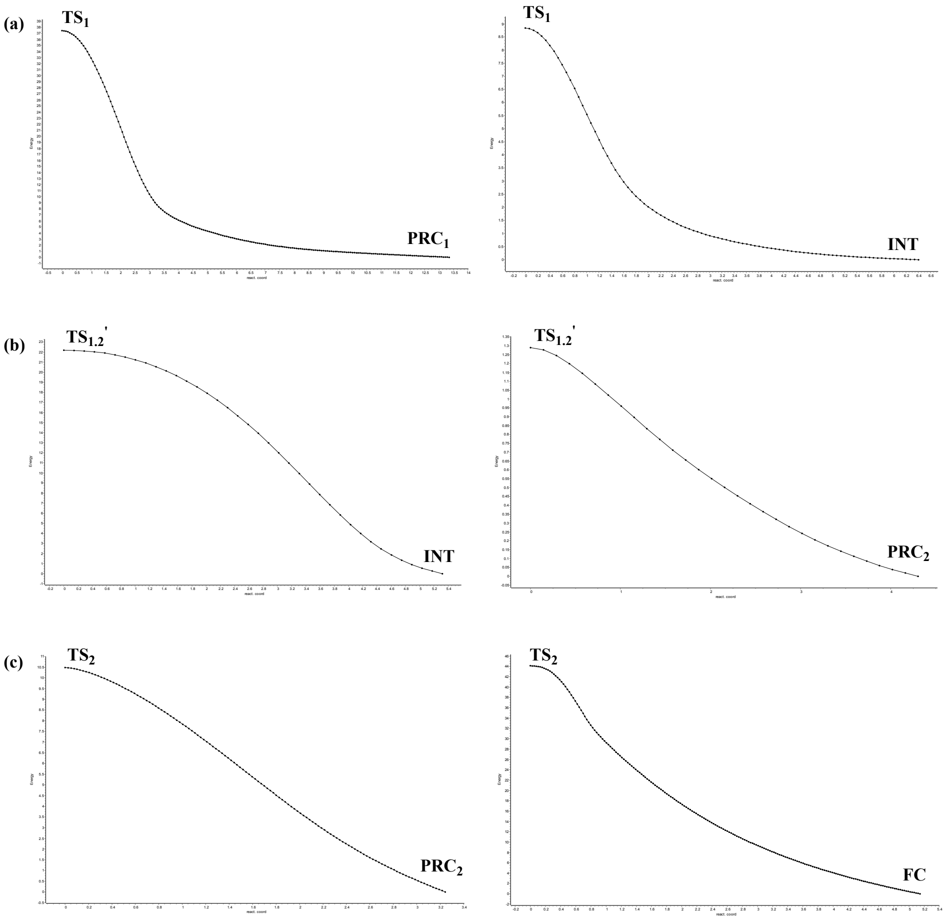

Figure S10. a) Intrinsic reaction coordinate (IRC) calculated from **TS$_1$** structure M06-2X/def2-TZVP/IEFPCM (acetonitrile) in implicit solvation; **b)** Intrinsic reaction coordinate (IRC) calculated from **TS$_{1,2}$'** structure M06-2X/def2-TZVP/IEFPCM (acetonitrile) in implicit solvation; **c)** Intrinsic reaction coordinate (IRC) calculated from **TS$_2$** structure M06-2X/def2-TZVP/IEFPCM (acetonitrile) in implicit solvation.

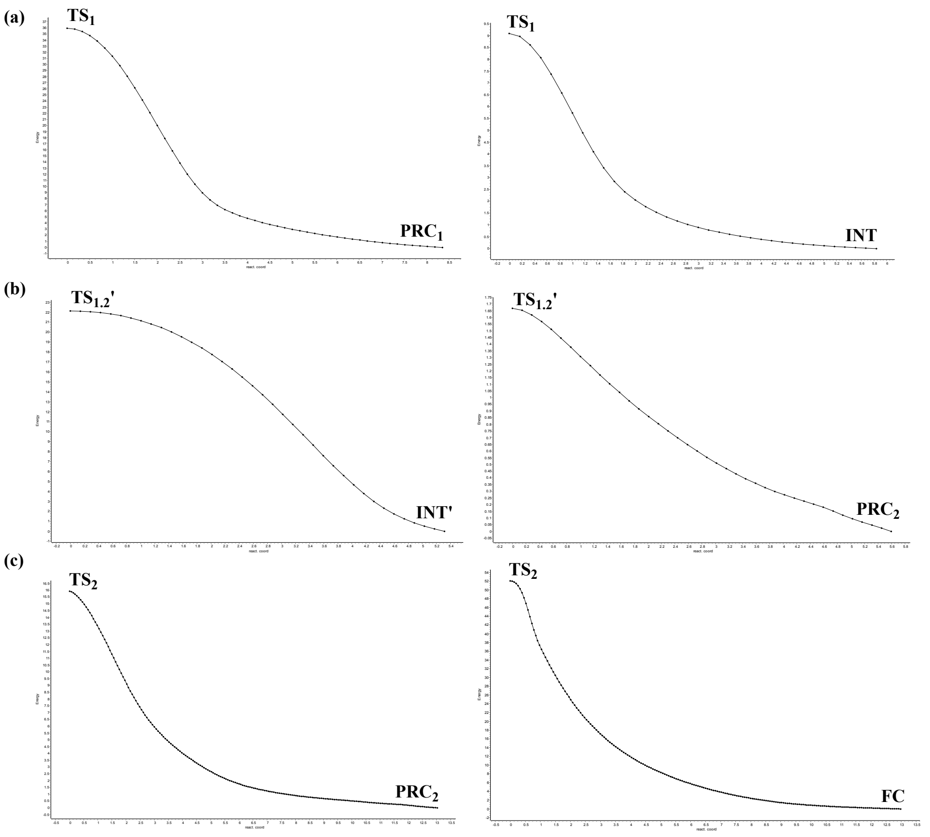

Figure S11. a) Intrinsic reaction coordinate (IRC) calculated from $\mathbf{TS_1}$ structure M06-2X/def2-TZVP/IEFPCM (water) in implicit solvation; **b)** Intrinsic reaction coordinate (IRC) calculated from $\mathbf{TS_{1.2}'}$ structure M06-2X/def2-TZVP/IEFPCM (water) in implicit solvation; **c)** Intrinsic reaction coordinate (IRC) calculated from $\mathbf{TS_2}$ structure M06-2X/def2-TZVP/IEFPCM (water) in implicit solvation.

S4.3 Explicit microsolvation calculations

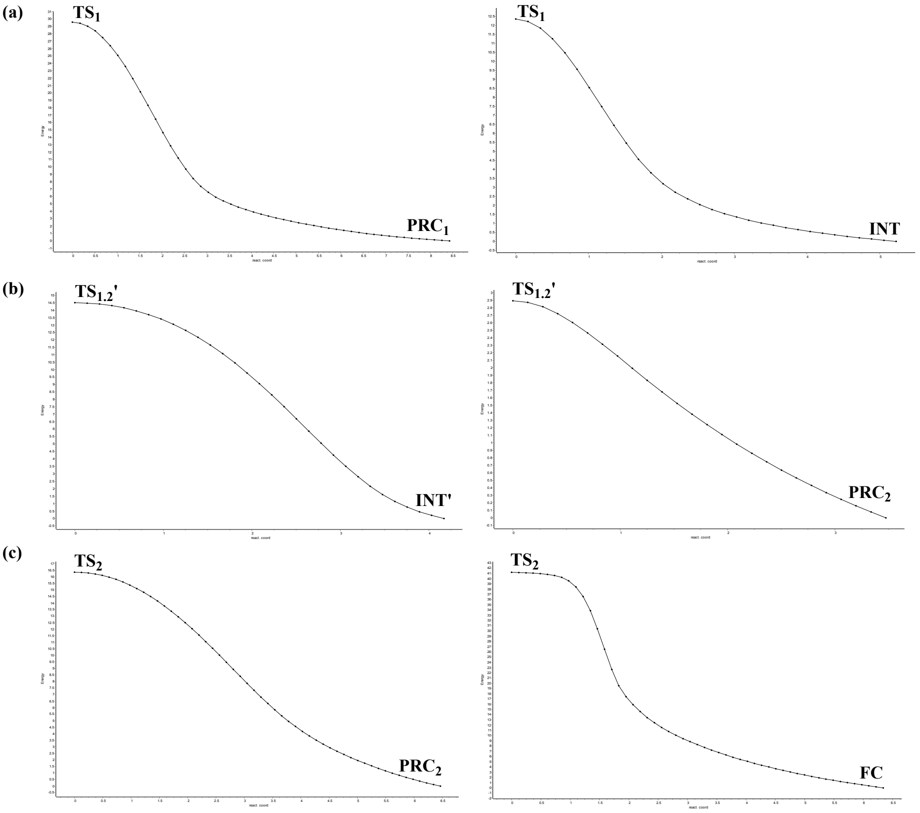

Figure S12. a) Intrinsic reaction coordinate (IRC) calculated from **TS$_1$** structure M06-2X/def2-TZVP/IEFPCM (water) in explicit microsolvation; **b)** Intrinsic reaction coordinate (IRC) calculated from **TS$_{1.2}$'** structure M06-2X/def2-TZVP/IEFPCM (water) in explicit microsolvation; **c)** Intrinsic reaction coordinate (IRC) calculated from **TS$_2$** structure M06-2X/def2-TZVP/IEFPCM (water) in explicit microsolvation.

S5 Variation of energy barriers for different functionals

Table S1. Energy barriers (in kcal mol^{-1}) at 25 °C and 1 mol L^{-1} standard state for the epoxide ring-opening (first stage) and intramolecular elimination (final stage) relative to its respective pre-reactive conformer, calculated using the implicit and explicit microsolvation approach (IEFPCM, solvent = water) with different functionals. For consistency, we maintained the def2-TZVP basis set.

def2-TZVP				
**Functional**	**Solvation model**	**Energy barrier (kcal mol^{-1})**	**Epoxide ring-opening (first stage)**	**Intramolecular elimination (final stage)**
			**$TS_1 - PRC_1$**	**$TS_2 - PRC_2$**
**M06-2X**	**Implicit solvation**	$\Delta G^{\ddagger}$	39.0	13.2
	**Explicit microsolvation**	$\Delta G^{\ddagger}$	33.0	15.7
**ωB97X-D**	**Implicit solvation**	$\Delta G^{\ddagger}$	35.0	12.3
	**Explicit microsolvation**	$\Delta G^{\ddagger}$	29.5	12.9
**CAM-B3LYP**	**Implicit solvation**	$\Delta G^{\ddagger}$	34.0	13.1
	**Explicit microsolvation**	$\Delta G^{\ddagger}$	28.1	15.8
**B3LYP-D3**	**Implicit solvation**	$\Delta G^{\ddagger}$	30.4	8.3
	**Explicit microsolvation**	$\Delta G^{\ddagger}$	23.7	10.9

To confirm our tendencies, we performed full geometry optimizations and frequency calculations, varying only the functional, for the main activation free energy (**Table S1**). Since the barriers are calculated using the pre-reactive complexes as the reference point, the standard-state entropy correction cancels out.

S6 Optimized cartesian coordinate matrices

S6.1 Gas phase calculations

PRC_1

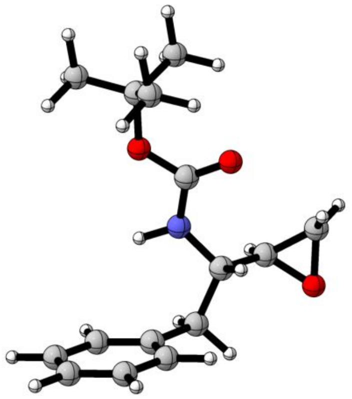

6	-1.353876000	0.274636000	0.005129000
7	-0.166279000	0.480826000	-0.627985000
6	0.774773000	1.465410000	-0.128404000
6	0.357091000	2.859531000	-0.530610000
8	-1.781641000	0.980419000	0.888790000
8	-1.963869000	-0.806335000	-0.505402000
6	-3.263121000	-1.228864000	-0.005859000
6	-3.564844000	-2.459210000	-0.847928000
6	-3.166859000	-1.603171000	1.467045000
6	-4.302846000	-0.144839000	-0.255842000
1	0.182324000	-0.284657000	-1.183701000
6	2.172472000	1.168238000	-0.678130000
6	-0.363903000	3.731856000	0.390708000
8	1.028761000	3.899837000	0.152629000
6	2.679715000	-0.193090000	-0.283925000
6	3.031389000	-0.456939000	1.038859000
6	3.463992000	-1.717030000	1.419561000
6	3.551325000	-2.738652000	0.481280000
6	3.207248000	-2.488248000	-0.837728000
6	2.775611000	-1.222249000	-1.215099000
1	0.796722000	1.425676000	0.965776000
1	0.243378000	3.012120000	-1.602139000
1	-4.531030000	-2.875699000	-0.562890000
1	-3.594040000	-2.196828000	-1.905710000
1	-2.797190000	-3.218662000	-0.697814000
1	-4.106005000	-2.061119000	1.781066000
1	-2.976728000	-0.728975000	2.084431000
1	-2.365594000	-2.328578000	1.616111000
1	-4.296746000	0.142866000	-1.308282000
1	-5.292030000	-0.536337000	-0.013904000
1	-4.111490000	0.732936000	0.356133000
1	2.836234000	1.949497000	-0.303987000

1	2.147856000	1.256127000	-1.768192000
1	-0.628135000	3.340797000	1.365393000
1	-0.993248000	4.521629000	-0.002831000
1	2.968970000	0.337796000	1.773833000
1	3.736744000	-1.903851000	2.450238000
1	3.889975000	-3.722452000	0.778502000
1	3.277687000	-3.275741000	-1.576951000
1	2.521283000	-1.027966000	-2.251653000

$E = -864.631422$ a.u. (M06-2X/def2-TZVP)

$E = -864.654735$ a.u. (ωB97M-V/def2-TZVP)

$G_{vrt} = 0.293168$ a.u. (M06-2X/def2-TZVP)

TS$_1$

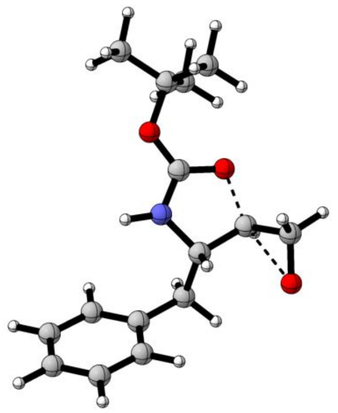

6	1.381855000	-0.121471000	-0.043957000
7	0.081379000	-0.256132000	-0.187113000
6	-0.642125000	1.012497000	-0.008228000
6	0.453402000	1.993849000	0.416345000
8	1.793568000	1.023560000	0.303386000
8	2.150632000	-1.148547000	-0.261978000
6	3.622446000	-1.075950000	-0.127299000
6	4.045859000	-2.491981000	-0.475321000
6	4.175808000	-0.077850000	-1.130574000
6	3.987970000	-0.734226000	1.307538000
1	-0.349353000	-1.116570000	-0.487951000
6	-1.783343000	0.869225000	0.992000000
6	0.456252000	3.232484000	-0.420127000
8	-0.797716000	3.606018000	-0.083490000
6	-2.813890000	-0.106756000	0.493922000
6	-3.717026000	0.283234000	-0.494916000
6	-4.634512000	-0.619182000	-1.010004000
6	-4.662664000	-1.929288000	-0.546454000
6	-3.769898000	-2.328652000	0.436642000
6	-2.851759000	-1.420982000	0.952019000

1	-1.042694000	1.346765000	-0.965134000
1	0.472403000	2.220084000	1.477027000
1	5.130799000	-2.571161000	-0.414312000
1	3.603208000	-3.205222000	0.219709000
1	3.732941000	-2.746135000	-1.487748000
1	5.264122000	-0.146034000	-1.124572000
1	3.892318000	0.942486000	-0.883983000
1	3.825071000	-0.316898000	-2.135060000
1	3.499739000	-1.423717000	1.997267000
1	5.066599000	-0.841441000	1.426292000
1	3.712816000	0.286554000	1.561365000
1	-2.194213000	1.873169000	1.102533000
1	-1.380099000	0.545774000	1.955134000
1	0.625999000	2.972243000	-1.490184000
1	1.274923000	3.915962000	-0.125370000
1	-3.694417000	1.307322000	-0.851493000
1	-5.333667000	-0.299558000	-1.772050000
1	-5.382002000	-2.632109000	-0.946036000
1	-3.791933000	-3.344563000	0.810064000
1	-2.164330000	-1.731982000	1.731423000

$E = -864.552098$ a.u. (M06-2X/def2-TZVP)

$E = -864.581995$ a.u. (ωB97M-V/def2-TZVP)

$G_{vrt} = 0.292853$ a.u. (M06-2X/def2-TZVP)

$f = 353.3944i$ (M06-2X/def2-TZVP)

INT

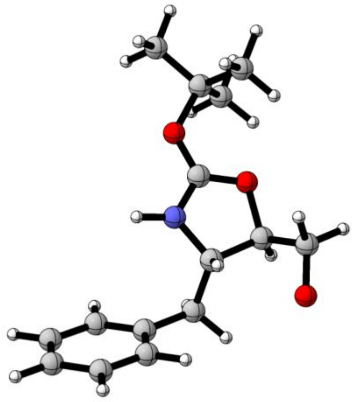

6	1.387447000	-0.199732000	0.002400000
7	0.092368000	-0.364831000	-0.103002000
6	-0.622943000	0.916799000	0.055431000
6	0.510505000	1.855492000	0.486536000
8	1.763106000	0.975687000	0.342395000
8	2.203520000	-1.176490000	-0.228755000

6	3.679828000	-1.022391000	-0.153259000
6	4.161304000	-2.418125000	-0.505233000
6	4.132079000	-0.007249000	-1.188713000
6	4.080089000	-0.645722000	1.262850000
1	-0.328130000	-1.225444000	-0.418711000
6	-1.782428000	0.819002000	1.033171000
6	0.478683000	3.114446000	-0.368392000
8	-0.775915000	3.543599000	-0.201829000
6	-2.852785000	-0.095215000	0.503587000
6	-3.698498000	0.350563000	-0.512333000
6	-4.652153000	-0.494662000	-1.057359000
6	-4.774275000	-1.800957000	-0.597406000
6	-3.938513000	-2.254671000	0.411650000
6	-2.983154000	-1.404583000	0.957469000
1	-0.997160000	1.254576000	-0.911502000
1	0.481749000	2.097407000	1.547322000
1	5.250528000	-2.437669000	-0.491763000
1	3.789421000	-3.145163000	0.216361000
1	3.818961000	-2.701092000	-1.500216000
1	5.221967000	-0.010563000	-1.222244000
1	3.798668000	0.998908000	-0.945595000
1	3.760293000	-0.281363000	-2.176406000
1	3.650372000	-1.347729000	1.978267000
1	5.166097000	-0.703440000	1.341064000
1	3.771039000	0.365223000	1.516606000
1	-2.141093000	1.844718000	1.137854000
1	-1.416547000	0.470517000	2.002504000
1	0.764415000	2.801285000	-1.409257000
1	1.289362000	3.797851000	-0.033494000
1	-3.599357000	1.371549000	-0.865440000
1	-5.306459000	-0.133188000	-1.840239000
1	-5.522393000	-2.458567000	-1.020472000
1	-4.034106000	-3.267572000	0.781686000
1	-2.341010000	-1.756765000	1.757820000

$E = -864.552871$ a.u. (M06-2X/def2-TZVP)

$E = -864.581346$ a.u. (ωB97M-V/def2-TZVP)

$G_{vrt} = 0.293326$ a.u. (M06-2X/def2-TZVP)

$TS_{1.2}$'

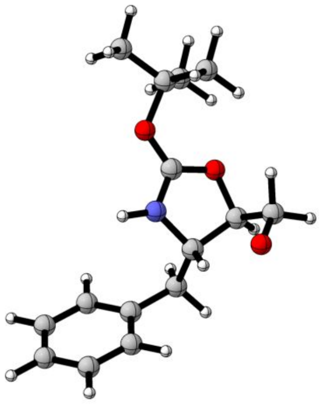

6	1.243507000	-0.168445000	0.015728000
7	-0.013738000	-0.084704000	-0.325838000
6	-0.646584000	1.103585000	0.262291000
6	0.585161000	1.828468000	0.807442000
8	1.647631000	0.776527000	0.793686000
8	1.996498000	-1.139348000	-0.381918000
6	3.472601000	-1.139598000	-0.197860000
6	3.878809000	-2.411998000	-0.918424000
6	4.061869000	0.087788000	-0.869929000
6	3.796733000	-1.221078000	1.283638000
1	-0.458616000	-0.727097000	-0.962375000
6	-1.729290000	0.724042000	1.262613000
6	0.977276000	3.028892000	-0.179366000
8	0.036443000	3.251076000	-1.078080000
6	-2.826885000	-0.067297000	0.599960000
6	-3.659824000	0.543541000	-0.337479000
6	-4.642231000	-0.185031000	-0.989326000
6	-4.804791000	-1.538706000	-0.718411000
6	-3.978650000	-2.157004000	0.207399000
6	-2.994951000	-1.423371000	0.860553000
1	-0.996437000	1.774762000	-0.542573000
1	0.498721000	2.115024000	1.853102000
1	4.959538000	-2.533402000	-0.852999000
1	3.400955000	-3.279370000	-0.463362000
1	3.595465000	-2.362842000	-1.969329000
1	5.148528000	-0.002380000	-0.860664000
1	3.787394000	1.006715000	-0.356531000
1	3.732105000	0.149365000	-1.907390000
1	3.275784000	-2.062915000	1.740916000
1	4.869300000	-1.383775000	1.393935000
1	3.532937000	-0.304991000	1.806139000
1	-2.127610000	1.655426000	1.671986000
1	-1.300688000	0.153190000	2.091391000
1	1.972570000	2.707533000	-0.586370000
1	1.207295000	3.881590000	0.500385000
1	-3.530167000	1.597704000	-0.555896000
1	-5.283676000	0.304159000	-1.710884000

1	-5.573328000	-2.106222000	-1.226654000
1	-4.100197000	-3.210129000	0.426372000
1	-2.354789000	-1.908181000	1.589183000

$E = -864.547156$ a.u. (M06-2X/def2-TZVP)

$E = -864.576430$ a.u. (ωB97M-V/def2-TZVP)

$G_{vrt} = 0.29337$ a.u. (M06-2X/def2-TZVP)

$f = 127.133i$ (M06-2X/def2-TZVP)

INT'

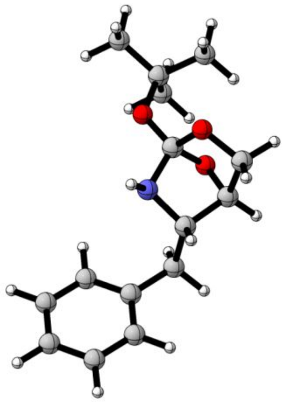

6	1.304929000	0.248706000	-0.342607000
7	-0.108764000	0.031984000	-0.578613000
6	-0.749964000	1.234788000	-0.001282000
6	0.493270000	1.965079000	0.536107000
8	1.315025000	0.864679000	0.927180000
8	2.003644000	-0.887441000	-0.472151000
6	3.347046000	-1.031286000	0.064203000
6	3.904590000	-2.207607000	-0.722897000
6	4.214911000	0.202692000	-0.145768000
6	3.240652000	-1.382966000	1.543617000
1	-0.288435000	-0.079173000	-1.568148000
6	-1.747326000	0.860000000	1.095541000
6	1.311822000	2.492462000	-0.648364000
8	1.797364000	1.278405000	-1.213934000
6	-2.915908000	0.087891000	0.547637000
6	-4.079985000	0.746616000	0.159740000
6	-5.146204000	0.047750000	-0.389146000
6	-5.060112000	-1.326724000	-0.559122000
6	-3.903527000	-1.993813000	-0.177635000
6	-2.839619000	-1.292007000	0.370888000
1	-1.254075000	1.831673000	-0.765833000
1	0.333368000	2.650247000	1.363345000
1	4.902454000	-2.459711000	-0.363125000

1	3.257772000	-3.077067000	-0.604866000
1	3.963174000	-1.958002000	-1.782353000
1	5.223512000	-0.029553000	0.200222000
1	3.843392000	1.052787000	0.424379000
1	4.255435000	0.476565000	-1.198458000
1	2.585558000	-2.244760000	1.673632000
1	4.228926000	-1.633913000	1.932464000
1	2.840259000	-0.548290000	2.115421000
1	-2.103259000	1.775627000	1.575719000
1	-1.213243000	0.269930000	1.842771000
1	2.152299000	3.108027000	-0.319820000
1	0.711838000	3.041855000	-1.375270000
1	-4.154353000	1.820001000	0.297032000
1	-6.045657000	0.575882000	-0.678973000
1	-5.890831000	-1.875804000	-0.983106000
1	-3.830040000	-3.066202000	-0.306304000
1	-1.932460000	-1.808648000	0.657337000

$E = -864.618688$ a.u. (M06-2X/def2-TZVP)

$E = -864.6469563$ a.u. (ωB97M-V/def2-TZVP)

$G_{vrt} = 0.300502$ a.u. (M06-2X/def2-TZVP)

PRC$_2$

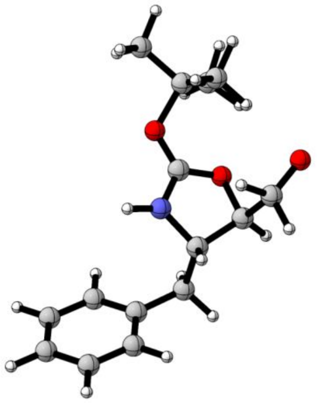

6	1.055923000	-0.327271000	0.111537000
7	-0.167249000	-0.172720000	-0.352118000
6	-0.692169000	1.146323000	0.030274000
6	0.605071000	1.815456000	0.506364000
8	1.467538000	0.653524000	0.826262000
8	1.761953000	-1.371741000	-0.115662000
6	3.276032000	-1.256529000	-0.133118000
6	3.670059000	-2.629396000	-0.640114000
6	3.653432000	-0.145917000	-1.095858000
6	3.783914000	-1.013109000	1.275758000
1	-0.612623000	-0.832460000	-0.969015000

6	-1.819138000	1.029375000	1.055380000
6	1.364655000	2.584104000	-0.604645000
8	2.621396000	2.796441000	-0.299699000
6	-2.977010000	0.235717000	0.512755000
6	-3.901081000	0.830670000	-0.344212000
6	-4.933419000	0.089219000	-0.898786000
6	-5.055721000	-1.262916000	-0.605621000
6	-4.142035000	-1.866147000	0.246266000
6	-3.109911000	-1.119956000	0.800580000
1	-1.041940000	1.659280000	-0.870428000
1	0.523861000	2.381575000	1.430521000
1	4.755140000	-2.675404000	-0.726548000
1	3.341023000	-3.407982000	0.048728000
1	3.235742000	-2.815154000	-1.621966000
1	4.740848000	-0.148225000	-1.191823000
1	3.355619000	0.857756000	-0.750928000
1	3.232654000	-0.345625000	-2.083519000
1	3.326252000	-1.711040000	1.978258000
1	4.860706000	-1.186834000	1.277929000
1	3.602651000	0.010555000	1.592794000
1	-2.134867000	2.041825000	1.315617000
1	-1.426116000	0.562466000	1.961639000
1	0.711237000	3.483430000	-0.780016000
1	1.183045000	1.947227000	-1.528338000
1	-3.812979000	1.887230000	-0.572189000
1	-5.646458000	0.567891000	-1.557391000
1	-5.863173000	-1.841254000	-1.034895000
1	-4.235293000	-2.917511000	0.485954000
1	-2.403297000	-1.592227000	1.473868000

$E = -864.550858$ a.u. (M06-2X/def2-TZVP)

$E = -864.5793747$ a.u. (ωB97M-V/def2-TZVP)

$G_{vrt} = 0.294532$ a.u. (M06-2X/def2-TZVP)

TS$_2$

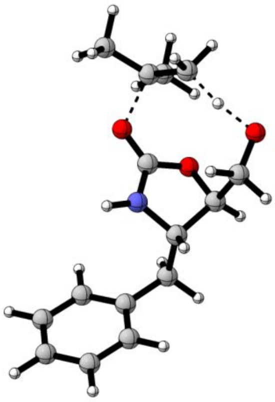

6	1.004330000	-0.375531000	0.103521000
7	-0.198244000	-0.154366000	-0.422536000
6	-0.725270000	1.141404000	0.011418000
6	0.583985000	1.769417000	0.506432000
8	1.363146000	0.596977000	0.897869000
8	1.723099000	-1.370508000	-0.119494000
6	3.471435000	-1.136703000	-0.142018000
6	3.750360000	-2.440527000	-0.842655000
6	3.780481000	0.100397000	-0.858080000
6	3.817380000	-1.143537000	1.323835000
1	-0.626512000	-0.779022000	-1.084532000
6	-1.830715000	0.990907000	1.057020000
6	1.384986000	2.455417000	-0.622726000
8	2.704944000	2.465423000	-0.365164000
6	-3.005332000	0.224157000	0.512518000
6	-3.967781000	0.865095000	-0.264864000
6	-5.020326000	0.153388000	-0.820814000
6	-5.124452000	-1.214990000	-0.608127000
6	-4.172280000	-1.864128000	0.164402000
6	-3.120276000	-1.147751000	0.720002000
1	-1.093141000	1.692556000	-0.857541000
1	0.487103000	2.382432000	1.398735000
1	4.834729000	-2.540475000	-0.922069000
1	3.355936000	-3.294150000	-0.291707000
1	3.335665000	-2.431816000	-1.849123000
1	4.742286000	0.458347000	-0.478732000
1	3.166953000	1.209769000	-0.590059000
1	3.850421000	-0.055453000	-1.934773000
1	3.310471000	-1.952223000	1.848963000
1	4.894582000	-1.305626000	1.405960000
1	3.576254000	-0.187395000	1.782761000
1	-2.138782000	1.992187000	1.365939000
1	-1.416865000	0.486294000	1.933306000
1	0.934763000	3.459963000	-0.743116000
1	1.121675000	1.902406000	-1.556580000
1	-3.894174000	1.934606000	-0.428991000
1	-5.762918000	0.667759000	-1.416924000

1	-5.947125000	-1.770909000	-1.038174000
1	-4.250410000	-2.929213000	0.340420000
1	-2.381857000	-1.656695000	1.329112000

$E = -864.535086$ a.u. (M06-2X/def2-TZVP)

$E = -864.564464$ a.u. (ωB97M-V/def2-TZVP)

$G_{vrt} = 0.289783$ a.u. (M06-2X/def2-TZVP)

$f = 1186.1646i$ (M06-2X/def2-TZVP)

TS$_2$'

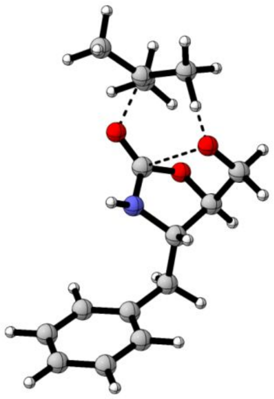

6	-1.118005000	0.348486000	0.212069000
7	0.155045000	0.269134000	-0.192712000
6	0.699621000	-1.046176000	0.150217000
6	-0.553620000	-1.733365000	0.723516000
8	-1.413433000	-0.608168000	1.070563000
8	-1.909856000	1.258138000	-0.090455000
6	-3.675578000	0.894639000	-0.137678000
6	-4.016733000	2.072281000	-1.006861000
6	-3.867833000	-0.420848000	-0.716662000
6	-4.003107000	1.079055000	1.317540000
1	0.407173000	0.734481000	-1.049765000
6	1.873627000	-0.938400000	1.122675000
6	-1.321854000	-2.487481000	-0.365602000
8	-1.613656000	-1.630285000	-1.383663000
6	3.025626000	-0.178862000	0.523135000
6	3.947260000	-0.826095000	-0.296820000
6	4.980892000	-0.122777000	-0.897428000
6	5.106340000	1.243897000	-0.686772000
6	4.193314000	1.899536000	0.126778000
6	3.160278000	1.191741000	0.726239000
1	1.001478000	-1.556760000	-0.765987000
1	-0.367152000	-2.295066000	1.637390000
1	-5.105789000	2.132990000	-1.065484000

1	-3.633097000	3.003399000	-0.593403000
1	-3.625776000	1.929998000	-2.012875000
1	-4.318779000	-1.120979000	-0.014657000
1	-2.786918000	-0.977531000	-1.063459000
1	-4.420276000	-0.375131000	-1.653628000
1	-3.614364000	2.021108000	1.701806000
1	-5.092660000	1.089034000	1.404849000
1	-3.621410000	0.251190000	1.910975000
1	2.187879000	-1.950677000	1.388384000
1	1.527008000	-0.447384000	2.035275000
1	-2.217169000	-2.926540000	0.115299000
1	-0.686089000	-3.334930000	-0.681448000
1	3.855239000	-1.894086000	-0.461473000
1	5.691222000	-0.642142000	-1.527651000
1	5.913827000	1.793883000	-1.152005000
1	4.286432000	2.964228000	0.298262000
1	2.448102000	1.705701000	1.360959000

$E = -864.553019$ a.u. (M06-2X/def2-TZVP)

$E = -864.583127$ a.u. (ωB97M-V/def2-TZVP)

$G_{vrt} = 0.291734$ a.u. (M06-2X/def2-TZVP)

$f = 1016.1408i$ (M06-2X/def2-TZVP)

FC

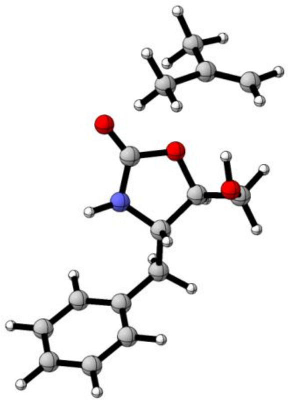

6	0.856535000	-1.357501000	0.506990000
7	-0.436594000	-0.980897000	0.292787000
6	-0.629392000	0.443339000	0.475091000
6	0.635808000	0.788823000	1.280054000
8	1.514647000	-0.324759000	1.095372000
8	1.379801000	-2.404960000	0.253433000
6	4.385907000	-0.162333000	-0.745937000
6	3.708822000	-0.787683000	-1.930555000
6	4.717354000	1.127398000	-0.716758000

6	4.681984000	-1.096942000	0.388124000
1	-0.997178000	-1.527047000	-0.340968000
6	-1.926384000	0.779996000	1.205015000
6	1.335639000	2.046588000	0.804898000
8	1.659037000	1.972231000	-0.557870000
6	-3.131807000	0.359109000	0.407451000
6	-3.630838000	1.171361000	-0.608311000
6	-4.706822000	0.760289000	-1.380801000
6	-5.298943000	-0.474259000	-1.149033000
6	-4.809205000	-1.292264000	-0.141100000
6	-3.732817000	-0.876641000	0.631552000
1	-0.603711000	0.963383000	-0.487953000
1	0.405007000	0.864262000	2.347611000
1	4.343877000	-1.568857000	-2.356239000
1	2.783518000	-1.278684000	-1.618745000
1	3.488918000	-0.056834000	-2.708278000
1	5.211446000	1.562172000	0.143801000
1	2.461642000	1.436451000	-0.641156000
1	4.529073000	1.780688000	-1.561524000
1	3.774046000	-1.616916000	0.698953000
1	5.391942000	-1.860716000	0.058797000
1	5.108168000	-0.572162000	1.241952000
1	-1.950506000	1.858513000	1.384040000
1	-1.921802000	0.277774000	2.174742000
1	2.225843000	2.214755000	1.419325000
1	0.662519000	2.896669000	0.944213000
1	-3.172667000	2.137011000	-0.791714000
1	-5.085733000	1.405248000	-2.163053000
1	-6.140013000	-0.795001000	-1.749503000
1	-5.267791000	-2.254227000	0.048181000
1	-3.353353000	-1.515975000	1.420458000

E = −864.640986 a.u. (M06-2X/def2-TZVP)

E = −864.668011 a.u. (ωB97M-V/def2-TZVP)

G_{vrt} = 0.290621 a.u. (M06-2X/def2-TZVP)

P

Oxazolidin-2-one

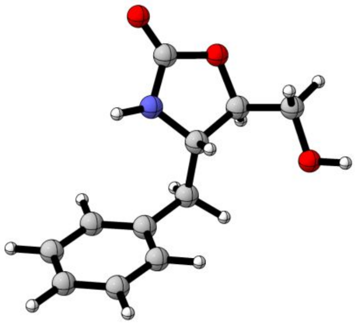

6	-0.772003000	0.175345000	0.046237000
6	-2.228328000	0.403660000	-0.396964000
8	-2.927340000	-0.787003000	-0.047611000
6	-2.045236000	-1.777345000	0.270981000
7	-0.780613000	-1.266301000	0.178559000
8	-2.381288000	-2.885367000	0.561237000
6	-2.890718000	1.576985000	0.288455000
6	0.265088000	0.678886000	-0.953358000
8	-2.119148000	2.715684000	-0.041983000
6	1.663429000	0.375099000	-0.487269000
6	2.308934000	1.225113000	0.408362000
6	3.575221000	0.923183000	0.886647000
6	4.215776000	-0.238653000	0.476016000
6	3.582953000	-1.092808000	-0.415568000
6	2.315485000	-0.786342000	-0.893468000
1	-0.594925000	0.661197000	1.013728000
1	-2.275047000	0.533224000	-1.482542000
1	-0.019817000	-1.780775000	0.590884000
1	-3.923303000	1.669762000	-0.059406000
1	-2.900423000	1.394306000	1.368660000
1	0.121768000	1.754659000	-1.069861000
1	0.074617000	0.201223000	-1.916825000
1	-2.524634000	3.498054000	0.339141000
1	1.812212000	2.135165000	0.726537000
1	4.065266000	1.596950000	1.577705000
1	5.204946000	-0.474170000	0.846145000
1	4.077703000	-1.997657000	-0.744326000
1	1.824514000	-1.453065000	-1.593306000

E = −707.430634 a.u. (M06-2X/def2-TZVP)

E = −707.468151 a.u. (ωB97M-V/def2-TZVP)

G_{vrt} = 0.190032 a.u. (M06-2X/def2-TZVP)

Isobutylene

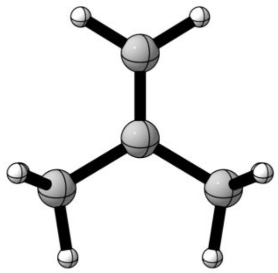

6	0.000000000	1.298003000	-0.669874000
6	0.000000000	0.000000000	0.106607000
6	0.000000000	-1.298003000	-0.669874000
6	0.000000000	0.000000000	1.433728000
1	0.000626000	1.126682000	-1.744991000
1	-0.879549000	1.896538000	-0.428400000
1	0.878883000	1.897127000	-0.427453000
1	-0.000626000	-1.126682000	-1.744991000
1	0.879549000	-1.896538000	-0.428400000
1	-0.878883000	-1.897127000	-0.427453000
1	-0.000045000	-0.924313000	1.999084000
1	0.000045000	0.924313000	1.999084000

$E = -157.188796$ a.u. (M06-2X/def2-TZVP)

$E = -157.177369$ a.u. (ωB97M-V/def2-TZVP)

$G_{vrt} = 0.082073$ a.u. (M06-2X/def2-TZVP)

S6.2 Implicit solvation calculations

PRC$_1$ (IEFPCM, solvent=1,4-dioxane)

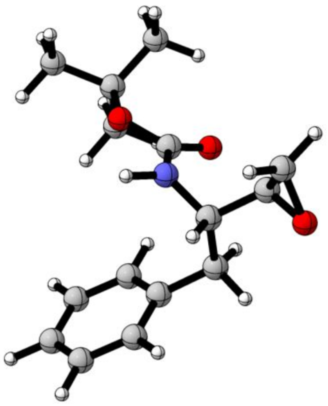

6	-0.996409000	0.702753000	0.035179000
7	0.106929000	0.879735000	0.806029000
6	1.381091000	1.320533000	0.256182000

6	1.313531000	2.760401000	-0.193149000
8	-1.097190000	1.083002000	-1.112027000
8	-1.946823000	0.065691000	0.734650000
6	-3.231227000	-0.259587000	0.130347000
6	-3.971230000	-0.953766000	1.263475000
6	-3.963604000	1.009734000	-0.282041000
6	-3.031424000	-1.215906000	-1.037337000
1	0.113861000	0.385357000	1.682627000
6	1.903413000	0.424919000	-0.881397000
6	1.627480000	3.845158000	0.734261000
8	2.567767000	3.419693000	-0.243769000
6	1.952394000	-1.022055000	-0.478717000
6	3.023421000	-1.523503000	0.257561000
6	3.039767000	-2.846452000	0.677507000
6	1.979650000	-3.688625000	0.367414000
6	0.907299000	-3.199954000	-0.366841000
6	0.895921000	-1.876293000	-0.785802000
1	2.093047000	1.274170000	1.083180000
1	0.657929000	2.959348000	-1.034438000
1	-4.961874000	-1.258046000	0.925710000
1	-3.423553000	-1.838781000	1.588200000
1	-4.082355000	-0.279444000	2.112944000
1	-4.982890000	0.752151000	-0.573275000
1	-3.470649000	1.498318000	-1.118301000
1	-4.013311000	1.702002000	0.559781000
1	-2.457083000	-2.085324000	-0.712113000
1	-4.005084000	-1.560739000	-1.388107000
1	-2.514513000	-0.729788000	-1.861549000
1	2.899545000	0.786902000	-1.143189000
1	1.258020000	0.552963000	-1.750395000
1	1.889705000	3.602760000	1.758748000
1	1.207311000	4.830175000	0.569709000
1	3.856044000	-0.870667000	0.496580000
1	3.882932000	-3.222180000	1.243121000
1	1.992373000	-4.721045000	0.691778000
1	0.079800000	-3.851699000	-0.617755000
1	0.061759000	-1.495786000	-1.366150000

$E = -864.635613$ a.u. (M06-2X/def2-TZVP)

$E = -864.667898$ a.u. (ωB97M-V/def2-TZVP)

$G_{vrt} = 0.293774$ a.u. (M06-2X/def2-TZVP)

TS$_1$ (IEFPCM, solvent=1,4-dioxane)

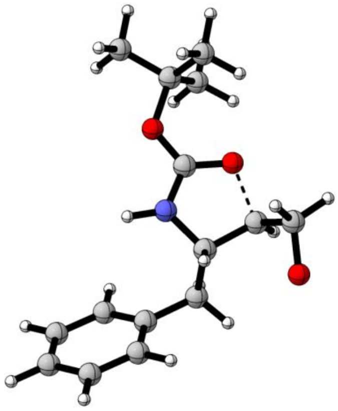

6	1.367173000	-0.075524000	-0.077326000
7	0.067195000	-0.162176000	-0.289896000
6	-0.655330000	1.090111000	-0.052763000
6	0.420033000	2.078185000	0.401681000
8	1.803224000	1.030251000	0.336599000
8	2.095188000	-1.130640000	-0.307602000
6	3.562194000	-1.135826000	-0.113159000
6	3.933773000	-2.554593000	-0.505694000
6	4.202478000	-0.125173000	-1.049485000
6	3.884737000	-0.872609000	1.348094000
1	-0.368841000	-1.002042000	-0.637847000
6	-1.779225000	0.898256000	0.963676000
6	0.535516000	3.293372000	-0.432678000
8	-0.705616000	3.715509000	-0.053335000
6	-2.789532000	-0.107955000	0.482797000
6	-3.737680000	0.254598000	-0.473622000
6	-4.641758000	-0.676244000	-0.962826000
6	-4.610889000	-1.987910000	-0.503951000
6	-3.672250000	-2.360337000	0.447389000
6	-2.768144000	-1.424496000	0.935895000
1	-1.073251000	1.454835000	-0.990751000
1	0.451049000	2.287097000	1.463216000
1	5.010769000	-2.686185000	-0.407236000
1	3.432570000	-3.273967000	0.141627000
1	3.650235000	-2.751435000	-1.539358000
1	5.285138000	-0.245249000	-1.000891000
1	3.955284000	0.896191000	-0.770104000
1	3.884444000	-0.306414000	-2.076682000
1	3.336588000	-1.565474000	1.987559000
1	4.951453000	-1.037353000	1.502485000
1	3.648315000	0.149064000	1.635152000
1	-2.231132000	1.880274000	1.101953000
1	-1.346241000	0.578820000	1.914776000
1	0.660242000	3.046709000	-1.505408000
1	1.384683000	3.931485000	-0.136303000
1	-3.766014000	1.279265000	-0.827355000

1	-5.375963000	-0.377353000	-1.699925000
1	-5.319282000	-2.713224000	-0.882413000
1	-3.646741000	-3.377888000	0.815952000
1	-2.042616000	-1.715993000	1.687364000

$E = -864.563838$ a.u. (M06-2X/def2-TZVP)

$E = -864.60354$ a.u. (ωB97M-V/def2-TZVP)

$G_{vrt} = 0.292113$ a.u. (M06-2X/def2-TZVP)

$f = 520.4333i$ (M06-2X/def2-TZVP)

INT (IEFPCM, solvent=1,4-dioxane)

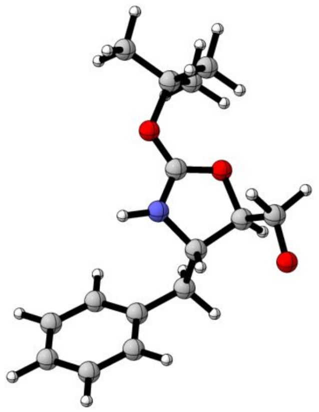

6	1.268789000	-0.172403000	-0.064449000
7	0.009225000	-0.137427000	-0.418196000
6	-0.648076000	1.084980000	0.068047000
6	0.566702000	1.877219000	0.572164000
8	1.664000000	0.852308000	0.608014000
8	2.028385000	-1.166554000	-0.364003000
6	3.505314000	-1.154200000	-0.139949000
6	3.921838000	-2.490929000	-0.723417000
6	4.112496000	0.001365000	-0.914581000
6	3.788638000	-1.092018000	1.350020000
1	-0.433784000	-0.870246000	-0.950968000
6	-1.711885000	0.774349000	1.114947000
6	0.888659000	3.032809000	-0.379047000
8	-0.196169000	3.808914000	-0.428425000
6	-2.805059000	-0.092681000	0.550402000
6	-3.760868000	0.455468000	-0.304511000
6	-4.740751000	-0.343395000	-0.873990000
6	-4.778874000	-1.705401000	-0.598858000
6	-3.832419000	-2.261376000	0.249232000
6	-2.851577000	-1.457804000	0.818835000
1	-1.080746000	1.644145000	-0.761404000

1	0.457279000	2.224823000	1.596219000
1	5.001171000	-2.601810000	-0.626240000
1	3.436948000	-3.309031000	-0.191398000
1	3.658020000	-2.546925000	-1.779007000
1	5.198045000	-0.082944000	-0.859835000
1	3.823153000	0.966338000	-0.503911000
1	3.818525000	-0.047685000	-1.963318000
1	3.243551000	-1.877936000	1.873513000
1	4.855344000	-1.258651000	1.501921000
1	3.529222000	-0.125220000	1.773896000
1	-2.110505000	1.735598000	1.445386000
1	-1.242982000	0.289545000	1.975243000
1	1.190328000	2.545440000	-1.348366000
1	1.823162000	3.508819000	-0.002173000
1	-3.734274000	1.518157000	-0.518411000
1	-5.479144000	0.097798000	-1.530965000
1	-5.546262000	-2.327800000	-1.040215000
1	-3.859576000	-3.319898000	0.474154000
1	-2.119337000	-1.893767000	1.489498000

$E = -864.569071$ a.u. (M06-2X/def2-TZVP)

$E = -864.607029$ a.u. (ωB97M-V/def2-TZVP)

$G_{vrt} = 0.293274$ a.u. (M06-2X/def2-TZVP)

$TS_{1,2}$' (IEFPCM, solvent=1,4-dioxane)

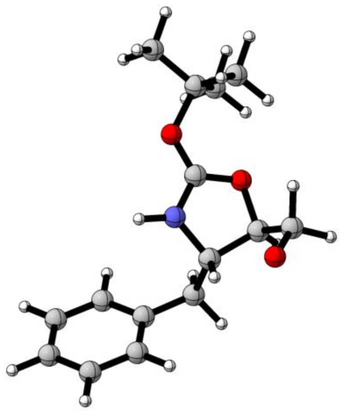

6	1.231308000	-0.137887000	-0.041018000
7	-0.018727000	-0.029919000	-0.404509000
6	-0.652170000	1.139032000	0.218319000
6	0.581514000	1.855764000	0.773052000
8	1.636309000	0.801624000	0.747355000
8	1.973857000	-1.115059000	-0.426867000
6	3.443460000	-1.172832000	-0.171668000
6	3.836996000	-2.445233000	-0.897909000
6	4.105949000	0.044402000	-0.790498000

6	3.686636000	-1.292871000	1.321878000
1	-0.470452000	-0.675362000	-1.034048000
6	-1.703483000	0.722879000	1.241109000
6	1.005486000	3.074106000	-0.156925000
8	0.102299000	3.348761000	-1.093591000
6	-2.805019000	-0.079986000	0.600375000
6	-3.736814000	0.543402000	-0.229043000
6	-4.723602000	-0.195022000	-0.863998000
6	-4.792099000	-1.571525000	-0.682339000
6	-3.868387000	-2.202375000	0.137713000
6	-2.880848000	-1.458776000	0.773485000
1	-1.042979000	1.805259000	-0.560020000
1	0.485041000	2.117315000	1.824303000
1	4.907921000	-2.606972000	-0.780790000
1	3.306271000	-3.302384000	-0.484235000
1	3.609231000	-2.364965000	-1.960324000
1	5.186502000	-0.091874000	-0.741119000
1	3.850597000	0.961089000	-0.263847000
1	3.820701000	0.141489000	-1.838244000
1	3.114794000	-2.124390000	1.734781000
1	4.746002000	-1.495018000	1.481848000
1	3.429505000	-0.377562000	1.849316000
1	-2.106035000	1.638021000	1.680986000
1	-1.233764000	0.146961000	2.043227000
1	2.010862000	2.765993000	-0.539178000
1	1.215338000	3.904815000	0.555023000
1	-3.684384000	1.616352000	-0.375680000
1	-5.441871000	0.304064000	-1.501535000
1	-5.563750000	-2.147141000	-1.176614000
1	-3.917001000	-3.273344000	0.287593000
1	-2.164701000	-1.953995000	1.419752000

$E = -864.563142$ a.u. (M06-2X/def2-TZVP)

$E = -864.601559$ a.u. (ωB97M-V/def2-TZVP)

$G_{vrt} = 0.294012$ a.u. (M06-2X/def2-TZVP)

$f = 136.9784i$ (M06-2X/def2-TZVP)

INT’ (IEFPCM, solvent=1,4-dioxane)

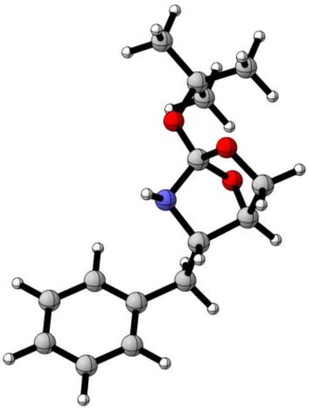

6	-1.306128000	-0.252480000	-0.343161000
7	0.107552000	-0.035839000	-0.583516000
6	0.749736000	-1.233984000	0.005724000
6	-0.491895000	-1.960889000	0.549825000
8	-1.315229000	-0.855594000	0.931490000
8	-2.006417000	0.883940000	-0.481599000
6	-3.350512000	1.033073000	0.055892000
6	-3.906882000	2.207133000	-0.735291000
6	-4.220228000	-0.200151000	-0.148759000
6	-3.244422000	1.391192000	1.533703000
1	0.285064000	0.057434000	-1.575689000
6	1.747316000	-0.852158000	1.099844000
6	-1.310701000	-2.499559000	-0.628377000
8	-1.798892000	-1.288470000	-1.204998000
6	2.917344000	-0.084897000	0.548168000
6	4.069305000	-0.751475000	0.136343000
6	5.137544000	-0.056720000	-0.414648000
6	5.066337000	1.321769000	-0.562973000
6	3.922270000	1.996793000	-0.157425000
6	2.856461000	1.298456000	0.393214000
1	1.252635000	-1.836633000	-0.754399000
1	-0.331533000	-2.638848000	1.382404000
1	-4.905463000	2.458849000	-0.377504000
1	-3.261749000	3.078096000	-0.617872000
1	-3.964484000	1.954909000	-1.794255000
1	-5.226422000	0.033554000	0.203022000
1	-3.846173000	-1.051059000	0.418426000
1	-4.268266000	-0.473138000	-1.201444000
1	-2.585111000	2.250198000	1.661477000
1	-4.232288000	1.649870000	1.918270000
1	-2.851219000	0.556871000	2.110870000
1	2.101045000	-1.765948000	1.584529000
1	1.215493000	-0.256707000	1.844313000
1	-2.150553000	-3.111671000	-0.293503000
1	-0.710941000	-3.053885000	-1.350937000
1	4.132021000	-1.827687000	0.255915000

1	6.027076000	-0.591080000	-0.723334000
1	5.898606000	1.867280000	-0.988683000
1	3.859967000	3.071880000	-0.268841000
1	1.960473000	1.822955000	0.700245000

$E = -864.622762$ a.u. (M06-2X/def2-TZVP)

$E = -864.660539$ a.u. (ωB97M-V/def2-TZVP)

$G_{vrt} = 0.300024$ a.u. (M06-2X/def2-TZVP)

PRC$_2$ (IEFPCM, solvent=1,4-dioxane)

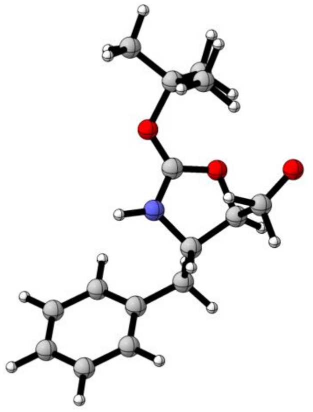

6	1.059768000	-0.310006000	0.039189000
7	-0.160423000	-0.140772000	-0.418603000
6	-0.686017000	1.166555000	-0.002727000
6	0.609756000	1.829862000	0.489178000
8	1.491864000	0.669167000	0.749876000
8	1.751868000	-1.362885000	-0.192027000
6	3.261463000	-1.308785000	-0.119405000
6	3.640555000	-2.665911000	-0.677491000
6	3.741010000	-0.170671000	-1.002628000
6	3.689675000	-1.163996000	1.328640000
1	-0.631248000	-0.808972000	-1.008552000
6	-1.797793000	1.026554000	1.035980000
6	1.335555000	2.680376000	-0.570356000
8	2.570583000	2.995740000	-0.224319000
6	-2.956025000	0.224003000	0.507000000
6	-3.899468000	0.812779000	-0.333183000
6	-4.937254000	0.063655000	-0.867909000
6	-5.045373000	-1.289307000	-0.570917000
6	-4.111765000	-1.886056000	0.264319000
6	-3.074048000	-1.132421000	0.798516000
1	-1.048958000	1.697103000	-0.886798000
1	0.520160000	2.346116000	1.441432000

1	4.726689000	-2.748455000	-0.698136000
1	3.241393000	-3.465736000	-0.053548000
1	3.263336000	-2.782628000	-1.692936000
1	4.831052000	-0.206517000	-1.028999000
1	3.445030000	0.818015000	-0.634458000
1	3.377043000	-0.308041000	-2.022340000
1	3.186630000	-1.903506000	1.952709000
1	4.763236000	-1.347439000	1.382297000
1	3.494899000	-0.164546000	1.708096000
1	-2.119894000	2.033472000	1.309312000
1	-1.386186000	0.556219000	1.932095000
1	0.620117000	3.531276000	-0.737229000
1	1.236742000	2.066890000	-1.515235000
1	-3.823167000	1.869742000	-0.563143000
1	-5.665766000	0.537008000	-1.513347000
1	-5.857228000	-1.873249000	-0.984188000
1	-4.193516000	-2.937796000	0.506631000
1	-2.352150000	-1.599516000	1.459090000

E = −864.566702 a.u. (M06-2X/def2-TZVP)

E = −864.605313 a.u. (ωB97M-V/def2-TZVP)

G_{vrt} = 0.294247 a.u. (M06-2X/def2-TZVP)

TS$_2$ (IEFPCM, solvent=1,4-dioxane)

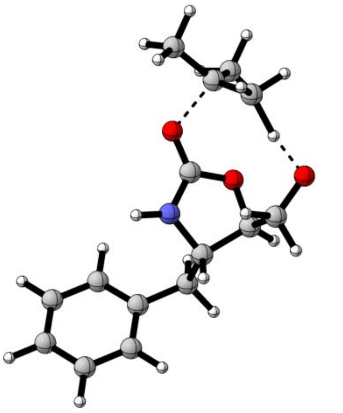

6	0.992509000	-0.385272000	0.076132000
7	-0.205301000	-0.149545000	-0.454007000
6	-0.732329000	1.139782000	-0.002910000
6	0.576814000	1.759311000	0.502551000
8	1.352425000	0.584422000	0.883395000
8	1.713979000	-1.373398000	-0.147079000
6	3.524123000	-1.128474000	-0.132522000
6	3.783250000	-2.392153000	-0.902492000
6	3.846594000	0.138324000	-0.771396000
6	3.806590000	-1.223040000	1.340194000

1	-0.641088000	-0.769597000	-1.116346000
6	-1.831340000	0.980270000	1.048004000
6	1.377975000	2.459123000	-0.612050000
8	2.703069000	2.490766000	-0.341130000
6	-3.011128000	0.218286000	0.507763000
6	-3.982331000	0.865936000	-0.253568000
6	-5.043221000	0.158964000	-0.800604000
6	-5.147070000	-1.210936000	-0.594708000
6	-4.185912000	-1.866429000	0.161877000
6	-3.125515000	-1.154866000	0.708433000
1	-1.103740000	1.701469000	-0.863086000
1	0.473349000	2.363763000	1.399996000
1	4.867496000	-2.512303000	-0.963892000
1	3.359526000	-3.266043000	-0.409841000
1	3.392982000	-2.314523000	-1.915609000
1	4.762979000	0.511691000	-0.304398000
1	3.190671000	1.214533000	-0.529014000
1	3.992667000	0.033600000	-1.846047000
1	3.291287000	-2.069322000	1.791291000
1	4.882499000	-1.375704000	1.456558000
1	3.531729000	-0.301268000	1.847775000
1	-2.136150000	1.979415000	1.366400000
1	-1.411881000	0.468327000	1.917282000
1	0.917391000	3.457369000	-0.735007000
1	1.139171000	1.908011000	-1.550654000
1	-3.909460000	1.936283000	-0.412240000
1	-5.792512000	0.678161000	-1.384154000
1	-5.976173000	-1.762955000	-1.017597000
1	-4.263261000	-2.932562000	0.332117000
1	-2.380216000	-1.668813000	1.304789000

$E = -864.544906$ a.u. (M06-2X/def2-TZVP)

$E = -864.58485$ a.u. (ωB97M-V/def2-TZVP)

$G_{vrt} = 0.289087$ a.u. (M06-2X/def2-TZVP)

$f = 1208.2898i$ (M06-2X/def2-TZVP)

TS_2’ (IEFPCM, solvent=1,4-dioxane)

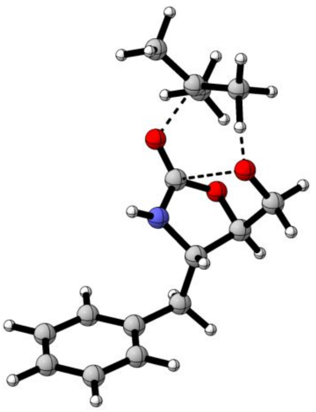

6	1.105410000	-0.368152000	0.201921000
7	-0.158274000	-0.262246000	-0.214403000
6	-0.699970000	1.047673000	0.145774000
6	0.554494000	1.719169000	0.735125000
8	1.411691000	0.586736000	1.061586000
8	1.892640000	-1.280540000	-0.099758000
6	3.691425000	-0.904207000	-0.142130000
6	4.022105000	-2.064544000	-1.034679000
6	3.874038000	0.422036000	-0.693000000
6	4.003306000	-1.117273000	1.310342000
1	-0.445522000	-0.757692000	-1.043013000
6	-1.873604000	0.934140000	1.118276000
6	1.330616000	2.511239000	-0.318464000
8	1.651497000	1.699438000	-1.368666000
6	-3.027779000	0.178833000	0.517426000
6	-3.937492000	0.825903000	-0.316630000
6	-4.975325000	0.125807000	-0.914454000
6	-5.117168000	-1.237153000	-0.686904000
6	-4.216109000	-1.892344000	0.140775000
6	-3.178726000	-1.187633000	0.737491000
1	-1.000484000	1.572318000	-0.762744000
1	0.361843000	2.259680000	1.660297000
1	5.111202000	-2.126763000	-1.097118000
1	3.639041000	-3.002193000	-0.636619000
1	3.630113000	-1.902273000	-2.037191000
1	4.320404000	1.109565000	0.024563000
1	2.802291000	0.993177000	-1.029751000
1	4.428489000	0.396102000	-1.629954000
1	3.614678000	-2.068102000	1.671246000
1	5.092525000	-1.126976000	1.405745000
1	3.615954000	-0.301834000	1.917035000
1	-2.184976000	1.945588000	1.389671000
1	-1.526121000	0.437338000	2.027327000
1	2.214450000	2.940566000	0.191405000

1	0.692609000	3.364959000	-0.611024000
1	-3.832957000	1.890611000	-0.494355000
1	-5.676280000	0.644628000	-1.555630000
1	-5.928001000	-1.784234000	-1.149887000
1	-4.321903000	-2.953673000	0.325542000
1	-2.477384000	-1.701102000	1.384753000

$E = -864.559803$ a.u. (M06-2X/def2-TZVP)

$E = -864.599644$ a.u. (ωB97M-V/def2-TZVP)

$G_{vrt} = 0.290893$ a.u. (M06-2X/def2-TZVP)

$f = 1026.0611i$ (M06-2X/def2-TZVP)

FC (IEFPCM, solvent=1,4-dioxane)

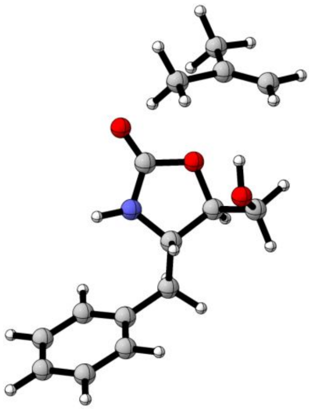

6	-0.842840000	-1.371927000	-0.511480000
7	0.435372000	-0.980285000	-0.270475000
6	0.629046000	0.440339000	-0.481566000
6	-0.631904000	0.769366000	-1.300569000
8	-1.505232000	-0.351855000	-1.112661000
8	-1.359790000	-2.429613000	-0.269837000
6	-4.393711000	-0.149745000	0.770847000
6	-3.664925000	-0.735754000	1.945030000
6	-4.755038000	1.131684000	0.726946000
6	-4.701260000	-1.111854000	-0.337166000
1	1.004675000	-1.522912000	0.358844000
6	1.928754000	0.762852000	-1.213490000
6	-1.345626000	2.027109000	-0.849196000
8	-1.688971000	1.967635000	0.512256000
6	3.132817000	0.356720000	-0.406008000
6	3.625882000	1.184493000	0.600632000
6	4.702770000	0.788314000	1.380250000
6	5.301920000	-0.446365000	1.164794000

6	4.818280000	-1.279351000	0.165779000
6	3.740872000	-0.878619000	-0.613950000
1	0.601121000	0.976808000	0.471895000
1	-0.393250000	0.834510000	-2.366363000
1	-4.261145000	-1.531599000	2.398540000
1	-2.731762000	-1.199133000	1.614284000
1	-3.447041000	0.013276000	2.705738000
1	-5.282910000	1.538723000	-0.127142000
1	-2.494661000	1.435988000	0.590764000
1	-4.553978000	1.805495000	1.552510000
1	-3.787751000	-1.605354000	-0.674365000
1	-5.370916000	-1.894565000	0.029629000
1	-5.176275000	-0.615254000	-1.182044000
1	1.952492000	1.837804000	-1.411324000
1	1.926485000	0.243766000	-2.174160000
1	-2.228644000	2.183608000	-1.476121000
1	-0.675610000	2.878688000	-0.989912000
1	3.163136000	2.150367000	0.770845000
1	5.077121000	1.444935000	2.155043000
1	6.143603000	-0.755301000	1.770702000
1	5.282052000	-2.241275000	-0.010941000
1	3.367383000	-1.529491000	-1.396266000

$E = -864.646851$ a.u. (M06-2X/def2-TZVP)

$E = -864.683726$ a.u. (ωB97M-V/def2-TZVP)

$G_{vrt} = 0.288495$ a.u. (M06-2X/def2-TZVP)

P

Oxazolidin-2-one (IEFPCM, solvent=1,4-dioxane)

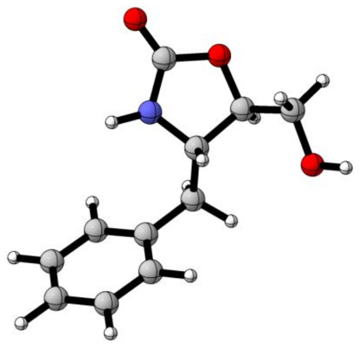

6	-0.772020000	0.184362000	0.049711000
6	-2.228348000	0.401116000	-0.401903000
8	-2.917805000	-0.803663000	-0.065972000
6	-2.030228000	-1.776106000	0.274597000
7	-0.775592000	-1.255038000	0.215727000
8	-2.365156000	-2.892148000	0.557867000

6	-2.908051000	1.562127000	0.287598000
6	0.264985000	0.667274000	-0.960117000
8	-2.153647000	2.711978000	-0.042506000
6	1.664254000	0.372553000	-0.490511000
6	2.301209000	1.226598000	0.408041000
6	3.569342000	0.933901000	0.888051000
6	4.220275000	-0.222325000	0.476032000
6	3.595839000	-1.080080000	-0.418502000
6	2.326206000	-0.782965000	-0.897884000
1	-0.596735000	0.688187000	1.007322000
1	-2.272186000	0.534560000	-1.486459000
1	-0.007886000	-1.764598000	0.621899000
1	-3.941798000	1.641322000	-0.059398000
1	-2.913702000	1.379143000	1.367566000
1	0.122151000	1.740968000	-1.095151000
1	0.073423000	0.174038000	-1.915334000
1	-2.563133000	3.484864000	0.355378000
1	1.796740000	2.131933000	0.727567000
1	4.052664000	1.610248000	1.581385000
1	5.210744000	-0.450472000	0.847508000
1	4.098328000	-1.980402000	-0.748087000
1	1.842637000	-1.452586000	-1.600089000

E = −707.437868 a.u. (M06-2X/def2-TZVP)

E = −707.48473 a.u. (ωB97M-V/def2-TZVP)

G_{vrt} = 0.189682 a.u. (M06-2X/def2-TZVP)

Isobutylene (IEFPCM, solvent=1,4-dioxane)

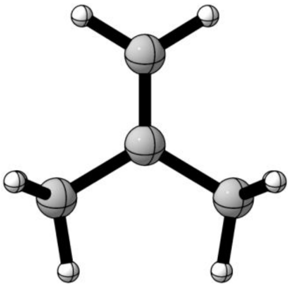

6	0.000000000	1.297814000	-0.670258000
6	0.000000000	0.000000000	0.106709000
6	0.000000000	-1.297814000	-0.670258000
6	0.000000000	0.000000000	1.434517000
1	0.000662000	1.125298000	-1.745087000
1	-0.879391000	1.896632000	-0.428813000
1	0.878687000	1.897255000	-0.427813000
1	-0.000662000	-1.125298000	-1.745087000

1	0.879391000	-1.896632000	-0.428813000
1	-0.878687000	-1.897255000	-0.427813000
1	-0.000046000	-0.924722000	1.999583000
1	0.000046000	0.924722000	1.999583000

$E = −157.189362$ a.u. (M06-2X/def2-TZVP)

$E = −157.179105$ a.u. (ωB97M-V/def2-TZVP)

G_{vrt} = 0.081953 a.u. (M06-2X/def2-TZVP)

PRC$_1$ (IEFPCM, solvent=acetonitrile)

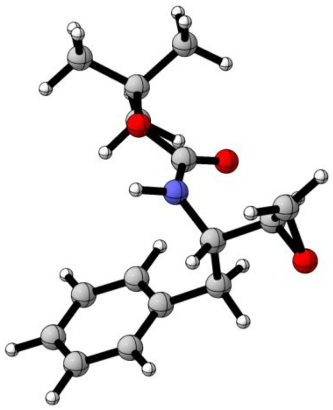

6	-0.983732000	0.705891000	0.026100000
7	0.115006000	0.887287000	0.797170000
6	1.399477000	1.310465000	0.259228000
6	1.351317000	2.750675000	-0.192011000
8	-1.070300000	1.053634000	-1.135277000
8	-1.949346000	0.104147000	0.733883000
6	-3.238599000	-0.220516000	0.133620000
6	-3.992638000	-0.869228000	1.284182000
6	-3.950635000	1.046874000	-0.317369000
6	-3.048877000	-1.214898000	-1.003052000
1	0.095996000	0.466722000	1.711500000
6	1.919869000	0.405511000	-0.871929000
6	1.686871000	3.832508000	0.731184000
8	2.614666000	3.393686000	-0.257846000
6	1.941648000	-1.043264000	-0.471383000
6	2.993125000	-1.559264000	0.283665000
6	2.986700000	-2.884450000	0.698413000
6	1.923105000	-3.714240000	0.364545000
6	0.869710000	-3.210790000	-0.388060000
6	0.881473000	-1.884772000	-0.802001000
1	2.101856000	1.254554000	1.093142000
1	0.692081000	2.959437000	-1.028223000

1	-4.986950000	-1.165827000	0.951060000
1	-3.461788000	-1.754663000	1.634966000
1	-4.095605000	-0.168772000	2.113404000
1	-4.972067000	0.794477000	-0.604862000
1	-3.449234000	1.504140000	-1.166284000
1	-3.993851000	1.763864000	0.503904000
1	-2.482934000	-2.079765000	-0.652117000
1	-4.026766000	-1.560044000	-1.340975000
1	-2.528196000	-0.761774000	-1.843404000
1	2.925846000	0.749761000	-1.119369000
1	1.289853000	0.543185000	-1.750478000
1	1.957555000	3.586670000	1.752094000
1	1.278371000	4.822339000	0.568257000
1	3.826610000	-0.916132000	0.544421000
1	3.813833000	-3.270996000	1.280221000
1	1.917625000	-4.747674000	0.686034000
1	0.038728000	-3.851409000	-0.655629000
1	0.060945000	-1.493845000	-1.394720000

E = −864.641503 a.u. (M06-2X/def2-TZVP)

E = −864.676300 a.u. (ωB97M-V/def2-TZVP)

G_{vrt} = 0.293311 a.u. (M06-2X/def2-TZVP)

TS$_1$ (IEFPCM, solvent=acetonitrile)

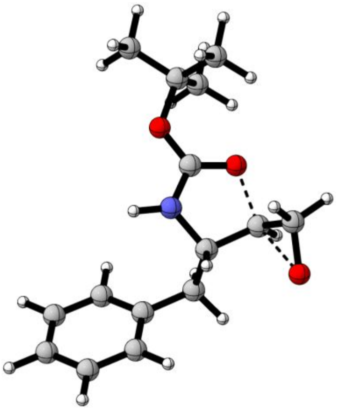

6	1.341376000	-0.027196000	-0.111717000
7	0.067853000	-0.027092000	-0.475562000
6	-0.673699000	1.164380000	-0.075344000
6	0.380844000	2.157918000	0.403599000
8	1.768811000	0.994820000	0.476286000
8	2.042755000	-1.086740000	-0.400204000
6	3.487590000	-1.184963000	-0.096898000
6	3.835420000	-2.565567000	-0.624113000

6	4.239413000	-0.105958000	-0.857457000
6	3.701092000	-1.107523000	1.405280000
1	-0.358034000	-0.815477000	-0.937827000
6	-1.715409000	0.843674000	0.998744000
6	0.662532000	3.297441000	-0.477334000
8	-0.563060000	3.820935000	-0.123889000
6	-2.739736000	-0.143527000	0.508215000
6	-3.798106000	0.282340000	-0.293797000
6	-4.722072000	-0.625995000	-0.789871000
6	-4.599093000	-1.978393000	-0.492205000
6	-3.548827000	-2.413728000	0.304189000
6	-2.626003000	-1.500449000	0.800485000
1	-1.162011000	1.593314000	-0.948737000
1	0.397328000	2.394118000	1.457443000
1	4.894525000	-2.758463000	-0.456972000
1	3.254437000	-3.328650000	-0.106346000
1	3.632034000	-2.628234000	-1.692976000
1	5.309060000	-0.284970000	-0.746217000
1	4.013084000	0.886837000	-0.475552000
1	3.992996000	-0.150946000	-1.918808000
1	3.074179000	-1.840725000	1.913793000
1	4.744288000	-1.341909000	1.618889000
1	3.482817000	-0.115125000	1.792096000
1	-2.190167000	1.786787000	1.273123000
1	-1.199662000	0.453159000	1.879163000
1	0.774354000	3.010402000	-1.535717000
1	1.544979000	3.879680000	-0.177348000
1	-3.898673000	1.337088000	-0.524846000
1	-5.541069000	-0.278715000	-1.406652000
1	-5.320687000	-2.687442000	-0.876629000
1	-3.448273000	-3.464570000	0.543690000
1	-1.810454000	-1.843354000	1.427158000

$E = -864.577580$ a.u. (M06-2X/def2-TZVP)

$E = -864.619510$ a.u. (ωB97M-V/def2-TZVP)

$G_{vrt} = 0.292012$ a.u. (M06-2X/def2-TZVP)

$f = 613.2028i$ (M06-2X/def2-TZVP)

INT (IEFPCM, solvent=acetonitrile)

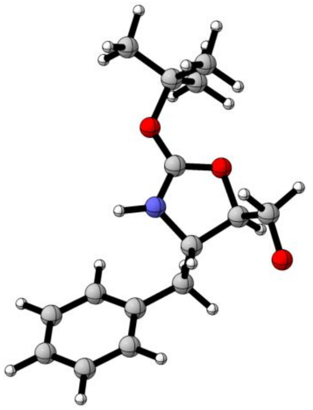

6	1.279856000	-0.165868000	-0.111183000
7	0.018320000	-0.144119000	-0.458648000
6	-0.649546000	1.066073000	0.031048000
6	0.560214000	1.870678000	0.534874000
8	1.669925000	0.884520000	0.535630000
8	2.045442000	-1.152838000	-0.390045000
6	3.518914000	-1.160631000	-0.114772000
6	3.932160000	-2.507150000	-0.676045000
6	4.167134000	-0.019402000	-0.875998000
6	3.745783000	-1.092404000	1.383631000
1	-0.424940000	-0.898247000	-0.962507000
6	-1.698080000	0.747661000	1.093272000
6	0.880815000	3.052295000	-0.380211000
8	-0.169820000	3.897476000	-0.385185000
6	-2.806217000	-0.110388000	0.544285000
6	-3.825277000	0.460383000	-0.217445000
6	-4.822856000	-0.329111000	-0.770593000
6	-4.813894000	-1.705075000	-0.571608000
6	-3.803637000	-2.283608000	0.183940000
6	-2.806411000	-1.489351000	0.738126000
1	-1.103511000	1.604887000	-0.800058000
1	0.447107000	2.192528000	1.567915000
1	5.004717000	-2.635100000	-0.535812000
1	3.412410000	-3.312705000	-0.158234000
1	3.708450000	-2.562775000	-1.740870000
1	5.248642000	-0.128815000	-0.795092000
1	3.890426000	0.951279000	-0.470386000
1	3.896151000	-0.064262000	-1.930999000
1	3.172941000	-1.869246000	1.890166000
1	4.804553000	-1.268743000	1.574052000
1	3.481544000	-0.120108000	1.792171000
1	-2.090921000	1.704251000	1.444315000
1	-1.211360000	0.254469000	1.937826000
1	1.132236000	2.600145000	-1.372922000
1	1.839841000	3.479550000	-0.012705000
1	-3.836493000	1.533578000	-0.372116000

1	-5.610296000	0.129160000	-1.355053000
1	-5.593194000	-2.321284000	-1.000753000
1	-3.791967000	-3.353658000	0.346746000
1	-2.022702000	-1.943939000	1.333723000

E = −864.591804 a.u. (M06-2X/def2-TZVP)

E = −864.630418 a.u. (ωB97M-V/def2-TZVP)

G_{vrt} = 0.294043 a.u. (M06-2X/def2-TZVP)

PRC$_2$ (IEFPCM, solvent=acetonitrile)

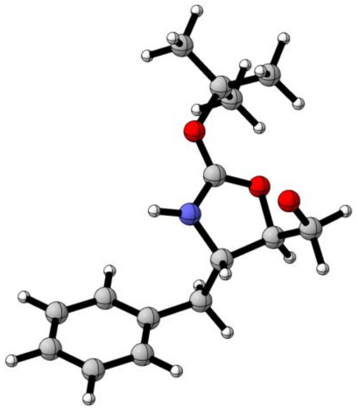

6	1.199449000	-0.106002000	-0.054005000
7	-0.063595000	-0.033765000	-0.388674000
6	-0.685013000	1.162843000	0.175226000
6	0.548858000	1.877127000	0.759522000
8	1.592166000	0.828296000	0.747352000
8	1.951505000	-1.063033000	-0.453824000
6	3.396972000	-1.186919000	-0.091215000
6	3.802234000	-2.435043000	-0.851579000
6	4.149511000	0.032371000	-0.590552000
6	3.512226000	-1.389963000	1.407991000
1	-0.501299000	-0.679712000	-1.027834000
6	-1.765570000	0.814325000	1.196617000
6	1.042934000	3.004044000	-0.148887000
8	1.234155000	2.587434000	-1.414645000
6	-2.888649000	0.029064000	0.573297000
6	-3.886402000	0.678780000	-0.152183000
6	-4.896389000	-0.043161000	-0.771692000
6	-4.921135000	-1.429820000	-0.676630000
6	-3.931927000	-2.086902000	0.042049000
6	-2.922684000	-1.360304000	0.662697000
1	-1.089343000	1.764045000	-0.639917000
1	0.424282000	2.161437000	1.803191000

1	4.853058000	-2.644776000	-0.656170000
1	3.208146000	-3.289334000	-0.528344000
1	3.666918000	-2.289985000	-1.922999000
1	3.904630000	0.924293000	-0.018923000
1	3.931660000	0.208433000	-1.644077000
1	5.216979000	-0.163660000	-0.487268000
1	2.890539000	-2.226445000	1.727598000
1	4.550653000	-1.625488000	1.641625000
1	3.231853000	-0.496179000	1.960599000
1	-2.140887000	1.753293000	1.609048000
1	-1.312000000	0.248152000	2.013194000
1	1.947284000	3.413719000	0.355600000
1	0.262754000	3.794121000	-0.019037000
1	-3.871991000	1.760411000	-0.226762000
1	-5.666760000	0.476319000	-1.327023000
1	-5.709513000	-1.993667000	-1.158023000
1	-3.946037000	-3.166178000	0.123600000
1	-2.154848000	-1.876655000	1.227631000

$E = -864.595064$ a.u. (M06-2X/def2-TZVP)

$E = -864.633962$ a.u. (ωB97M-V/def2-TZVP)

$G_{vrt} = 0.294738$ a.u. (M06-2X/def2-TZVP)

TS$_{1,2}$' (IEFPCM, solvent=acetonitrile)

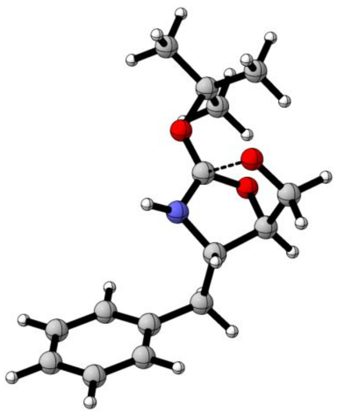

6	1.188314000	-0.047549000	-0.066258000
7	-0.106803000	-0.059980000	-0.381634000
6	-0.711235000	1.172709000	0.144099000
6	0.539156000	1.871597000	0.711910000
8	1.449269000	0.747014000	0.938384000
8	1.978141000	-0.972205000	-0.472044000
6	3.402423000	-1.069835000	-0.053075000
6	3.901333000	-2.231488000	-0.892458000
6	4.136725000	0.212179000	-0.397278000

6	3.455498000	-1.404489000	1.427755000
1	-0.393155000	-0.451143000	-1.266038000
6	-1.802977000	0.873687000	1.169236000
6	1.260818000	2.666461000	-0.384873000
8	1.569620000	1.809553000	-1.391028000
6	-2.942472000	0.101646000	0.560647000
6	-3.944915000	0.764736000	-0.145842000
6	-4.973424000	0.057217000	-0.752299000
6	-5.012762000	-1.329126000	-0.661838000
6	-4.019147000	-1.999853000	0.038805000
6	-2.991523000	-1.287583000	0.644982000
1	-1.109726000	1.762191000	-0.683015000
1	0.386125000	2.374262000	1.662965000
1	4.952096000	-2.410360000	-0.667429000
1	3.336318000	-3.136243000	-0.668611000
1	3.804007000	-2.003488000	-1.953536000
1	5.193382000	0.060806000	-0.172436000
1	3.766840000	1.056940000	0.178794000
1	4.024864000	0.441597000	-1.454954000
1	2.835073000	-2.274889000	1.642993000
1	4.486316000	-1.645023000	1.689007000
1	3.131393000	-0.568128000	2.042569000
1	-2.161164000	1.827123000	1.563299000
1	-1.361932000	0.313511000	1.996706000
1	2.138081000	3.146826000	0.096352000
1	0.573029000	3.490151000	-0.666963000
1	-3.919881000	1.846590000	-0.216124000
1	-5.746668000	0.587918000	-1.292976000
1	-5.815315000	-1.882328000	-1.132158000
1	-4.044333000	-3.079320000	0.116314000
1	-2.218829000	-1.813881000	1.193103000

$E = -864.592331$ a.u. (M06-2X/def2-TZVP)

$E = -864.633101$ a.u. (ωB97M-V/def2-TZVP)

$G_{vrt} = 0.295947$ a.u. (M06-2X/def2-TZVP)

$f = 233.7753i$ (M06-2X/def2-TZVP)

INT’ (IEFPCM, solvent=acetonitrile)

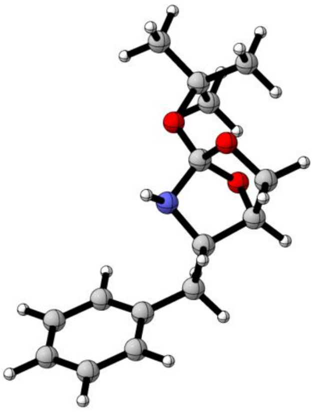

6	-1.304128000	-0.272337000	-0.340095000
7	0.113129000	-0.078293000	-0.595800000
6	0.744984000	-1.251104000	0.061065000
6	-0.501843000	-1.939406000	0.639923000
8	-1.316266000	-0.804734000	0.963301000
8	-1.995478000	0.865028000	-0.535282000
6	-3.339225000	1.056154000	-0.000817000
6	-3.884410000	2.198791000	-0.843724000
6	-4.222644000	-0.175084000	-0.146061000
6	-3.226374000	1.481846000	1.458184000
1	0.283072000	-0.065826000	-1.594441000
6	1.743487000	-0.826009000	1.137099000
6	-1.325721000	-2.531159000	-0.507219000
8	-1.805359000	-1.343080000	-1.146741000
6	2.912370000	-0.078105000	0.556641000
6	3.962314000	-0.771631000	-0.044305000
6	5.028021000	-0.093355000	-0.619657000
6	5.059707000	1.296061000	-0.602176000
6	4.020374000	1.997638000	-0.005530000
6	2.955569000	1.313618000	0.568327000
1	1.242183000	-1.895029000	-0.666907000
1	-0.347865000	-2.575351000	1.505385000
1	-4.880742000	2.473398000	-0.497352000
1	-3.234149000	3.070466000	-0.763391000
1	-3.946100000	1.901189000	-1.890819000
1	-5.221637000	0.083766000	0.208049000
1	-3.850474000	-1.007537000	0.448901000
1	-4.288990000	-0.487455000	-1.186908000
1	-2.552862000	2.335137000	1.546894000
1	-4.209730000	1.776989000	1.826966000
1	-2.851413000	0.668448000	2.075906000
1	2.096655000	-1.724025000	1.650408000
1	1.218660000	-0.204294000	1.864776000
1	-2.171125000	-3.115665000	-0.141052000
1	-0.731194000	-3.125454000	-1.200377000
1	3.944845000	-1.856102000	-0.056236000

1	5.836266000	-0.648826000	-1.078267000
1	5.890826000	1.827332000	-1.047701000
1	4.038257000	3.079998000	0.014657000
1	2.145550000	1.863685000	1.031960000

$E = -864.629534$ a.u. (M06-2X/def2-TZVP)

$E = -864,670398$ a.u. (ωB97M-V/def2-TZVP)

$G_{vrt} = 0.299903$ a.u. (M06-2X/def2-TZVP)

TS$_2$ (IEFPCM, solvent=acetonitrile)

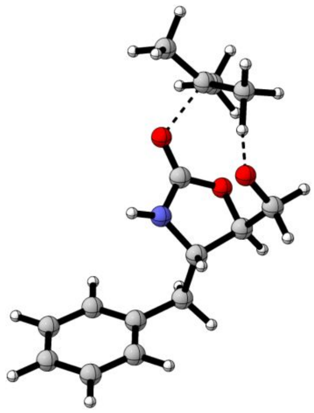

6	1.086570000	-0.397636000	0.191602000
7	-0.168360000	-0.262888000	-0.236888000
6	-0.709480000	1.040016000	0.139399000
6	0.547960000	1.700004000	0.735460000
8	1.405003000	0.565221000	1.044706000
8	1.868196000	-1.313274000	-0.099212000
6	3.753908000	-0.897751000	-0.141040000
6	4.061446000	-2.044680000	-1.049101000
6	3.899426000	0.436032000	-0.672189000
6	4.028032000	-1.126053000	1.310356000
1	-0.503456000	-0.803768000	-1.017460000
6	-1.878171000	0.919278000	1.117677000
6	1.325315000	2.526935000	-0.287995000
8	1.681610000	1.759190000	-1.365850000
6	-3.037512000	0.172442000	0.515846000
6	-3.954908000	0.831929000	-0.300890000
6	-4.999632000	0.140505000	-0.898185000
6	-5.140794000	-1.226088000	-0.687916000
6	-4.232235000	-1.893390000	0.122708000
6	-3.188222000	-1.197195000	0.719441000
1	-1.017022000	1.578387000	-0.758665000
1	0.351256000	2.225821000	1.667888000

1	5.150972000	-2.108515000	-1.118849000
1	3.682347000	-2.985437000	-0.656819000
1	3.667190000	-1.868511000	-2.048210000
1	4.330526000	1.122816000	0.055766000
1	2.838308000	0.985048000	-0.990349000
1	4.453972000	0.431961000	-1.610358000
1	3.648473000	-2.087816000	1.648407000
1	5.116424000	-1.122273000	1.424988000
1	3.620489000	-0.322420000	1.919023000
1	-2.184377000	1.929141000	1.399019000
1	-1.525920000	0.412580000	2.019217000
1	2.195167000	2.952992000	0.247771000
1	0.678943000	3.381074000	-0.559136000
1	-3.850751000	1.898808000	-0.464707000
1	-5.706473000	0.668698000	-1.525272000
1	-5.956510000	-1.766196000	-1.150735000
1	-4.337096000	-2.957138000	0.294021000
1	-2.482635000	-1.720220000	1.354476000

E = −864.569718 a.u. (M06-2X/def2-TZVP)

E = −864.611844 a.u. (ωB97M-V/def2-TZVP)

G_{vrt} = 0.289938 a.u. (M06-2X/def2-TZVP)

f = 871.6901i (M06-2X/def2-TZVP)

FC (IEFPCM, solvent=acetonitrile)

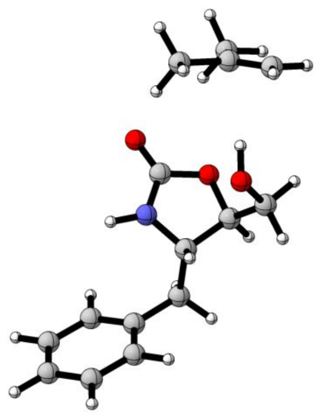

6	-0.788501000	-1.150402000	-0.566438000
7	0.346822000	-0.688255000	-0.009451000
6	0.669978000	0.670477000	-0.383674000
6	-0.596990000	1.054001000	-1.181293000
8	-1.363044000	-0.158307000	-1.286632000
8	-1.275791000	-2.253256000	-0.481998000
6	-4.452134000	-0.306325000	0.668791000
6	-3.807080000	-1.042959000	1.807420000

6	-4.845366000	0.962373000	0.775584000
6	-4.630358000	-1.099477000	-0.591577000
1	0.949737000	-1.290465000	0.526545000
6	1.954321000	0.771747000	-1.209406000
6	-1.437249000	2.110505000	-0.495298000
8	-1.756803000	1.731988000	0.825621000
6	3.154418000	0.303012000	-0.431898000
6	3.775073000	1.147884000	0.487554000
6	4.850942000	0.705331000	1.243783000
6	5.321786000	-0.593911000	1.092795000
6	4.711103000	-1.443961000	0.181059000
6	3.634508000	-0.996501000	-0.575761000
1	0.756457000	1.295903000	0.507687000
1	-0.346045000	1.375397000	-2.193309000
1	-4.399138000	-1.923005000	2.070717000
1	-2.821152000	-1.408921000	1.507353000
1	-3.701656000	-0.414984000	2.691396000
1	-5.306597000	1.480627000	-0.056705000
1	-2.578296000	1.221077000	0.798083000
1	-4.732413000	1.512612000	1.703182000
1	-3.670330000	-1.503691000	-0.919326000
1	-5.289745000	-1.951079000	-0.403157000
1	-5.058689000	-0.496153000	-1.390728000
1	2.078892000	1.815979000	-1.505852000
1	1.833150000	0.176786000	-2.117499000
1	-2.341053000	2.294164000	-1.082918000
1	-0.861121000	3.037341000	-0.454706000
1	3.413373000	2.163227000	0.606157000
1	5.324990000	1.375382000	1.949575000
1	6.162298000	-0.939345000	1.680540000
1	5.073992000	-2.455920000	0.054635000
1	3.164001000	-1.661304000	-1.291298000

E = −864.654965 a.u. (M06-2X/def2-TZVP)

E = −864,694462 a.u. (ωB97M-V/def2-TZVP)

G_{vrt} = 0.287523 a.u. (M06-2X/def2-TZVP)

P

Oxazolidin-2-one (IEFPCM, solvent=acetonitrile)

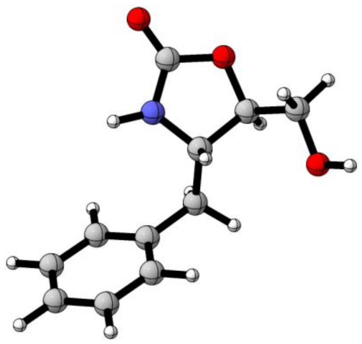

6	-0.771798000	0.191447000	0.053606000
6	-2.225828000	0.408503000	-0.409053000
8	-2.914225000	-0.808653000	-0.096293000
6	-2.030363000	-1.765986000	0.277592000
7	-0.784591000	-1.243350000	0.265878000
8	-2.374809000	-2.888878000	0.551613000
6	-2.916540000	1.557097000	0.290362000
6	0.268089000	0.634830000	-0.971498000
8	-2.176607000	2.715978000	-0.040390000
6	1.668329000	0.355095000	-0.495058000
6	2.302932000	1.232250000	0.383637000
6	3.574678000	0.957214000	0.865712000
6	4.231670000	-0.204217000	0.475830000
6	3.609084000	-1.084969000	-0.398151000
6	2.335581000	-0.805407000	-0.879696000
1	-0.593150000	0.718740000	0.996495000
1	-2.264011000	0.552158000	-1.491720000
1	-0.018001000	-1.751640000	0.676479000
1	-3.952473000	1.628043000	-0.051346000
1	-2.913675000	1.372118000	1.369489000
1	0.127797000	1.704112000	-1.141041000
1	0.074315000	0.112828000	-1.910694000
1	-2.563205000	3.473971000	0.408140000
1	1.794465000	2.140890000	0.686753000
1	4.055709000	1.650835000	1.543468000
1	5.224674000	-0.418540000	0.849031000
1	4.115390000	-1.989710000	-0.709456000
1	1.854437000	-1.493081000	-1.565887000

E = −707.446958 a.u. (M06-2X/def2-TZVP)

E = −707.495026 a.u. (ωB97M-V/def2-TZVP)

G_{vrt} = 0.189128 a.u. (M06-2X/def2-TZVP)

Isobutylene (IEFPCM, solvent=acetonitrile)

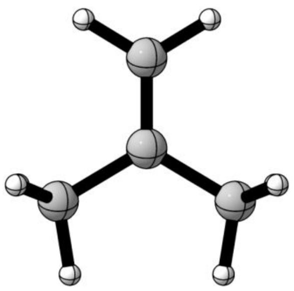

6	0.000000000	1.297709000	-0.670745000
6	0.000000000	0.000000000	0.106723000
6	0.000000000	-1.297709000	-0.670745000
6	0.000000000	0.000000000	1.435505000
1	0.000671000	1.124030000	-1.745231000
1	-0.879049000	1.897028000	-0.429085000
1	0.878330000	1.897664000	-0.428068000
1	-0.000671000	-1.124030000	-1.745231000
1	0.879049000	-1.897028000	-0.429085000
1	-0.878330000	-1.897664000	-0.428068000
1	-0.000048000	-0.925279000	2.000168000
1	0.000048000	0.925279000	2.000168000

E = −157.190132 a.u. (M06-2X/def2-TZVP)

E = −157.181382 a.u. (ωB97M-V/def2-TZVP)

G_{vrt} = 0.081801 a.u. (M06-2X/def2-TZVP)

PRC$_1$ (IEFPCM, solvent=water)

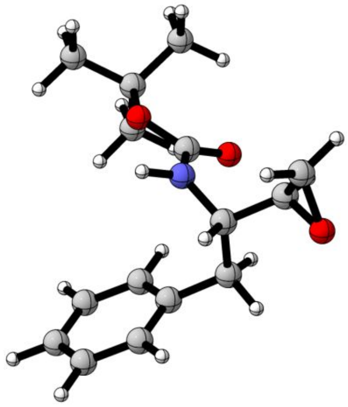

6	0.979894000	0.709523000	-0.026092000
7	-0.119094000	0.888361000	-0.797143000
6	-1.405569000	1.305778000	-0.259439000

6	-1.363764000	2.746223000	0.191638000
8	1.064820000	1.055840000	1.136030000
8	1.947671000	0.111843000	-0.734137000
6	3.238572000	-0.208376000	-0.134493000
6	3.994532000	-0.853365000	-1.285861000
6	3.945661000	1.061566000	0.316975000
6	3.053243000	-1.204423000	1.001398000
1	-0.097648000	0.471692000	-1.713222000
6	-1.922123000	0.398497000	0.871573000
6	-1.706195000	3.826415000	-0.730980000
8	-2.630132000	3.383111000	0.259981000
6	-1.936127000	-1.050551000	0.471491000
6	-2.982846000	-1.571339000	-0.286900000
6	-2.969379000	-2.896673000	-0.701079000
6	-1.903370000	-3.721812000	-0.363310000
6	-0.854621000	-3.213545000	0.392584000
6	-0.873440000	-1.887381000	0.805944000
1	-2.107505000	1.246586000	-1.093462000
1	-0.704038000	2.958421000	1.026613000
1	4.989951000	-1.146812000	-0.953261000
1	3.466719000	-1.740430000	-1.637131000
1	4.094828000	-0.151873000	-2.114528000
1	4.967864000	0.813007000	0.605031000
1	3.442089000	1.516797000	1.165716000
1	3.986598000	1.778818000	-0.504179000
1	2.490392000	-2.071159000	0.650116000
1	4.032684000	-1.546099000	1.338350000
1	2.531334000	-0.754143000	1.842488000
1	-2.930112000	0.737571000	1.117991000
1	-1.293672000	0.539574000	1.750672000
1	-1.977830000	3.579077000	-1.751240000
1	-1.302227000	4.818203000	-0.568804000
1	-3.818105000	-0.931874000	-0.550914000
1	-3.792871000	-3.286900000	-1.285588000
1	-1.892367000	-4.755329000	-0.684392000
1	-0.021748000	-3.850455000	0.663104000
1	-0.056358000	-1.492772000	1.400958000

E = −864.641805 a.u. (M06-2X/def2-TZVP)

E = −864.668315 a.u. (ωB97M-V/def2-TZVP)

G_{vrt} = 0.293266 a.u. (M06-2X/def2-TZVP)

TS_1 (IEFPCM, solvent=water)

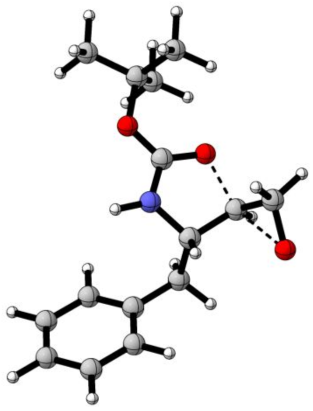

6	1.340321000	-0.024400000	-0.114413000
7	0.068106000	-0.020383000	-0.483487000
6	-0.674279000	1.168165000	-0.076991000
6	0.379338000	2.161879000	0.403025000
8	1.767314000	0.993971000	0.479870000
8	2.040601000	-1.083950000	-0.405556000
6	3.483600000	-1.187587000	-0.095611000
6	3.830345000	-2.566509000	-0.627937000
6	4.242211000	-0.106928000	-0.847027000
6	3.689754000	-1.118140000	1.407988000
1	-0.357459000	-0.806592000	-0.949762000
6	-1.712567000	0.842288000	0.999028000
6	0.667268000	3.298683000	-0.478709000
8	-0.557604000	3.825774000	-0.125652000
6	-2.737064000	-0.144785000	0.508601000
6	-3.800357000	0.282352000	-0.286186000
6	-4.724838000	-0.625785000	-0.781771000
6	-4.597352000	-1.979242000	-0.490838000
6	-3.542027000	-2.415840000	0.298223000
6	-2.618757000	-1.502774000	0.794081000
1	-1.165310000	1.599206000	-0.947808000
1	0.395921000	2.398148000	1.456782000
1	4.888106000	-2.763082000	-0.456642000
1	3.244779000	-3.330537000	-0.116768000
1	3.631983000	-2.623290000	-1.698080000
1	5.310784000	-0.289207000	-0.730832000
1	4.016278000	0.884607000	-0.461611000
1	4.001352000	-0.146273000	-1.909873000
1	3.058062000	-1.851909000	1.909713000
1	4.731173000	-1.356602000	1.625660000
1	3.472380000	-0.126995000	1.798534000
1	-2.188220000	1.783559000	1.278365000
1	-1.193568000	0.449604000	1.876557000
1	0.778284000	3.010927000	-1.536743000
1	1.550877000	3.878605000	-0.178228000
1	-3.904482000	1.337879000	-0.512077000
1	-5.547654000	-0.277573000	-1.392926000

1	-5.319250000	-2.688177000	-0.874910000
1	-3.437775000	-3.467552000	0.532238000
1	-1.799076000	-1.846692000	1.414758000

E = −864.578214 a.u. (M06-2X/def2-TZVP)

E = −864.619603 a.u. (ωB97M-V/def2-TZVP)

G_{vrt} = 0.291883 a.u. (M06-2X/def2-TZVP)

f = 616.4581i (M06-2X/def2-TZVP)

INT (IEFPCM, solvent=water)

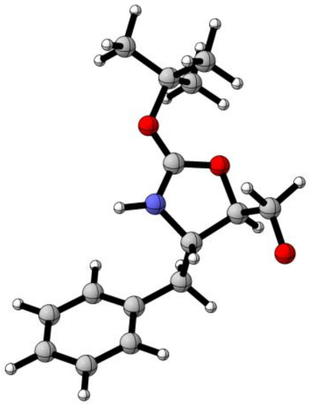

6	1.280517000	-0.166336000	-0.112704000
7	0.018961000	-0.145154000	-0.460031000
6	-0.649289000	1.064734000	0.029049000
6	0.560217000	1.869894000	0.533240000
8	1.670383000	0.885207000	0.532951000
8	2.046300000	-1.153083000	-0.390292000
6	3.519618000	-1.161374000	-0.113921000
6	3.932331000	-2.508967000	-0.672913000
6	4.168893000	-0.021593000	-0.876396000
6	3.745323000	-1.090949000	1.384537000
1	-0.423890000	-0.899752000	-0.963641000
6	-1.697828000	0.746791000	1.091625000
6	0.881972000	3.052543000	-0.379893000
8	-0.165957000	3.902452000	-0.381629000
6	-2.806931000	-0.110518000	0.543362000
6	-3.827399000	0.460999000	-0.215958000
6	-4.826184000	-0.327875000	-0.767917000
6	-4.816936000	-1.704034000	-0.570062000
6	-3.805239000	-2.283279000	0.183100000
6	-2.806850000	-1.489644000	0.736108000
1	-1.103918000	1.601934000	-0.802843000
1	0.446947000	2.190727000	1.566641000
1	5.004656000	-2.637520000	-0.531516000

1	3.411417000	-3.313278000	-0.154357000
1	3.709461000	-2.565857000	-1.737853000
1	5.250291000	-0.131060000	-0.794121000
1	3.891896000	0.949757000	-0.472518000
1	3.898914000	-0.068130000	-1.931577000
1	3.171437000	-1.866514000	1.891792000
1	4.803845000	-1.267616000	1.576002000
1	3.481316000	-0.117842000	1.791316000
1	-2.090198000	1.703448000	1.443124000
1	-1.211031000	0.253296000	1.935861000
1	1.129708000	2.602349000	-1.373964000
1	1.842836000	3.476088000	-0.013321000
1	-3.838970000	1.534351000	-0.369687000
1	-5.614678000	0.130924000	-1.350560000
1	-5.597011000	-2.319841000	-0.998389000
1	-3.793098000	-3.353500000	0.344702000
1	-2.021756000	-1.944847000	1.329350000

$E = -864.592926$ a.u. (M06-2X/def2-TZVP)

$E = -864.638672$ a.u. (ωB97M-V/def2-TZVP)

$G_{vrt} = 0.294068$ a.u. (M06-2X/def2-TZVP)

PRC$_2$ (IEFPCM, solvent=water)

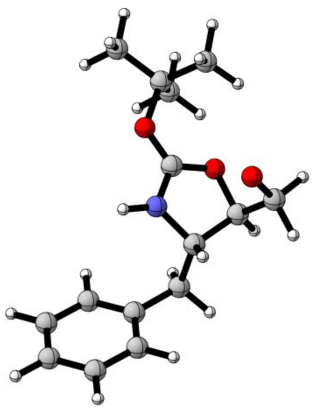

6	1.200662000	-0.105324000	-0.056992000
7	-0.062203000	-0.033345000	-0.392048000
6	-0.685020000	1.161728000	0.173460000
6	0.548271000	1.877440000	0.757307000
8	1.594007000	0.831358000	0.741357000
8	1.953070000	-1.062343000	-0.455787000
6	3.397714000	-1.187949000	-0.089511000
6	3.802612000	-2.437990000	-0.846836000
6	4.153222000	0.029285000	-0.589604000
6	3.509260000	-1.388357000	1.410244000
1	-0.502253000	-0.685286000	-1.023575000

6	-1.763814000	0.811071000	1.196091000
6	1.038408000	3.009011000	-0.146835000
8	1.230458000	2.598881000	-1.415443000
6	-2.887672000	0.026402000	0.573447000
6	-3.890672000	0.677540000	-0.143496000
6	-4.901612000	-0.043653000	-0.762414000
6	-4.921971000	-1.430933000	-0.675371000
6	-3.927525000	-2.089403000	0.034838000
6	-2.917441000	-1.363598000	0.655078000
1	-1.091772000	1.762914000	-0.640483000
1	0.424145000	2.158665000	1.801853000
1	4.852620000	-2.649082000	-0.648600000
1	3.206315000	-3.290646000	-0.523408000
1	3.670013000	-2.294576000	-1.918827000
1	5.220158000	-0.168401000	-0.483985000
1	3.909407000	0.922520000	-0.019539000
1	3.938074000	0.203273000	-1.644060000
1	2.885523000	-2.223270000	1.729880000
1	4.546841000	-1.625085000	1.646359000
1	3.229039000	-0.493193000	1.960692000
1	-2.138755000	1.749357000	1.610320000
1	-1.308608000	0.243907000	2.011030000
1	1.941969000	3.419084000	0.358350000
1	0.256470000	3.796410000	-0.013760000
1	-3.879657000	1.759631000	-0.211647000
1	-5.676126000	0.476851000	-1.310995000
1	-5.711017000	-1.994222000	-1.156332000
1	-3.938185000	-3.169171000	0.110113000
1	-2.145590000	-1.881054000	1.213501000

E = −864.596081 a.u. (M06-2X/def2-TZVP)

E = −864.641035 a.u. (ωB97M-V/def2-TZVP)

G_{vrt} = 0.294726 a.u. (M06-2X/def2-TZVP)

$TS_{1.2}$' (IEFPCM, solvent=water)

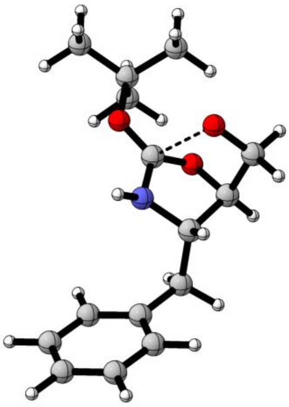

6	1.188632000	-0.043555000	-0.070409000
7	-0.106873000	-0.056710000	-0.385601000
6	-0.711738000	1.175173000	0.141646000
6	0.538848000	1.873699000	0.709519000
8	1.447823000	0.748814000	0.936979000
8	1.978323000	-0.968861000	-0.475553000
6	3.400535000	-1.071046000	-0.050919000
6	3.900177000	-2.231921000	-0.890937000
6	4.139773000	0.209850000	-0.388725000
6	3.446552000	-1.409310000	1.429314000
1	-0.393583000	-0.447587000	-1.270002000
6	-1.802079000	0.874468000	1.167859000
6	1.262602000	2.666714000	-0.387104000
8	1.575127000	1.806513000	-1.390340000
6	-2.941547000	0.101596000	0.560311000
6	-3.947966000	0.764457000	-0.140742000
6	-4.976779000	0.056299000	-0.746055000
6	-5.012368000	-1.330451000	-0.659945000
6	-4.014741000	-2.000961000	0.035281000
6	-2.986910000	-1.288068000	0.640419000
1	-1.111723000	1.765488000	-0.684187000
1	0.385521000	2.377627000	1.659783000
1	4.949492000	-2.413993000	-0.661800000
1	3.331913000	-3.135747000	-0.671658000
1	3.808068000	-2.001185000	-1.951916000
1	5.194755000	0.055651000	-0.158083000
1	3.768527000	1.054286000	0.186946000
1	4.034974000	0.441077000	-1.446767000
1	2.822822000	-2.278491000	1.639811000
1	4.475616000	-1.653266000	1.694250000
1	3.122118000	-0.573533000	2.044749000
1	-2.160716000	1.827294000	1.562884000
1	-1.359285000	0.314194000	1.994317000
1	2.138417000	3.148684000	0.094682000
1	0.575323000	3.488749000	-0.674105000
1	-3.925802000	1.846583000	-0.207511000

1	-5.753169000	0.586802000	-1.282412000
1	-5.815109000	-1.884153000	-1.129369000
1	-4.036986000	-3.080735000	0.109404000
1	-2.211190000	-1.814171000	1.184431000

$E = -864.592939$ a.u. (M06-2X/def2-TZVP)

$E = -864.633321$ a.u. (ωB97M-V/def2-TZVP)

$G_{vrt} = 0.295939$ a.u. (M06-2X/def2-TZVP)

$f = 239.8391i$ (M06-2X/def2-TZVP)

INT’ (IEFPCM, solvent=water)

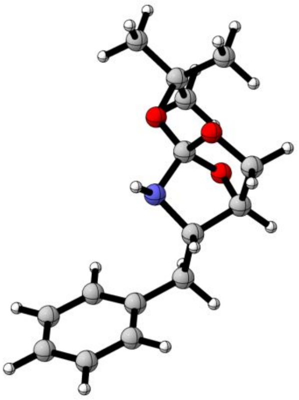

6	-1.304529000	-0.273774000	-0.340223000
7	0.112999000	-0.081471000	-0.597522000
6	0.744333000	-1.252312000	0.064066000
6	-0.502731000	-1.937752000	0.645633000
8	-1.316261000	-0.801043000	0.965096000
8	-1.995180000	0.863624000	-0.539102000
6	-3.338488000	1.057948000	-0.004044000
6	-3.883275000	2.198428000	-0.850091000
6	-4.223065000	-0.172955000	-0.144564000
6	-3.224045000	1.488258000	1.453455000
1	0.281803000	-0.075258000	-1.596490000
6	1.743046000	-0.824164000	1.138576000
6	-1.327333000	-2.533106000	-0.499054000
8	-1.806707000	-1.346748000	-1.142609000
6	2.912043000	-0.077710000	0.556386000
6	3.955197000	-0.772268000	-0.055277000
6	5.021252000	-0.095277000	-0.631487000
6	5.060330000	1.293866000	-0.604124000
6	4.027987000	1.996446000	0.003375000
6	2.962751000	1.313627000	0.578072000
1	1.240917000	-1.899220000	-0.661573000
1	-0.348970000	-2.570748000	1.513237000
1	-4.879126000	2.474794000	-0.503780000

1	-3.232454000	3.069982000	-0.772754000
1	-3.946117000	1.897760000	-1.896256000
1	-5.221295000	0.087739000	0.210287000
1	-3.850693000	-1.004088000	0.452096000
1	-4.291380000	-0.488081000	-1.184474000
1	-2.549512000	2.341101000	1.539041000
1	-4.206781000	1.785953000	1.821775000
1	-2.849993000	0.676370000	2.073707000
1	2.096123000	-1.721070000	1.653854000
1	1.218819000	-0.200652000	1.865165000
1	-2.172936000	-3.115743000	-0.130543000
1	-0.733325000	-3.129991000	-1.190333000
1	3.932099000	-1.856503000	-0.074990000
1	5.824061000	-0.651565000	-1.098575000
1	5.891761000	1.824032000	-1.050374000
1	4.051683000	3.078513000	0.031508000
1	2.158674000	1.864774000	1.050835000

$E = -864.629934$ a.u. (M06-2X/def2-TZVP)

$E = -864.663492$ a.u. (ωB97M-V/def2-TZVP)

$G_{vrt} = 0.299922$ a.u. (M06-2X/def2-TZVP)

TS$_2$ (IEFPCM, solvent=water)

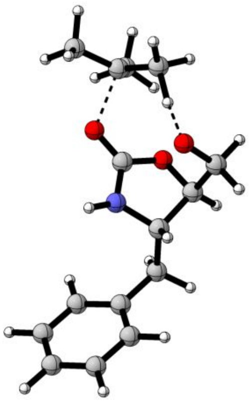

6	1.085895000	-0.399690000	0.191693000
7	-0.168890000	-0.264392000	-0.236923000
6	-0.710556000	1.038058000	0.139666000
6	0.547011000	1.698492000	0.734921000
8	1.404592000	0.564257000	1.043942000
8	1.867640000	-1.315057000	-0.098235000
6	3.760336000	-0.895591000	-0.140869000
6	4.066740000	-2.042923000	-1.047949000
6	3.901782000	0.437999000	-0.673039000
6	4.031542000	-1.122167000	1.310788000
1	-0.506464000	-0.808292000	-1.014297000

6	-1.878667000	0.916747000	1.118604000
6	1.323347000	2.526384000	-0.288496000
8	1.680402000	1.760175000	-1.367439000
6	-3.038659000	0.171244000	0.516349000
6	-3.957493000	0.832858000	-0.297097000
6	-5.002861000	0.142842000	-0.894978000
6	-5.143223000	-1.224474000	-0.688652000
6	-4.233245000	-1.893885000	0.118694000
6	-3.188622000	-1.199089000	0.716076000
1	-1.019148000	1.576550000	-0.757977000
1	0.350464000	2.224495000	1.667255000
1	5.156354000	-2.105805000	-1.118581000
1	3.689096000	-2.983551000	-0.654123000
1	3.671785000	-1.867976000	-2.046985000
1	4.331959000	1.126144000	0.054202000
1	2.840977000	0.982753000	-0.989440000
1	4.455744000	0.434297000	-1.611620000
1	3.653458000	-2.084434000	1.648846000
1	5.119873000	-1.116340000	1.426812000
1	3.621933000	-0.318603000	1.918095000
1	-2.184249000	1.926400000	1.401269000
1	-1.526066000	0.408712000	2.019243000
1	2.192963000	2.952777000	0.247440000
1	0.676129000	3.380299000	-0.558458000
1	-3.853925000	1.900266000	-0.457782000
1	-5.710825000	0.672679000	-1.519416000
1	-5.959397000	-1.763491000	-1.151947000
1	-4.337461000	-2.958191000	0.286938000
1	-2.482003000	-1.723787000	1.348583000

$E = -864.570243$ a.u. (M06-2X/def2-TZVP)

$E = -864.608614$ a.u. (ωB97M-V/def2-TZVP)

$G_{vrt} = 0.289881$ a.u. (M06-2X/def2-TZVP)

$f = 846.7202i$ (M06-2X/def2-TZVP)

FC (IEFPCM, solvent=water)

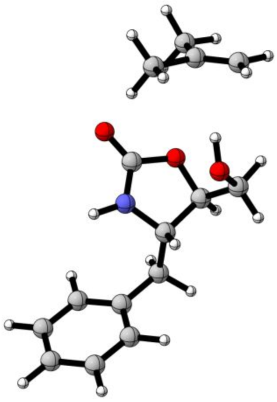

6	-0.787922000	-1.150189000	-0.567184000
7	0.346636000	-0.688145000	-0.009176000
6	0.670141000	0.670682000	-0.383517000
6	-0.596699000	1.054398000	-1.181199000
8	-1.362554000	-0.158302000	-1.287138000
8	-1.275037000	-2.253538000	-0.483655000
6	-4.453000000	-0.306122000	0.669038000
6	-3.807919000	-1.041894000	1.808215000
6	-4.846309000	0.962630000	0.774850000
6	-4.630850000	-1.100098000	-0.590902000
1	0.949949000	-1.290707000	0.526038000
6	1.954540000	0.771753000	-1.209145000
6	-1.437387000	2.110371000	-0.495051000
8	-1.757549000	1.730956000	0.825692000
6	3.154596000	0.302884000	-0.431641000
6	3.776404000	1.148349000	0.486530000
6	4.852408000	0.705838000	1.242639000
6	5.322257000	-0.593931000	1.092809000
6	4.710416000	-1.444561000	0.182342000
6	3.633698000	-0.997144000	-0.574367000
1	0.756788000	1.296012000	0.507857000
1	-0.345775000	1.376115000	-2.193049000
1	-4.399409000	-1.922251000	2.071723000
1	-2.821514000	-1.407065000	1.508644000
1	-3.703194000	-0.413469000	2.691961000
1	-5.306991000	1.480434000	-0.058023000
1	-2.579327000	1.220573000	0.797388000
1	-4.733306000	1.513566000	1.702036000
1	-3.670463000	-1.503804000	-0.918321000
1	-5.289809000	-1.951948000	-0.402154000
1	-5.059344000	-0.497398000	-1.390439000
1	2.079135000	1.816045000	-1.505256000
1	1.833379000	0.176964000	-2.117323000
1	-2.340995000	2.294313000	-1.082830000
1	-0.861325000	3.037119000	-0.453541000
1	3.415488000	2.164072000	0.604145000
1	5.327351000	1.376344000	1.947404000

1	6.162876000	-0.939307000	1.680441000
1	5.072515000	-2.456917000	0.056809000
1	3.162384000	-1.662388000	-1.288967000

E = −864.655389 a.u. (M06-2X/def2-TZVP)

E = −864.688034 a.u. (ωB97M-V/def2-TZVP)

G_{vrt} = 0.287681 a.u. (M06-2X/def2-TZVP)

P

Oxazolidin-2-one (IEFPCM, solvent=water)

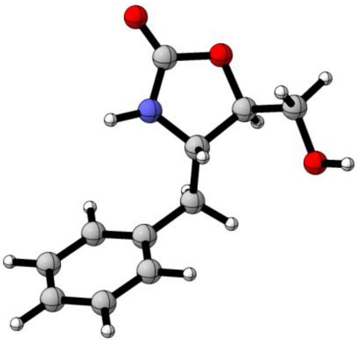

6	-0.771660000	0.191768000	0.053544000
6	-2.225515000	0.409515000	-0.409658000
8	-2.914300000	-0.808164000	-0.098946000
6	-2.031238000	-1.764914000	0.277676000
7	-0.785809000	-1.242542000	0.269279000
8	-2.376728000	-2.887956000	0.551230000
6	-2.916337000	1.557269000	0.291058000
6	0.268472000	0.631683000	-0.972843000
8	-2.177141000	2.716676000	-0.039563000
6	1.668742000	0.353255000	-0.495718000
6	2.303633000	1.233105000	0.380095000
6	3.575560000	0.959536000	0.862618000
6	4.232391000	-0.203161000	0.476159000
6	3.609480000	-1.086622000	-0.394910000
6	2.335815000	-0.808512000	-0.876927000
1	-0.592352000	0.720923000	0.995180000
1	-2.263331000	0.554455000	-1.492125000
1	-0.019711000	-1.750945000	0.680676000
1	-3.952537000	1.628119000	-0.049866000
1	-2.912537000	1.371668000	1.370037000
1	0.128447000	1.700512000	-1.145482000
1	0.074493000	0.107070000	-1.910541000
1	-2.560149000	3.473198000	0.414590000
1	1.795316000	2.142664000	0.680672000
1	4.056763000	1.655229000	1.538127000

1	5.225489000	-0.416380000	0.849753000
1	4.115630000	-1.992387000	-0.703484000
1	1.854447000	-1.498314000	-1.560820000

E = −707.447385 a.u. (M06-2X/def2-TZVP)

E = −707.492204 a.u. (ωB97M-V/def2-TZVP)

G_{vrt} = 0.189011 a.u. (M06-2X/def2-TZVP)

Isobutylene (IEFPCM, solvent=water)

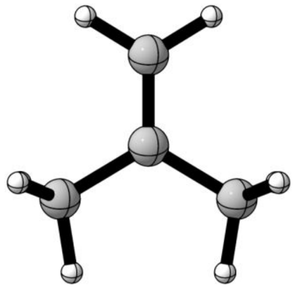

6	0.000000000	1.297716000	-0.670765000
6	0.000000000	0.000000000	0.106714000
6	0.000000000	-1.297716000	-0.670765000
6	0.000000000	0.000000000	1.435545000
1	0.000668000	1.124018000	-1.745239000
1	-0.879026000	1.897059000	-0.429073000
1	0.878311000	1.897693000	-0.428060000
1	-0.000668000	-1.124018000	-1.745239000
1	0.879026000	-1.897059000	-0.429073000
1	-0.878311000	-1.897693000	-0.428060000
1	-0.000048000	-0.925307000	2.000186000
1	0.000048000	0.925307000	2.000186000

E = −157.190169 a.u. (M06-2X/def2-TZVP)

E = −157.175357 a.u. (ωB97M-V/def2-TZVP)

G_{vrt} = 0.081796 a.u. (M06-2X/def2-TZVP)

S6.3 Explicit microsolvation calculations

R

***N*-Boc-epoxide** (IEFPCM, solvent=water)

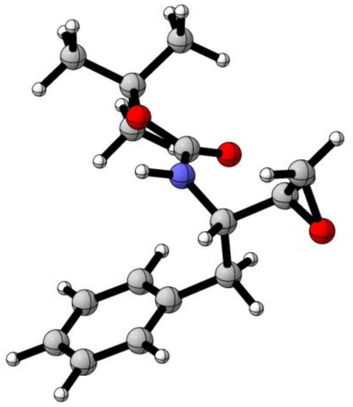

6	0.979894000	0.709523000	-0.026092000
7	-0.119094000	0.888361000	-0.797143000
6	-1.405569000	1.305778000	-0.259439000
6	-1.363764000	2.746223000	0.191638000
8	1.064820000	1.055840000	1.136030000
8	1.947671000	0.111843000	-0.734137000
6	3.238572000	-0.208376000	-0.134493000
6	3.994532000	-0.853365000	-1.285861000
6	3.945661000	1.061566000	0.316975000
6	3.053243000	-1.204423000	1.001398000
1	-0.097648000	0.471692000	-1.713222000
6	-1.922123000	0.398497000	0.871573000
6	-1.706195000	3.826415000	-0.730980000
8	-2.630132000	3.383111000	0.259981000
6	-1.936127000	-1.050551000	0.471491000
6	-2.982846000	-1.571339000	-0.286900000
6	-2.969379000	-2.896673000	-0.701079000
6	-1.903370000	-3.721812000	-0.363310000
6	-0.854621000	-3.213545000	0.392584000
6	-0.873440000	-1.887381000	0.805944000
1	-2.107505000	1.246586000	-1.093462000
1	-0.704038000	2.958421000	1.026613000
1	4.989951000	-1.146812000	-0.953261000
1	3.466719000	-1.740430000	-1.637131000
1	4.094828000	-0.151873000	-2.114528000
1	4.967864000	0.813007000	0.605031000
1	3.442089000	1.516797000	1.165716000
1	3.986598000	1.778818000	-0.504179000
1	2.490392000	-2.071159000	0.650116000
1	4.032684000	-1.546099000	1.338350000
1	2.531334000	-0.754143000	1.842488000
1	-2.930112000	0.737571000	1.117991000

1	-1.293672000	0.539574000	1.750672000
1	-1.977830000	3.579077000	-1.751240000
1	-1.302227000	4.818203000	-0.568804000
1	-3.818105000	-0.931874000	-0.550914000
1	-3.792871000	-3.286900000	-1.285588000
1	-1.892367000	-4.755329000	-0.684392000
1	-0.021748000	-3.850455000	0.663104000
1	-0.056358000	-1.492772000	1.400958000

E = −864.641805 a.u. (M06-2X/def2-TZVP)

E = −864.668315 a.u. (ωB97M-V/def2-TZVP)

G_{vrt} = 0.293266 a.u. (M06-2X/def2-TZVP)

PRC$_1$ (IEFPCM, solvent=water)

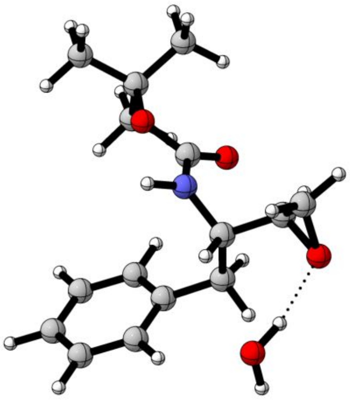

6	1.143033000	-0.855076000	0.015855000
7	0.003036000	-0.809362000	0.747893000
6	-1.320423000	-0.935007000	0.157126000
6	-1.572422000	-2.352484000	-0.298104000
8	1.196598000	-1.212580000	-1.144489000
8	2.184834000	-0.465180000	0.761462000
6	3.534977000	-0.412202000	0.210407000
6	4.362784000	0.069076000	1.391958000
6	3.987556000	-1.799126000	-0.223016000
6	3.594847000	0.599283000	-0.925349000
1	0.076289000	-0.402162000	1.665687000
6	-1.579718000	0.059163000	-0.987920000
6	-2.219453000	-3.317904000	0.587620000
8	-2.947436000	-2.687078000	-0.470047000
6	-1.312384000	1.475248000	-0.561038000
6	-2.247948000	2.170577000	0.203071000
6	-1.983474000	3.459977000	0.644690000
6	-0.774569000	4.070640000	0.330973000
6	0.165621000	3.384596000	-0.426925000
6	-0.103772000	2.094978000	-0.869032000
1	-2.027480000	-0.713897000	0.959406000

1	-0.925739000	-2.717389000	-1.088462000
1	5.408170000	0.156133000	1.096692000
1	4.011667000	1.044576000	1.729500000
1	4.290203000	-0.637271000	2.219381000
1	5.048231000	-1.761756000	-0.474371000
1	3.432885000	-2.145192000	-1.091168000
1	3.854671000	-2.508577000	0.595152000
1	3.208350000	1.562881000	-0.588368000
1	4.634344000	0.733867000	-1.226924000
1	3.021299000	0.263417000	-1.786076000
1	-2.624079000	-0.056758000	-1.284343000
1	-0.953342000	-0.205263000	-1.839373000
1	-2.506952000	-3.003234000	1.584617000
1	-2.049159000	-4.377358000	0.446229000
1	-3.188345000	1.689268000	0.450707000
1	-2.721393000	3.990452000	1.233114000
1	-0.568099000	5.076108000	0.674304000
1	1.109311000	3.853565000	-0.675996000
1	0.629575000	1.559681000	-1.463272000
8	-4.671030000	-0.684909000	0.609645000
1	-5.154380000	-0.220687000	-0.080283000
1	-4.178951000	-1.387342000	0.154117000

E = −941.084180 a.u. (M06-2X/def2-TZVP)

E = −941.118602 a.u. (ωB97M-V/def2-TZVP)

G_{vrt} = 0.312774 a.u. (M06-2X/def2-TZVP)

Water (IEFPCM, solvent=water)

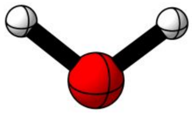

8	0.000000000	0.117404000	0.000000000
1	0.762157000	-0.469636000	0.000000000
1	-0.762157000	-0.469594000	0.000000000

E = −76.433708 a.u. (M06-2X/def2-TZVP)

E = −76.442823 a.u. (ωB97M-V/def2-TZVP)

G_{vrt} = 0.003093 a.u. (M06-2X/def2-TZVP)

TS_1 (IEFPCM, solvent=water)

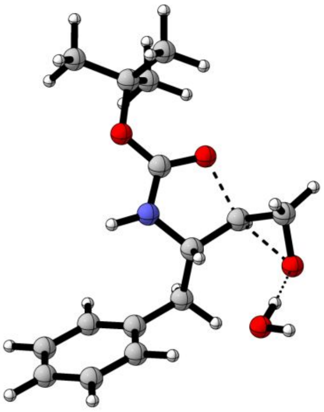

6	1.574507000	-0.067531000	-0.036365000
7	0.272825000	-0.302123000	-0.142823000
6	-0.595539000	0.836980000	0.127066000
6	0.313467000	1.945216000	0.651547000
8	1.931553000	1.065588000	0.350315000
8	2.370396000	-1.055343000	-0.344947000
6	3.840298000	-0.946488000	-0.236207000
6	4.301380000	-2.321814000	-0.685058000
6	4.344118000	0.130537000	-1.182117000
6	4.226830000	-0.689217000	1.210464000
1	-0.083620000	-1.184122000	-0.478370000
6	-1.701990000	0.454072000	1.113233000
6	0.301546000	3.229486000	-0.042476000
8	-0.944110000	3.415164000	0.552858000
6	-2.545228000	-0.673683000	0.578166000
6	-2.390467000	-1.975270000	1.047424000
6	-3.146913000	-3.014888000	0.517375000
6	-4.066120000	-2.761195000	-0.490893000
6	-4.225325000	-1.464183000	-0.967367000
6	-3.469470000	-0.427748000	-0.438224000
1	-1.051724000	1.178699000	-0.804918000
1	0.469525000	1.997965000	1.719531000
1	5.389195000	-2.367442000	-0.647779000
1	3.895584000	-3.092492000	-0.029889000
1	3.977257000	-2.517632000	-1.707090000
1	5.433409000	0.089374000	-1.205611000
1	4.041469000	1.123321000	-0.858013000
1	3.972353000	-0.049848000	-2.191302000
1	3.775846000	-1.439530000	1.860792000
1	5.310780000	-0.766047000	1.298783000
1	3.922323000	0.302198000	1.537125000
1	-2.308270000	1.345375000	1.271682000
1	-1.244741000	0.171180000	2.063752000
1	0.287079000	3.149021000	-1.136721000
1	1.078594000	3.933370000	0.269724000
1	-1.675815000	-2.175736000	1.837696000

1	-3.018237000	-4.021222000	0.894986000
1	-4.657590000	-3.568499000	-0.903111000
1	-4.942698000	-1.261270000	-1.752531000
1	-3.585974000	0.586825000	-0.806138000
1	-2.143422000	3.125347000	-0.453376000
8	-2.887249000	2.870935000	-1.081300000
1	-3.082386000	3.659635000	-1.594279000

$E = -941.030197$ a.u. (M06-2X/def2-TZVP)

$E = -941.073205$ a.u. (ωB97M-V/def2-TZVP)

$G_{vrt} = 0.311366$ a.u. (M06-2X/def2-TZVP)

$f = 619.3281i$ (M06-2X/def2-TZVP)

INT (IEFPCM, solvent=water)

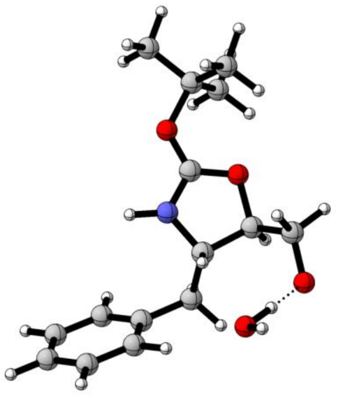

6	1.520490000	-0.312695000	-0.062736000
7	0.234877000	-0.525142000	-0.171448000
6	-0.536875000	0.670104000	0.189991000
6	0.579846000	1.575569000	0.741931000
8	1.828632000	0.852342000	0.414655000
8	2.388407000	-1.186537000	-0.405800000
6	3.868410000	-0.996133000	-0.251489000
6	4.406015000	-2.311211000	-0.780678000
6	4.309269000	0.170803000	-1.114715000
6	4.192858000	-0.815540000	1.219264000
1	-0.149806000	-1.368354000	-0.572818000
6	-1.652293000	0.365943000	1.179442000
6	0.572679000	2.966780000	0.127977000
8	-0.595512000	3.584810000	0.479977000
6	-2.668967000	-0.577764000	0.591213000
6	-2.744140000	-1.903261000	1.008530000
6	-3.661562000	-2.776024000	0.432991000
6	-4.511293000	-2.328745000	-0.568573000

6	-4.438625000	-1.006339000	-0.994422000
6	-3.522867000	-0.135977000	-0.420802000
1	-0.966247000	1.111532000	-0.711882000
1	0.550792000	1.633854000	1.829995000
1	5.493507000	-2.298215000	-0.720954000
1	4.031865000	-3.144204000	-0.186375000
1	4.113535000	-2.452889000	-1.820588000
1	5.399088000	0.192742000	-1.122456000
1	3.950474000	1.122235000	-0.729297000
1	3.962091000	0.037665000	-2.139345000
1	3.768906000	-1.631033000	1.805270000
1	5.276791000	-0.840491000	1.332084000
1	3.831509000	0.135628000	1.602981000
1	-2.111430000	1.326084000	1.423418000
1	-1.223242000	-0.050731000	2.092899000
1	0.698382000	2.831911000	-0.966211000
1	1.477296000	3.494201000	0.477619000
1	-2.083491000	-2.254376000	1.793130000
1	-3.711636000	-3.803560000	0.769848000
1	-5.227421000	-3.005676000	-1.016303000
1	-5.100151000	-0.653659000	-1.775677000
1	-3.453860000	0.895981000	-0.752585000
1	-1.643368000	3.264333000	-0.443214000
8	-2.413047000	2.989794000	-1.113349000
1	-2.473243000	3.696690000	-1.760814000

E = −941.050746 a.u. (M06-2X/def2-TZVP)

E = −941.095988 a.u. (ωB97M-V/def2-TZVP)

G_{vrt} = 0.314661 a.u. (M06-2X/def2-TZVP)

PRC$_2$ (IEFPCM, solvent=water)

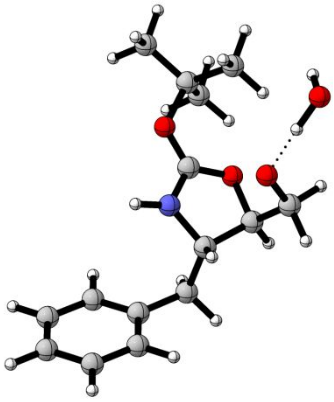

6	0.902035000	-0.469380000	0.089627000
7	-0.311792000	-0.174097000	-0.297641000
6	-0.826026000	0.983319000	0.432635000

6	0.442924000	1.424390000	1.189866000
8	1.341213000	0.262758000	1.062960000
8	1.564753000	-1.436618000	-0.422306000
6	2.994823000	-1.722251000	-0.082092000
6	3.312477000	-2.879171000	-1.009016000
6	3.831011000	-0.500260000	-0.412943000
6	3.085260000	-2.138439000	1.374047000
1	-0.773432000	-0.638957000	-1.064796000
6	-2.004866000	0.615633000	1.331290000
6	1.144074000	2.595869000	0.516438000
8	1.391769000	2.330280000	-0.797302000
6	-3.172452000	0.092779000	0.537632000
6	-4.039337000	0.974426000	-0.106616000
6	-5.089993000	0.497900000	-0.877596000
6	-5.287209000	-0.871057000	-1.017254000
6	-4.429318000	-1.757488000	-0.380793000
6	-3.378722000	-1.276691000	0.391817000
1	-1.104693000	1.754977000	-0.285600000
1	0.274213000	1.563140000	2.255879000
1	4.347294000	-3.182443000	-0.855149000
1	2.663210000	-3.728268000	-0.797375000
1	3.185258000	-2.581341000	-2.049259000
1	4.882203000	-0.766899000	-0.300556000
1	3.608184000	0.331594000	0.252542000
1	3.659573000	-0.191592000	-1.444688000
1	2.385363000	-2.948041000	1.581202000
1	4.095443000	-2.502782000	1.561350000
1	2.890122000	-1.307592000	2.047697000
1	-2.290406000	1.515995000	1.879246000
1	-1.674837000	-0.128381000	2.059588000
1	2.057652000	2.800714000	1.107719000
1	0.471403000	3.461298000	0.688804000
1	-3.891648000	2.043024000	0.003067000
1	-5.757990000	1.194867000	-1.367192000
1	-6.107860000	-1.243607000	-1.616380000
1	-4.578539000	-2.824842000	-0.481449000
1	-2.714164000	-1.971601000	0.892488000
8	3.771018000	2.887148000	-1.315208000
1	4.318679000	2.176444000	-0.974952000
1	2.779406000	2.626948000	-1.096108000

E = −941.053031 a.u. (M06-2X/def2-TZVP)

E = −941.100831 a.u. (ωB97M-V/def2-TZVP)

G_{vrt} = 0.314548 a.u. (M06-2X/def2-TZVP)

$TS_{1.2}$' (IEFPCM, solvent=water)

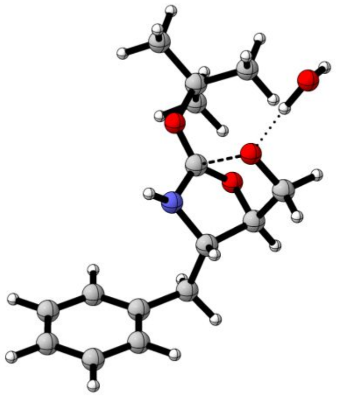

6	-0.933772000	-0.272898000	-0.046104000
7	0.346521000	-0.095644000	0.317683000
6	0.880873000	1.013276000	-0.489941000
6	-0.385733000	1.411389000	-1.273146000
8	-1.153716000	0.172227000	-1.265938000
8	-1.657005000	-1.164759000	0.528316000
6	-3.006523000	-1.557128000	0.045856000
6	-3.454489000	-2.537185000	1.114649000
6	-3.918514000	-0.346179000	-0.002546000
6	-2.867116000	-2.247700000	-1.300393000
1	0.580462000	-0.176793000	1.295652000
6	2.050664000	0.562546000	-1.361911000
6	-1.270093000	2.336308000	-0.435957000
8	-1.594565000	1.632403000	0.701694000
6	3.211049000	0.080182000	-0.533788000
6	4.111576000	0.991874000	0.015243000
6	5.159015000	0.560321000	0.817216000
6	5.319713000	-0.794258000	1.083519000
6	4.428019000	-1.710921000	0.542523000
6	3.380876000	-1.274612000	-0.260040000
1	1.183080000	1.833900000	0.162479000
1	-0.213212000	1.705635000	-2.303749000
1	-4.450580000	-2.904051000	0.869766000
1	-2.771787000	-3.385252000	1.166008000
1	-3.490132000	-2.048589000	2.087980000
1	-4.914787000	-0.689595000	-0.284515000
1	-3.579380000	0.387189000	-0.731728000
1	-3.971868000	0.129717000	0.975008000
1	-2.120233000	-3.040092000	-1.244247000
1	-3.826453000	-2.698310000	-1.555852000
1	-2.594656000	-1.549040000	-2.087745000
1	2.354622000	1.410759000	-1.978977000
1	1.702799000	-0.229917000	-2.028140000
1	-2.149431000	2.611262000	-1.044012000
1	-0.703259000	3.261122000	-0.241892000
1	3.992591000	2.049338000	-0.193480000

1	5.852532000	1.280803000	1.231710000
1	6.137514000	-1.132930000	1.706310000
1	4.548096000	-2.767946000	0.742972000
1	2.687904000	-1.992441000	-0.682952000
8	-3.784074000	2.685806000	1.569878000
1	-4.457600000	2.484222000	0.915170000
1	-2.940256000	2.235014000	1.237779000

$E = -941.046260$ a.u. (M06-2X/def2-TZVP)

$E = -941.089556$ a.u. (ωB97M-V/def2-TZVP)

$G_{vrt} = 0.317067$ a.u. (M06-2X/def2-TZVP)

$f = 295.6825i$ (M06-2X/def2-TZVP)

INT’ (IEFPCM, solvent=water)

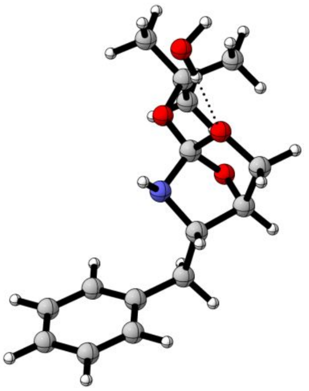

6	-1.114297000	-0.188575000	0.046279000
7	0.274953000	-0.128755000	-0.359248000
6	0.936738000	-1.123482000	0.523480000
6	-0.269376000	-1.588454000	1.355855000
8	-1.033370000	-0.376188000	1.434812000
8	-1.805012000	0.875917000	-0.391623000
6	-3.092857000	1.258183000	0.183426000
6	-3.698049000	2.145859000	-0.892645000
6	-4.002959000	0.069331000	0.457867000
6	-2.834354000	2.058703000	1.453568000
1	0.363822000	-0.366074000	-1.340166000
6	2.034190000	-0.483666000	1.372554000
6	-1.198848000	-2.426234000	0.473587000
8	-1.704693000	-1.421548000	-0.415600000
6	3.164007000	0.042440000	0.530074000
6	4.140271000	-0.825549000	0.042263000
6	5.168393000	-0.360007000	-0.765942000
6	5.235530000	0.987532000	-1.099904000

6	4.268968000	1.861497000	-0.620029000
6	3.241409000	1.389999000	0.188147000
1	1.353448000	-1.951360000	-0.053000000
1	-0.054937000	-1.984436000	2.342700000
1	-4.654775000	2.541332000	-0.551634000
1	-3.032621000	2.981415000	-1.111889000
1	-3.859212000	1.573296000	-1.806608000
1	-4.957457000	0.453845000	0.819958000
1	-3.587039000	-0.590899000	1.216979000
1	-4.182034000	-0.502208000	-0.451565000
1	-2.144865000	2.877504000	1.244712000
1	-3.773688000	2.480288000	1.813808000
1	-2.412084000	1.429492000	2.234521000
1	2.410889000	-1.237709000	2.068257000
1	1.589175000	0.323809000	1.956947000
1	-2.023731000	-2.855907000	1.042608000
1	-0.678738000	-3.202936000	-0.084967000
1	4.095298000	-1.877115000	0.304108000
1	5.920160000	-1.047819000	-1.131805000
1	6.037978000	1.353679000	-1.727207000
1	4.315064000	2.913103000	-0.873392000
1	2.488679000	2.074382000	0.560651000
8	-3.020063000	-1.363361000	-2.947410000
1	-3.915953000	-1.684647000	-2.811369000
1	-2.589542000	-1.423785000	-2.079824000

E = −941.071652 a.u. (M06-2X/def2-TZVP)

E = −941.113324 a.u. (ωB97M-V/def2-TZVP)

G_{vrt} = 0.31701 a.u. (M06-2X/def2-TZVP)

TS$_2$ (IEFPCM, solvent=water)

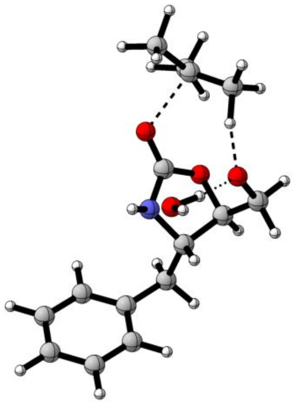

6	-1.045626000	-0.549918000	-0.243192000

7	0.218392000	-0.305225000	0.144808000
6	0.744441000	0.867258000	-0.550515000
6	-0.514043000	1.363979000	-1.281370000
8	-1.362121000	0.185514000	-1.312345000
8	-1.825035000	-1.359610000	0.248298000
6	-3.945234000	-0.902148000	0.131388000
6	-4.185420000	-1.732316000	1.330404000
6	-4.022491000	0.535024000	0.254899000
6	-4.096671000	-1.529071000	-1.197393000
1	0.440919000	-0.444180000	1.122067000
6	1.904940000	0.509592000	-1.479684000
6	-1.272484000	2.424548000	-0.484799000
8	-1.641940000	1.954798000	0.754178000
6	3.076100000	-0.051694000	-0.720170000
6	3.959680000	0.800088000	-0.058311000
6	5.016380000	0.291292000	0.683945000
6	5.204695000	-1.082776000	0.776398000
6	4.330432000	-1.940509000	0.122221000
6	3.273648000	-1.426351000	-0.619496000
1	1.064298000	1.604126000	0.188132000
1	-0.321324000	1.658738000	-2.311608000
1	-5.276325000	-1.790571000	1.429914000
1	-3.795044000	-2.739344000	1.217451000
1	-3.794546000	-1.256695000	2.227312000
1	-4.640333000	0.815203000	1.109167000
1	-4.350900000	1.012087000	-0.666926000
1	-2.993175000	0.996371000	0.499375000
1	-3.748205000	-2.557597000	-1.213065000
1	-5.177136000	-1.522224000	-1.392535000
1	-3.617124000	-0.934697000	-1.971246000
1	2.201181000	1.416163000	-2.012360000
1	1.550307000	-0.212165000	-2.219351000
1	-2.146523000	2.719500000	-1.092262000
1	-0.613732000	3.310322000	-0.438206000
1	3.819136000	1.873016000	-0.130756000
1	5.695800000	0.967110000	1.187579000
1	6.029658000	-1.481363000	1.352576000
1	4.471140000	-3.011947000	0.187491000
1	2.593202000	-2.098492000	-1.129041000
8	-0.021916000	1.111180000	2.549870000
1	-0.041124000	1.713278000	3.297475000
1	-0.640872000	1.502900000	1.839200000

$E = -941.024229$ a.u. (M06-2X/def2-TZVP)

$E = -941.068676$ a.u. (ωB97M-V/def2-TZVP)

$G_{vrt} = 0.310721$ a.u. (M06-2X/def2-TZVP)

f = 286.5812i (M06-2X/def2-TZVP)

FC (IEFPCM, solvent=water)

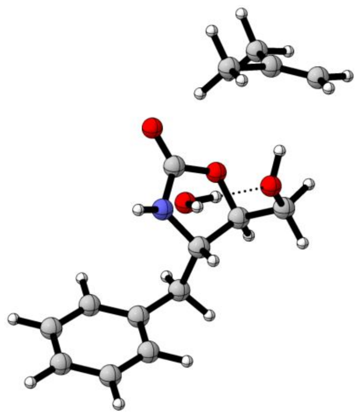

6	-0.756430000	1.290488000	-0.584422000
7	0.384946000	0.578087000	-0.485579000
6	0.707687000	0.205651000	0.875931000
6	-0.539230000	0.727715000	1.622071000
8	-1.240400000	1.529946000	0.658244000
8	-1.301482000	1.704884000	-1.580067000
6	-4.461165000	-0.341770000	-0.509931000
6	-3.780164000	-0.882055000	-1.734287000
6	-4.884586000	-1.134058000	0.475396000
6	-4.626079000	1.148384000	-0.468202000
1	0.733687000	0.070633000	-1.283733000
6	2.015918000	0.832889000	1.361517000
6	-1.450015000	-0.389088000	2.085743000
8	-1.762322000	-1.253940000	1.005153000
6	3.191047000	0.352079000	0.554277000
6	3.781564000	-0.879840000	0.833054000
6	4.833652000	-1.353756000	0.061782000
6	5.310196000	-0.600283000	-1.004635000
6	4.728441000	0.627171000	-1.292226000
6	3.675472000	1.098170000	-0.517204000
1	0.774084000	-0.881542000	0.959086000
1	-0.271386000	1.369414000	2.461211000
1	-4.348228000	-0.615589000	-2.628995000
1	-2.790880000	-0.428664000	-1.844345000
1	-3.673210000	-1.965675000	-1.695850000
1	-4.772592000	-2.211249000	0.420646000
1	-5.371024000	-0.727989000	1.354310000
1	-2.677970000	-1.092183000	0.729793000
1	-3.653509000	1.635443000	-0.569762000
1	-5.237509000	1.478191000	-1.312258000
1	-5.098865000	1.476477000	0.456339000
1	2.145491000	0.568969000	2.413723000
1	1.925790000	1.919493000	1.295516000

1	-2.362388000	0.031597000	2.513557000
1	-0.935828000	-0.963708000	2.859156000
1	3.415217000	-1.469512000	1.666213000
1	5.284505000	-2.309976000	0.294662000
1	6.132140000	-0.967287000	-1.605512000
1	5.095311000	1.221124000	-2.119585000
1	3.224714000	2.057558000	-0.742960000
8	-0.161733000	-2.161025000	-1.211924000
1	-0.263175000	-3.114528000	-1.285406000
1	-0.719336000	-1.896710000	-0.461175000

$E = -941.098338$ a.u. (M06-2X/def2-TZVP)

$E = -941.136509$ a.u. (ωB97M-V/def2-TZVP)

$G_{vrt} = 0.30791$ a.u. (M06-2X/def2-TZVP)

P

Oxazolidin-2-one (IEFPCM, solvent=water)

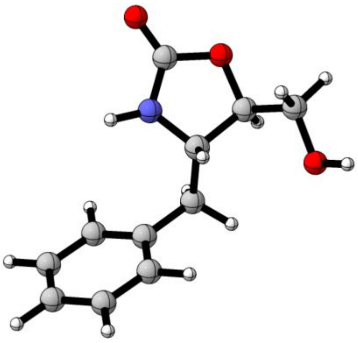

6	-0.771660000	0.191768000	0.053544000
6	-2.225515000	0.409515000	-0.409658000
8	-2.914300000	-0.808164000	-0.098946000
6	-2.031238000	-1.764914000	0.277676000
7	-0.785809000	-1.242542000	0.269279000
8	-2.376728000	-2.887956000	0.551230000
6	-2.916337000	1.557269000	0.291058000
6	0.268472000	0.631683000	-0.972843000
8	-2.177141000	2.716676000	-0.039563000
6	1.668742000	0.353255000	-0.495718000
6	2.303633000	1.233105000	0.380095000
6	3.575560000	0.959536000	0.862618000
6	4.232391000	-0.203161000	0.476159000
6	3.609480000	-1.086622000	-0.394910000
6	2.335815000	-0.808512000	-0.876927000
1	-0.592352000	0.720923000	0.995180000

1	-2.263331000	0.554455000	-1.492125000
1	-0.019711000	-1.750945000	0.680676000
1	-3.952537000	1.628119000	-0.049866000
1	-2.912537000	1.371668000	1.370037000
1	0.128447000	1.700512000	-1.145482000
1	0.074493000	0.107070000	-1.910541000
1	-2.560149000	3.473198000	0.414590000
1	1.795316000	2.142664000	0.680672000
1	4.056763000	1.655229000	1.538127000
1	5.225489000	-0.416380000	0.849753000
1	4.115630000	-1.992387000	-0.703484000
1	1.854447000	-1.498314000	-1.560820000

$E = -707.447385$ a.u. (M06-2X/def2-TZVP)

$E = -707.492204$ a.u. (ωB97M-V/def2-TZVP)

$G_{vrt} = 0.189011$ a.u. (M06-2X/def2-TZVP)

Isobutylene (IEFPCM, solvent=water)

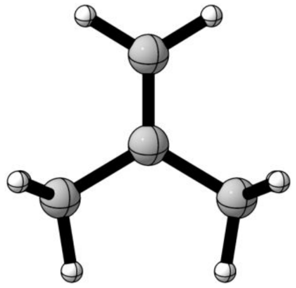

6	0.000000000	1.297716000	-0.670765000
6	0.000000000	0.000000000	0.106714000
6	0.000000000	-1.297716000	-0.670765000
6	0.000000000	0.000000000	1.435545000
1	0.000668000	1.124018000	-1.745239000
1	-0.879026000	1.897059000	-0.429073000
1	0.878311000	1.897693000	-0.428060000
1	-0.000668000	-1.124018000	-1.745239000
1	0.879026000	-1.897059000	-0.429073000
1	-0.878311000	-1.897693000	-0.428060000
1	-0.000048000	-0.925307000	2.000186000
1	0.000048000	0.925307000	2.000186000

$E = -157.190169$ a.u. (M06-2X/def2-TZVP)

$E = -157.175357$ a.u. (ωB97M-V/def2-TZVP)

G_{vrt} = 0.081796 a.u. (M06-2X/def2-TZVP)

Water (IEFPCM, solvent=water)

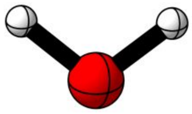

8	0.000000000	0.117404000	0.000000000
1	0.762157000	-0.469636000	0.000000000
1	-0.762157000	-0.469594000	0.000000000

E = −76.433708 a.u. (M06-2X/def2-TZVP)

E = −76.442823 a.u. (ωB97M-V/def2-TZVP)

G_{vrt} = 0.003093 a.u. (M06-2X/def2-TZVP)
